# Processing of ellipsis with garden-path antecedents in French and German: Evidence from eye tracking

**DOI:** 10.1371/journal.pone.0198620

**Published:** 2018-06-13

**Authors:** Dario Paape, Barbara Hemforth, Shravan Vasishth

**Affiliations:** 1 Department of Linguistics, University of Potsdam, Potsdam, Germany; 2 Laboratoire de Linguistique Formelle, Université Paris Diderot, Paris, France; William & Mary, UNITED STATES

## Abstract

In a self-paced reading study on German sluicing, Paape (Paape, 2016) found that reading times were shorter at the ellipsis site when the antecedent was a temporarily ambiguous garden-path structure. As a post-hoc explanation of this finding, Paape assumed that the antecedent’s memory representation was reactivated during syntactic reanalysis, making it easier to retrieve. In two eye tracking experiments, we subjected the reactivation hypothesis to further empirical scrutiny. Experiment 1, carried out in French, showed no evidence in favor in the reactivation hypothesis. Instead, results for one out of the three types of garden-path sentences that were tested suggest that subjects sometimes failed to resolve the temporary ambiguity in the antecedent clause, and subsequently failed to resolve the ellipsis. The results of Experiment 2, a conceptual replication of Paape’s (Paape, 2016) original study carried out in German, are compatible with the reactivation hypothesis, but leave open the possibility that the observed speedup for ambiguous antecedents may be due to occasional retrievals of an incorrect structure.

## Introduction

Ellipsis has received considerable attention in both theoretical linguistics and experimental psycholinguistics. Part of the appeal of ellipsis is that meaning is essentially generated from nothing. In (1), the second clause attains the meaning *but I don’t know what Jane was supposed to do* without the lexical items being present. This particular type of ellipsis, in which only a wh-pronoun is left behind, is known as ‘sluicing’ [2].

(1) Jane was supposed to do something, but I don’t know what.

The generation of meaning from silence is difficult to capture using traditional phrase structure rules, in which syntactic phrases are projected from a layer of terminal symbols taken from the mental lexicon. Having the word *what* project a whole clause would violate the principle of syntactic headedness, as *what* is not of the required syntactic category, not to mention that invisible terminal symbols would be needed to carry the meaning conveyed by the ellipsis [3]. An alternative view would assume that there is an invisible pro-form at the ellipsis gap, much like a trace left behind by syntactic movement, that is co-indexed with the antecedent clause (e.g. [4]).

From a processing perspective, the ‘something from nothing’ challenge becomes less of a problem: upon encountering the wh-pronoun at the end of the clause, the reader notices that there must be a gap, and the parser simply has to access the relevant material from the antecedent in order to fill in the elided part of the second clause. There is a long-standing debate about the exact nature of the ‘filling in’ process, which has been claimed to produce syntactic structure at the ellipsis site [5, 6] or involve only a transfer of meaning [4]. Both accounts, however, lead to empirical problems, as noted by Merchant [7], inter alia.

An issue that is sometimes framed as being part of the syntax/semantics dichotomy, but is, in fact, orthogonal to it [10] concerns the mechanism by which information from the antecedent is transferred to the ellipsis site. There is converging evidence from processing studies on ellipsis that neither the size of the antecedent nor the distance between antecedent and gap influence the time it takes to resolve the ellipsis [6, 11–13] (see, however, [14] for a diverging result). The observed pattern suggests that the parser has direct access to the antecedent’s representation in memory, without having to initiate a time-consuming search process, and that all information from the antecedent is accessed at once, so that processing is not slowed for bigger as compared to smaller antecedents.

A recent study by Paape [1] investigated whether ellipsis is processed differently depending on whether the antecedent contains a temporary syntactic ambiguity or not. A German example stimulus from the study, adapted slightly in favor of brevity, is shown in (2).

(2)
  - **Locally ambiguous conditions**
    Eine Sympathisantin der Opposition hatte/n die Rebellen unterstützt, aber niemand weiß, wie, …
    A sympathizer.fem.nom|acc of the opposition had.sg/pl the rebels.nom|acc supported, but nobody knows how
    ‘The rebels had (been) supported (by) a sympathizer of the opposition, but nobody knows how, …’
  - **Control conditions**
    Ein/en Sympathisant/en der Opposition hatte/n die Rebellen unterstützt, aber niemand weiß, wie, …
    A.nom/acc sympathizer.masc.acc of the opposition had.sg/pl the rebels.nom|acc supported, but nobody knows how
    ‘The rebels had (been) supported (by) a sympathizer of the opposition, but nobody knows how, …’

In (2a), the first noun phrase *Eine Sympathisantin …*, ‘a sympathizer …’, is initially ambiguous between nominative and accusative marking, which is always the case for feminine but not masculine singular noun phrases in German. Native speakers prefer to adopt a subject reading for this noun phrase [15, 16]. As the finite verb in German agrees with the subject in number, readers are forced to reanalyze when the plural-marked auxiliary *hatten*, ‘had.pl’, arrives to indicate that the sentence has non-canonical OVS order, with the case-ambiguous post-verbal NP being the subject. In the control condition, the auxiliary bears singular marking and thus agrees with the initial noun phrase, allowing an SVO reading. In a self-paced reading experiment, Paape [1] found that reading times were increased at the second noun phrase in the OVS version of (2a) compared to the SVO version. No such effect was observed in the two additional control sentences in (2b), where the first noun phrase carries overt case marking. Taken together, the findings indicate that readers experienced a garden path upon disambiguation in the ambiguous OVS condition.

The use of potential garden-path sentences as antecedents for ellipsis was intended as a more stringent test of the assumption that the parser has direct access to a structured representation of the antecedent. If reanalysis of the ambiguous structure is successful—a reasonable assumption given that OVS constructions are not particularly rare in German—the parser should have a fully specified antecedent structure available when the ellipsis site is encountered. As this should also be the case in the control conditions, no difference in processing times is predicted. Alternatively, the parser may only have access to a (partly) unstructured antecedent representation, that is, a loose collection of constituents or words, and would have to compute the necessary syntactic (or semantic) structure *again* at the ellipsis site, which would possibly result in a reappearance of the garden-path effect.

**Fig 1:**
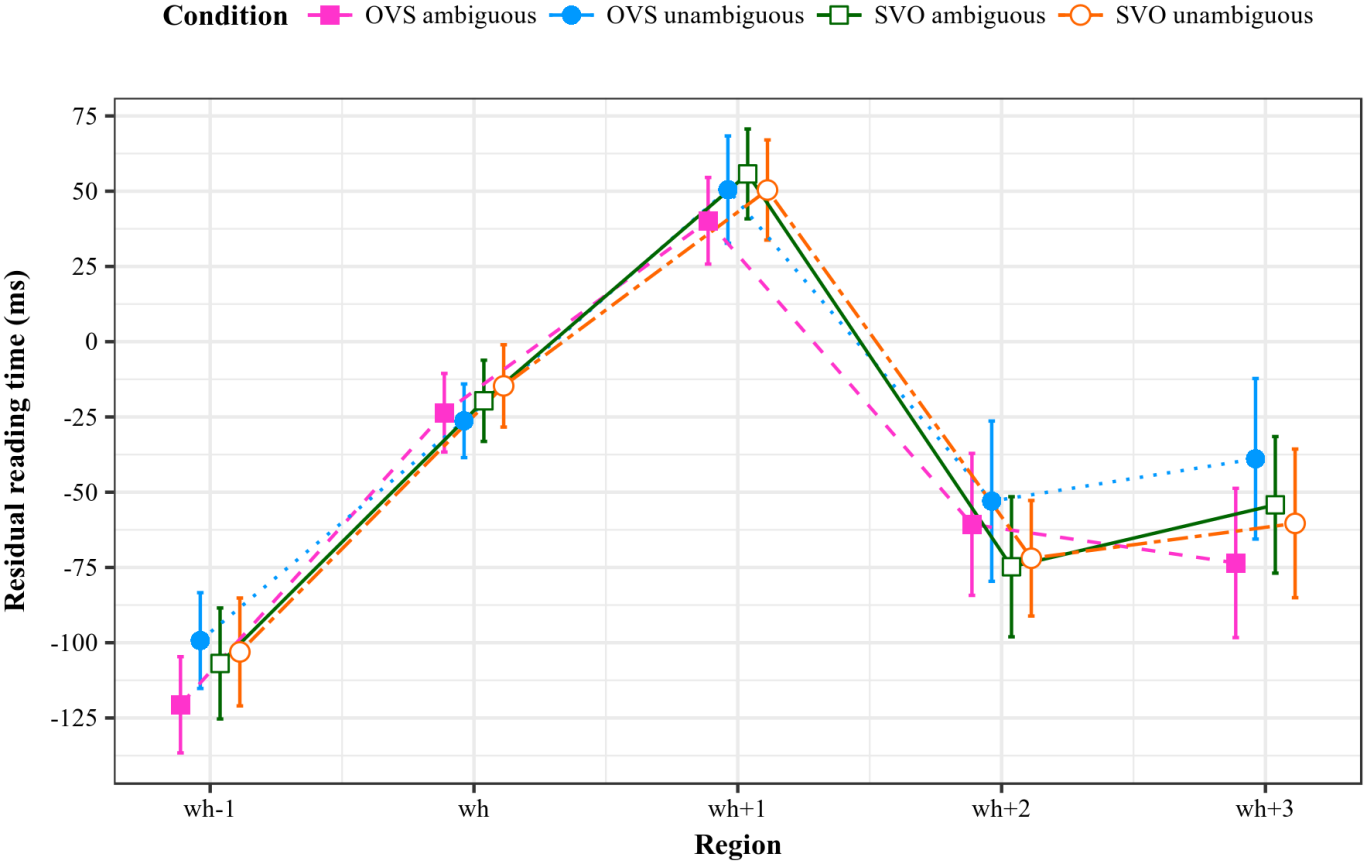
Residual reading times for regions wh−1 through wh+3 from Paape’s original study [1].

Apart from replicating the classic garden-path effect in the antecedent clause, Paape’s study yielded two main results: sentences with non-canonical OVS order in the antecedent showed longer reading times in a spillover region two regions downstream from the ellipsis site, independently of ambiguity. In the spillover region three regions downstream from the ellipsis site, there was an interaction between case marking on the initial noun phrase and antecedent word order, such that sentences with ambiguous OVS antecedents were processed faster compared to both their ambiguous SVO counterparts and control sentences. Furthermore, sentences with unambiguous OVS antecedents showed the longest reading times at this position. Fig 1 reproduces Paape’s [1, p. 7] summary of the results based on the original data.

Paape’s findings are incompatible with the view that the structure of the antecedent has to be recomputed at the ellipsis site: such an account would have predicted the opposite pattern, namely increased reading times for ambiguous OVS sentences at the ellipsis site, given that the garden-path effect should have been experienced again. The results are, however, in line with the retrieval of a stored structure if additional assumptions are made.

In the cue-based retrieval model of parsing developed by Lewis and Vasishth [17], syntactic chunks with varying activation levels are retrieved from memory in order to form new structures. Each chunk is assumed to have an associated activation level that will decrease with time unless the chunk is retrieved, that is, unless it is needed for syntactic computation. Any chunk that *is* retrieved receives a boost to its activation level. Even though this boost decays over time, it is permanent in the sense that the activation of a chunk that has been retrieved *n* times will not decay below the activation level of a chunk that has been retrieved *n* − 1 times.

Under the Lewis & Vasishth model, a sluicing gap would set a retrieval probe for a fully parsed clause in memory. The latency of the retrieval decreases with the target’s activation, which is determined by its retrieval history (see above) and the match between the target’s feature specification and the features cued for by the probe. Paape [1] suggests that the reanalysis of ambiguous OVS antecedents indexed by the garden-path effect in the antecedent clause involves additional retrievals of the corresponding memory chunk. This results in an activation boost, leading to faster retrieval at the ellipsis site later on. As no reanalysis and therefore no reactivation is assumed for ambiguous SVO or unambiguous antecedents, their activation levels are predicted to be lower upon retrieval, resulting in slowed processing compared to ambiguous OVS antecedents.

However, OVS antecedents also appeared to slow down processing at the ellipsis site, as indicated by the increased reading times at the second spillover region and the high reading times for sentences with unambiguous OVS antecedents at the third spillover region. In order to explain the finding, Paape [1] notes that there is a form mismatch between antecedent and ellipsis site in (2): the wh-pronoun *wie*, ‘how’, marks the beginning of a subordinate clause—the complement of *weiß*, ‘knows’—which in German would canonically show SOV word order if there was no ellipsis, see (3) below. The requirement for a complement clause headed by *dass*, ‘that’, to be verb-final is absolute, unless the embedded verb takes a clausal complement as well. Note that an unelided utterance would likely use a definite rather than an indefinite article, and most likely drop the modifier.

(3)
  [Eine Sympathisantin der Opposition]_O_ hatten_V_ [die Rebellen]_S_ unterstützt, aber niemand weiß, wie [die Rebellen]_S_ [die Sympathisantin]_O_ unterstützt hatten_V_.
    A sympathizer.fem.nom/acc of the opposition had.pl the rebels supported, but nobody knows how the rebels the sympathizer supported had
    ‘The rebels had supported a sympathizer of the opposition, but nobody knows how the rebels had supported the sympathizer.’

Given that the antecedents in Paape’s study had either SVO or OVS word order, there was thus a mismatch between the feature set requested by the ellipsis gap, which would by assumption probe for an SOV feature, and the antecedent’s feature specification. This approach assumes that the word order of a clause is explicitly represented as a feature of the associated chunk in working memory. Even though no such assumption is made by the original Lewis & Vasishth model, the architecture is, in principle, able to represent and use word order like any other grammatical feature in order to find a suitable antecedent for an ellipsis. It is well known that ellipsis allows reference to partially mismatching antecedents, though there is some loss of acceptability (e.g. [7, 8]). Mismatching antecedents have been argued to require ‘repair’ in order to fit with the local (morpho-)syntactic context [9]. However, Paape argues that the mismatch effect is equally well explained if the local context of the ellipsis determines the cues for retrieving a matching antecedent: an SVO antecedent arguably provides a better match for the SOV cue set by the ellipsis than an OVS antecedent, given that the order of subject and object matches the gap’s local requirement. Despite the additional assumption that surface word order is encoded in the antecedent’s memory chunk, this latter approach offers the advantage of not having to assume an additional repair mechanism.

One caveat is that OSV is a permitted, though dispreferred, word order in German subordinate clauses. As the linear order mismatch between OSV and OVS is the same as between SOV and SVO, assuming that the gap probes for any permitted word order would predict no disadvantage for OVS antecedents. In order to explain the data, one must thus subscribe to the assumption that only the canonical word order serves as a retrieval cue.

As Paape [1]’s conclusions were arrived at post-hoc, it is important to subject them to further empirical investigation. The critical interaction between antecedent ambiguity and word order was found only in a late spillover region and after analyzing multiple regions of interest, which leaves open the possibility that the result is a false positive (type I error) [18]. Moreover, the observed power of Paape [1]’s experiment—which is likely an overestimate of the actual power [19]—was at 45%, which adds the possibility of a false negative result (type II error) with regard to potential effects at the ellipsis site.

Below, we present two new studies which were designed to test the reactivation hypothesis. Instead of self-paced reading, both of our experiments used eye tracking during reading, which arguably provides a more naturalistic way of presenting the stimuli. Self-paced reading is known to frequently show spillover effects, suggesting that participants may choose to hold words in memory rather than integrate them immediately. Such a strategy may be related to the adoption of a fixed rhythm in pressing the space bar [20, 21], which could obscure processing patterns that may become visible when the stimulus presentation more closely resembles normal reading.

Importantly, the eye tracking paradigm also provides subjects with the opportunity to make regressions to earlier parts of the sentence. One concern in relation to the original result was that the speedup observed for ambiguous OVS antecedents could be due to parsing failure rather than easier retrieval: if subjects fail to resolve the garden path and thus do not create a well-formed antecedent memory chunk, they may subsequently fail to resolve the ellipsis, given that they have no chance to return to the antecedent clause and reread it (though making a ‘covert’ regression is a logical possibility). The original study yielded no direct evidence for such failures: while comprehension accuracy suffered in the garden-path condition, questions targeting the interpretation of the antecedent and questions targeting other parts of the stimulus sentence were affected to the same degree, suggesting that parsing failure may not have been responsible for the errors. In eye tracking, targeted regressions to the antecedent in ambiguous sentences would indicate that the unavailability of a memory target may indeed play a role in creating the observed speedup at the ellipsis site, and that readers are making a second attempt at reanalyzing the antecedent.

## Experiment 1

The design of our first study differs from Paape’s [1] original experiment in three respects:

I) Language: Our experiment was carried out in French rather than German. Concordant evidence from the two languages would strengthen the claim that reactivation through reanalysis is a language-general phenomenon.
II) Method: We used eye tracking instead of self-paced reading.
III) Type of syntactic ambiguity: In addition to sentences with non-canonical word order which closely resembled Paape’s original German stimuli, our design also incorporated sentences containing main clause/reduced relative and lexical ambiguities, as explained below. Different types of ambiguity were used in order to test whether reactivation-based facilitation would occur in less similar environments as well.

If the reactivation hypothesis is correct, the observed pattern of faster processing of garden-path antecedents at the ellipsis site should be reproducible even when these parameters are changed.

### Materials

Like German, French allows sluicing, as shown in (4).

(4)
  Je veux acheter un cadeau, mais je ne sais pas lequel.
    I want to buy a present but I NEG know NEG which (one)
    ‘I want to buy a present, but I don’t know which one.’

By investigating possible antecedent reactivation effects in French sluicing constructions, we are thus looking at the same syntactic phenomenon investigated by Paape [1] in a different linguistic environment.

Given that syntactic reanalysis is critical to the reactivation hypothesis, the first challenge was to identify suitable French garden-path sentences, which are comparatively understudied. The few studies we could find investigated relative clause attachment ambiguities [22], ambiguous wh-questions with long-distance extraction [23] and a particular type of causative/non-causative ambiguity which is limited to the verb *faire*, ‘do’ [24]. None of these seemed suitable for our needs in the current study. Specifically, relative clause attachment has been argued to possibly involve underspecification [25], the grammatical status of long-distance extractions is dubious with some verb types [26], and the ambiguity of *faire* was deemed too lexically specific.

We identified three potential garden-path constructions in French which to our knowledge have not been investigated systematically before: sentences featuring subject-object inversion (SOI stimuli), sentences containing reduced relative clauses (RRC stimuli) and sentences containing a sequence of three lexically ambiguous words (triple lexical ambiguity, TLA stimuli). Apart from testing the reactivation hypothesis, the current study had the additional aim of establishing whether garden-path effects do indeed occur with these constructions. Furthermore, by using a set different stimulus types we hoped to curtail participants’ potential attunement to any one kind of garden-path structure (see [27] for a study demonstrating within-experiment attunement of syntactic processing). Lastly, if reactivation-based facilitation is found to occur with different ambiguity types, this would be a strong argument for the generalizability of the effect. Each of the three sentence types is discussed separately below.

The 56 experimental stimuli (20 × SOI + 16 × RRC + 20 × TLA) were combined with 24 filler sentences from an unrelated experiment, yielding a total of 80 sentences. Fewer RRC items were used because there is only a limited number of verbs in French whose third-person singular present form is identical to the participle, which constrained the number of possible stimulus sentences. Items were rotated through the experimental conditions according to a latin-square design, so that no subject saw the same sentence twice.

#### Type 1: Subject-object inversion (SOI)

Stimuli of the first type were wh-questions involving simple main clauses in which the order of subject and object was either canonical (SVO) or reversed (OVS), as shown in (5). OVS sequences are commonly analyzed as instances of so-called *stylistic inversion* [28, 29]. Diamonds indicate region-of-interest boundaries in all examples. The point of disambiguation and the critical ellipsis regions are marked by subscripts.

(5)
  Quel escrimeur ⋄ de l’équipe nationale ⋄ a / ont salué_disambig._ ⋄ les adversaires ⋄ du concours ⋄ et quand / avant la lutte,_critical_ ⋄ si je puis me permettre?
    Which fencer of the team national has / have greeted the opponents of.the contest and when / before the fight if I can _self_ allow
    ‘Which fencer of the national team greeted the opponents in the contest / did the opponents in the contest greet and when / before the fight, if I may ask?’

This sentence type almost directly parallels the German construction used by Paape [1]. The first noun phrase (*Quel escrimeur …*, ‘which fencer …’) could initially be either a subject or an object, though the subject reading is more common, and thus likely to be preferred by French readers, as it is by readers of other languages [30, 31]. Indeed, a search in the Frantext corpus (about 300 m words; [32]) for sentences starting with a wh-word revealed that 26 out of 29 questions involving both an animate subject and an animate object were subject-initial. As in German [33], the object-initial configuration in French has been argued to be a derived word order created either by shifting the subject NP to the right [29] or movement of the verb to the left [34]. In both cases, derivational economy would dictate that the SVO order be preferred (cf. also the Minimal Chain Principle of [35]).

One should note that claiming SVO to be *the* canonical word order in French is clearly problematic when sentences with clitics are considered [36], but significantly less so when all argument roles are filled by lexical noun phrases, as in the present study.

In (5), when the auxiliary bears singular marking (*a*, ‘has’), it agrees with the initial noun phrase, which is thus identified as the clause’s subject. When it is plural (*ont*, ‘have’) it agrees with the plural-marked second noun phrase (*les adversaires …*, ‘the opponents …’), which indicates that the initial noun phrase must be the direct object of *salué*, ‘greeted’.

The initial clause is coordinated with a sluice (*quand*, ‘when’) via the conjunction *et*, ‘and’, in the ellipsis conditions. In the control conditions, it continues with an adverbial phrase (*avant la lutte*, ‘before the fight’). Items of this type thus followed a 2 × 2 design with the factors canonicity (canonical vs. non-canonical) and elision (ellipsis vs. control). Each item ended with a set phrase (e.g. ‘… if I may ask’) which was intended as a spillover region.

A total of 20 SOI sentences were used in the experiment. In half of these the first noun phrase was singular and the second noun phrase was plural, as in (5), while for the other half the number features were switched, making plural marking on the auxiliary the form that agreed with the initial subject.

#### Type 2: Reduced relative clauses (RRC)

For the second stimulus type, we made use of the fact that some French verbs show syncretism between their third-person active and participle forms, such as *détruit*, ‘destroyed’, in (6). As in comparable English sentences, whose processing has been studied quite extensively (e.g. [37, 38]), this leads to a temporary ambiguity between an active reading of the initial clause (*The boat destroyed [something]*) and a reading in which *détruit* heads a reduced relative clause (RRC) modifying the initial noun. The sentence is disambiguated towards the latter reading at *avait rejoint*, ‘had returned (to)’, which marks the end of the relative clause. In French, an adverb can intervene between an active verb and its direct object, so the prepositional phrase *pendant la guerre*, ‘during the war’, does not disambiguate the sentence.

(6)
  **Ambiguous antecedent**
    Le navire ⋄ détruit ⋄ pendant la guerre ⋄ avait rejoint_disamb._ ⋄ le port,
    The ship destroyed during the war had returned.to the harbor
  **Unambiguous antecedent**
     Les navires ⋄ détruits ⋄ pendant la guerre ⋄ avaient rejoint_disambig._ ⋄ le port,
    The.pl ships destroyed.pl during the war had.pl returned.to the harbor
    ‘The ship(s) (that was/were) destroyed during the war had returned to the harbor, …’
  **Ellipsis**
    … mais le professeur ⋄ d’histoire ⋄ ne pouvait pas dire ⋄ quand,_critical_
    but the professor of history neg could neg say when
  **Control**
    … mais le professeur ⋄ d’histoire ⋄ n’en savait rien, _critical_
     but the professor of history neg.of it knew nothing
    ‘… but the history professor could not say when / knew nothing about it …’ … laissant ⋄ un peu désillusionnée ⋄ la jeune collègue ⋄ durant leur rendez-vous.
     leaving a bit disappointed the young colleague during their date
    ‘… leaving the young colleague a bit disappointed during their date.’

The unambiguous version of the sentence is obtained by explicitly marking the participle status of the verb form through number or gender agreement with the initial noun phrase. Note that while an active verb in this position would also agree with initial noun phrase, a different morphological paradigm would apply, and the corresponding form in (6) would be *détruisent*. Besides the ambiguity factor (ambiguous vs. unambiguous), RRC stimuli also featured an elision manipulation (ellipsis vs. control), again resulting in a 2 × 2 design.

In the ellipsis conditions, the clause headed by *mais*, ‘but’, ends with a sluice (… *quand*, ‘when’). The control conditions for this sentence type always contained a non-elliptical anaphor, such as *en*, ‘of.it’, in the above example. Across items, the anaphor sometimes appeared in the same region as the ellipsis, but could also appear at an earlier point (see item list in S1 File). The rest of the sentence served as a spillover region, and to keep subjects interested by providing a more elaborate discourse. A total of 16 RRC items were used in the experiment.

#### Type 3: Triple lexical ambiguity (TLA)

Items of the third stimulus type contained sequences of three lexically ambiguous words. In example (7), the translation of the first clause is ‘The filthy butcher cuts them’. The pronoun *les*, ‘them’, refers to the big sides of beef mentioned in the context sentence which accompanied the item.

(7)
  **Context**
    Le marché du quartier est connu pour les grandes côtes de bœuf qui sont livrées durant la nuit.
    The market of the district is known for the big sides of beef which are delivered during the night
  **Ambiguous antecedent**
    Le boucher ⋄ sale ⋄ les tranche,_disambig._
    the butcher filthy them cuts
  **Unambiguous antecedent**
    Les bouchers ⋄ sales ⋄ les tranchent,_disambig._
    the.pl butchers filthy.pl them cut
    ‘The filthy butcher(s) cut(s) them …’
  **Ellipsis**
    … mais les clients ⋄ de la place Colbert ⋄ se demandent ⋄ incrédulement ⋄ quand,_critical_
     but the clients of the square Colbert self ask disbelievingly when
  **Control**
    … mais les clients ⋄ de la place Colbert ⋄ en demandent ⋄ incrédulement ⋄ la technique,_*critical*_
     but the clients of the square Colbert of.it ask disbelievingly the technique
    ‘… but the clients of Colbert square ask disbelievingly when / about the technique of it, …’
    vu que la viande est vendue déjà marinée et prête à l’emploi en début de matinée.
    seen that the meat is sold already marinated and ready for the.use at beginning of morning
    ‘… given that the meat is sold already marinated and ready to use in the early morning.’

The words *sale*, *les* and *tranche* are all lexically ambiguous: *sale* is both the singular form of the adjective ‘filthy’ and the third-person singular form of the verb *saler*, ‘to salt’, while *les* can be either a plural-marked direct object pronoun or a plural-marked definite article. Besides being the third-person singular form of the verb ‘to cut’, *tranche* can also be a noun (‘slice’).

The alternative reading resulting from this chain of ambiguities, namely ‘The butcher salts the slices’, which is also compatible with the context sentence, is shown in (8). Note, however, that the would-be noun *tranche* would need to bear a plural suffix (-s) in order for this alternative reading to be grammatical. The stimulus sentence shown above in (7) is thus disambiguated towards the other reading by the absence of the plural marker.

(8)
  **Garden-path reading of (7)** Le boucher ⋄ sale ⋄ les tranches, …
    the butcher salts the slices

TLA stimuli also followed a 2 × 2 design with the factors ambiguity (ambiguous vs. unambiguous) and elision (ellipsis vs. control). The ambiguous reading becomes impossible when the initial noun phrase is plural and the adjective *sale*, ‘filthy’, bears a plural -s. Again, the third-person plural form of the verb *sale*, ‘to salt’, which is *salent*, would look different from the plural form of the adjective. The verb form *tranchent*, ‘cut’, also agrees with the plural feature of the initial noun phrase in the unambiguous conditions.

As for the RRC stimuli, the second clause, introduced by *mais*, ‘but’, contained either a sluice (… *quand*, ‘when’) or a pronoun (*en*, ‘of it’). Again, anaphor and ellipsis sometimes appeared within the same region for a given item, but this was not always the case. As before, the remainder of the sentence was intended both as a spillover region and as a reasonably interesting continuation of the discourse. The experiment contained 20 TLA sentences, each accompanied by a context sentence.

### Predictions

A vital assumption of the current design is that syntactic reanalysis of the antecedent clause is required across all three stimulus types. For SOI sentences, subjects are expected to initially compute an SVO structure, but the non-canonical versions force a switch to OVS at the auxiliary, much like in the German sentences of Paape [1]. For ambiguous TLA and RRC sentences, we assume that readers will initially adopt the preferred structure, which in both cases corresponds to an SVO active sentence, and are forced to abandon this analysis once the disambiguating word—either the singular-marked noun in TLA sentences or the finite verb in RRC sentences—arrives. We thus expect processing difficulty, in the form of elevated reading times that may be accompanied by regressions, at the disambiguating region across all stimulus types.

If the reactivation hypothesis is correct, reanalyzed antecedents should be easier to retrieve at the sluicing site in the elided conditions, leading to shorter reading times and possibly fewer and/or shorter regressions. In contrast, we do not expect to see an advantage for ambiguous/non-canonical antecedents in the control conditions, as pronouns are assumed to be resolved by discourse-based mechanisms instead of by the retrieval and integration of syntactic chunks from working memory [4].

### Pre-study: Off-line antecedent acceptability ratings

In order to assess whether garden-path effects were likely to be observed for our three stimulus types, we ran an Internet-based pre-study with 52 self-identified French native speakers who did not participate in the main study. The experiment was hosted on Ibex farm [39]. Informed consent was obtained from each participant prior to the experiment (in written form in the case of Experiments 1 and 2). The pre-study as well as the experiments described below complied with the June 1964 Declaration of Helsinki, as last revised. At the time of experimentation, both French and German law allowed non-invasive experiments with human subjects to be conducted without prior approval from an internal review board (or ethics committee). Instead, compliance with the Declaration of Helsinki was ensured by the principal investigator at the site of each experiment, in this case Prof. Barbara Hemforth for the pre-study and Experiment 1 and Prof. Shravan Vasishth for Experiment 2.

All stimulus sentences were truncated at the first comma, so that only the antecedent clause remained, which was presented at once in its entirety. There was thus only one manipulation for each sentence type: RRC and TLA stimuli were either ambiguous or unambiguous while SOI stimuli were either canonical or non-canonical. In the case of the TLA stimuli, the context sentence was visible on the screen, but participants were explicitly instructed to evaluate only the target sentence. Stimuli were presented in random order according to the design described above and participants were required to judge the acceptability of each sentence on a scale from 1 to 10. We opted for the 1-10 scale, as opposed to the more commonly used 1-7 scale, because French subjects are used to it from their time in school.

There was no time limit for giving the rating. We expected our participants to give lower ratings to sentences which cause processing difficulty, that is, to garden-path sentences as opposed to unambiguous controls.

#### Data analysis

The data were analyzed using the statistics software R [40] and the Stan programming language for Bayesian statistical inference [41]. As ratings on a Likert scale are discrete rather than continuous, and tend to show distributions that deviate from normality, we fitted a cumulative logit model across sentence types. Models of this type assume a latent variable *η* and a set of cutpoints *C* which demarcate the boundaries of each rating’s ‘bin’ in logit space. For instance, when *η* assumes a value between the cutpoints for rating 1 and rating 2, a rating of 1 will be assigned. One advantage of this approach is that the differences between any two adjacent cutpoints do not need to be the same, such that some manipulations may only affect ratings within a certain range on the Likert scale.

We assumed a common set of cutpoints across sentence types as well as across items and participants. In addition to a separate intercept on *η* and separate coefficients for the two experimental factors by sentence type, the model included random intercept and slope adjustments both by participants and by items. The ambiguity/canonicity factor was sum-coded, such that ambiguous/non-canonical sentences were coded as 1 and unambiguous/canonical sentences as −1. We set uninformative Cauchy(0, 2.5) priors for all coefficients and an LKJ prior [42] with parameter 2 for the variance-correlation matrices of the random effects for subjects and items.

Four chains with a total of 4000 iterations each and a warm-up period of 2000 iterations were run for each model. Convergence was verified based on Stan’s $\hat{R}$ statistic. We only report effects for which zero is not included in the associated 95% credible interval or for which there is nevertheless a high probability (> 95%) that the parameter is above or below zero given the data and the model.

#### Results

Mean ratings by sentence type and condition are shown in Table 1. There was no evidence of an effect of ambiguity on ratings for RRC sentences. For TLA sentences, ambiguous versions received lower ratings than unambiguous versions ($\hat{\beta }=-0.12$, CrI: [−0.23, −0.01], Pr(*β* > 0) = 0.02). For SOI sentences, non-canonical versions also received lower ratings than canonical versions ($\hat{\beta }=-1.02$, CrI: [−1.15, −0.89], Pr(*β* > 0) ≈ 0).

**Table 1:** Pre-study: Mean acceptability ratings and standard errors (in parentheses) by sentence type and condition.

**SOI stimuli**	canonical	non-canonical
	8.46 (0.10)	5.68 (0.15)
**RRC stimuli**	unambiguous	ambiguous
	7.94 (0.13)	7.83 (0.13)
**TLA stimuli**	unambiguous	ambiguous
	6.01 (0.14)	5.75 (0.14)

Given these preliminary results, we conclude that ambiguous TLA and non-canonical SOI stimuli caused greater processing difficulty for participants, leading to lower ratings, as intended. While there was no evidence of an effect for the RRC stimuli, this sentence type was carried over into the main experiment as well, given that a relatively mild garden path may still be detectable in an on-line experiment.

### Participants

We recruited 46 French native speakers between the ages of 19 and 38 through a mailing list. All had normal or corrected-to-normal eyesight. They were paid 7 for their participation. Informed consent was obtained from each participant prior to the experiment.

### Procedure

Participants were tested individually in a dimly lit, soundproof chamber. Subjects read the experimental sentences and fillers at their own pace while an SR Research Eyelink II tracker with a head-mounted camera setup recorded the movements of their dominant eye at a sampling rate of 500 Hz. Participants were seated at a distance of 75 cm from the presentation screen.

At the beginning of each experimental session the eye-tracker was calibrated using a nine-point grid. Each trial started with the presentation of a dot on the monitor which participants had to fixate in order to start the presentation of the stimulus sentence, whose first character then appeared in the same location as the dot. Participants signaled completion of a trial by pressing a button on a provided game pad.

Sentences were presented in 16 pt Times New Roman font at a resolution of 1024 × 768 pixels. At a screen width of 47.5 cm, one character accounted for 0.57° of visual angle. Line breaks were coded manually for longer sentences in order to avoid lines breaking directly before or after a region of interest. A chin rest was used to keep participants’ head position stable during tracking. Each session began with the presentation of two practice items to familiarize subjects with the procedure. The presentation order of the remaining sentences was randomized at runtime. All experimental items, but none of the filler items were followed by a yes/no comprehension probe. The probes targeted various parts of the sentence, including but not limited to the interpretation of the antecedent and the ellipsis. There were three obligatory breaks during the experiment, and subjects were told that they could take additional breaks at any time. The eye-tracker was recalibrated using a nine-point grid after each break. Recording sessions lasted 45 minutes on average.

### Data analysis

Due to repeated tracking loss and a computer crash, no analyzable data were collected for five participants. For the remaining 41 participants, data points in which a region had a total reading time of less than 20 ms were removed prior to the statistical analysis. Visual inspection suggested non-normality of residuals across all reported eye tracking measures. We thus applied the Box-Cox procedure [43] to identify an appropriate transform. The procedure suggested a logarithmic transformation of the response, hence all further analyses were carried out assuming that the dependent measures were log-normally distributed.

Our Stan model included a global standard deviation *σ* and separate intercepts *α*_*sr*_ per region of interest *r* of sentence type *s*, as shown in Eq (1) for first-pass reading times.

$$\begin{array}{cc} \mu_{srij}=\alpha_{sr}+u_{0i}+w_{sj}+u_{0sri}+w_{0srj}+ & \\ (\beta_{1}+u_{1i})\cdot length+ & \\ (\beta_{2sr}+u_{2sri}+w_{1srj})\cdot elision+ & \\ \ldots & \\ FPRT_{srij}∼Lognormal\left(\mu_{srij},\sigma \right) \end{array}$$ (1)

The intercept adjustment *u*_0*i*_ by subject *i* is global in the sense that it is applied to all regions of interest across all sentence types, thus taking into account that all items were read by the same subjects. The intercept adjustment *w*_0*sj*_ is applied across regions of interest for each item within each sentence type. Two further adjustments to the intercept, *u*_0*sri*_ and *w*_0*srj*_, are applied by region within each sentence type, for each item *j* of a given type and for each subject *i*. Together, these adjustments take into account possible variability in reading times between subjects and items for a particular region of interest for a particular sentence type. For all analyses of eye tracking measures, the log-transformed number of characters in each region of interest was added to the model as a centered covariate to control for length differences between conditions. The effect of length was estimated globally across items, regions and sentence types, but was allowed to vary between subjects by assuming the slope adjustment *u*_1*i*_. Lastly, slope adjustments *u*_2*sri*_ and *w*_1*srj*_ for each experimental factor account for variability in the effect of the experimental manipulations between items and subjects. Like the associated coefficient *β*, each adjustment is specific to region of interest *r* of sentence type *s*.

Analogous analyses, without the multiple regions of interest, were carried out for question response accuracies, which were analyzed using hierarchical logistic regression, as well as for log question response times. The accuracy model included a globally estimated coefficient with random slopes by subject for log response time to account for the interdependence between accuracy and response latency.

Across the three stimulus types, elision conditions were coded as 1 and control conditions as −1. For RRC and TLA stimuli, the ambiguous conditions were coded as 1 and the unambiguous conditions as −1. For SOI stimuli, non-canonical word order was coded as 1 and canonical word order as −1. Due to experimenter error, no data on question response accuracy and response time was recorded for the first three participants, thus the reported accuracy results refer to data from the 38 remaining participants. The eye-tracking data of all 41 participants were analyzed.

For nested comparisons as well as for the exploratory analyses, we used the rstanarm package [44], which interfaces R with Stan and simplifies model specification by emulating the syntax of the more commonly used lme4 package [45].

As before, we set Cauchy(0, 2.5) priors for all coefficients. An LKJ prior with parameter 2 was used for the variance-correlation matrices of the random effects for subjects and items. Four chains with 4000 iterations each were run for each model.

For easier interpretation, we have back-transformed the estimates in the text, along with their credible intervals, to the millisecond and probability scales, respectively. For comparison, model summaries are given on the log and logit scales, respectively.

### Results—Subject-object inversion (SOI)

**Fig 2:**
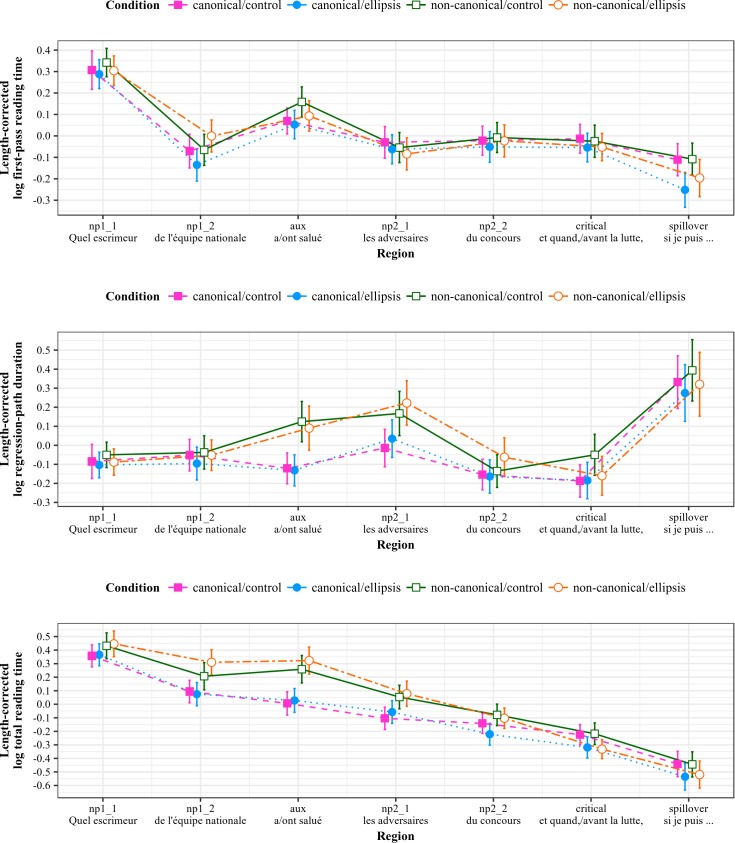
Reading measures by region for SOI stimuli (Experiment 1). All measures log-transformed and residualized against region length in characters; error bars show 95% intervals.

Length-corrected reading time measures by region of interest for the SOI stimuli are shown in Fig 2.

#### Comprehension probes

Table 2 shows the results for comprehension accuracy and response latency. The mean response accuracy for SOI stimuli was 74%. There were no effects of the experimental manipulations on response times. There was, however, an effect of the canonicity manipulation on response accuracy, such that less accurate responses were given after non-canonical sentences compared to canonical sentences ($\hat{\beta }=-25\%$, CrI: [−41%, −11%], Pr(*β* > 0) ≈ 0). There was also an effect of elision on response accuracy, such that more accurate responses were given after elided sentences ($\hat{\beta }=8\%$, CrI: [0%, 17%], Pr(*β* > 0) = 0.98).

**Table 2:** Experiment 1: Results for question response accuracy and response times (SOI stimuli). can = canonicity, el = elision.

response accuracy (logit scale)				
parameter	estimate	CrI low	CrI high	Pr(*β* > 0)
el	0.32	0.01	0.64	0.98
can	−0.95	−1.53	−0.42	≈0.00
el × can	−0.12	−0.46	0.22	0.25
response time (log scale)				
parameter	estimate	CrI low	CrI high	Pr(*β* > 0)
el	0.00	−0.05	0.04	0.45
can	0.03	−0.03	0.09	0.82
el × can	−0.02	−0.07	0.03	0.19

#### First-pass reading times

Results for first-pass reading times are shown in Table 3. There was an unexpected interaction between elision and canonicity in the second region of the first noun phrase ($\hat{\beta }$ = 41 ms, CrI: [−5 ms, 85 ms], Pr(*β* > 0 = 0.96). Given that elided and unelided sentences did not differ at this point and there was likely no parafoveal preview of the critical region, we have no ready explanation for this effect and speculate that it may be spurious. At the auxiliary, first-pass reading times were increased for non-canonical compared to canonical sentences ($\hat{\beta }$ = 21 ms, CrI: [−3 ms, 46 ms], Pr(*β* > 0 = 0.96). First-pass reading times were shorter for the elided than for the non-elided conditions in the spillover region following the critical region ($\hat{\beta }$ = −33 ms, CrI: [−64 ms, −2 ms], Pr(*β* > 0 = 0.02).

**Table 3:** Experiment 1: Results for first-pass reading times (SOI stimuli). can = canonicity, el = elision.

region	parameter	estimate	CrI low	CrI high	Pr(*β* > 0)
np1_1 *Quel escrimeur*	el	−0.01	−0.06	0.03	0.27
	can	0.01	−0.03	0.05	0.71
	el × can	−0.01	−0.05	0.04	0.38
np1_2 *de l’équipe nationale*	el	0.00	−0.05	0.04	0.45
	can	0.03	−0.01	0.08	0.92
	el × can	0.03	0.00	0.07	0.96
aux *a/ont salué*	el	−0.02	−0.06	0.02	0.17
	can	0.03	−0.01	0.07	0.96
	el × can	−0.01	−0.05	0.03	0.26
np2_1 *les adversaires*	el	−0.02	−0.07	0.04	0.28
	can	−0.01	−0.05	0.02	0.25
	el × can	0.00	−0.04	0.04	0.48
np2_2 *du concours*	el	−0.01	−0.05	0.03	0.28
	can	0.01	−0.04	0.06	0.65
	el × can	0.00	−0.04	0.04	0.45
crit *et quand,/avant la lutte,*	el	−0.02	−0.08	0.04	0.21
	can	0.00	−0.05	0.04	0.42
	el × can	0.01	−0.03	0.04	0.61
spillover *si je puis …*	el	−0.05	−0.10	0.00	0.02
	can	0.01	−0.04	0.06	0.65
	el × can	0.01	−0.03	0.06	0.70

#### Regression-path durations

Table 4 lists the results for regression-path durations by region. The region containing the disambiguating auxiliary and the participle showed an effect of canonicity, such that non-canonical sentences showed longer regression-path durations ($\hat{\beta }$ = 95 ms, CrI: [49 ms, 140 ms], Pr(*β* > 0) ≈ 1). This effect also appeared in the first region of the second noun phrase ($\hat{\beta }$ = 90 ms, CrI: [26 ms, 155 ms], Pr(*β* > 0) ≈ 1).

**Table 4:** Experiment 1: Results for regression-path durations (SOI stimuli). can = canonicity, el = elision.

region	parameter	estimate	CrI low	CrI high	Pr(*β* > 0)
np1_1 *Quel escrimeur*	See first-pass reading times				
np1_2 *de l’équipe nationale*	el	−0.02	−0.06	0.03	0.25
	can	0.02	−0.03	0.07	0.77
	el × can	0.01	−0.04	0.06	0.63
aux *a/ont salué*	el	−0.01	−0.06	0.04	0.38
	can	0.11	0.06	0.17	≈1.00
	el × can	−0.01	−0.06	0.04	0.36
np2_1 *les adversaires*	el	0.03	−0.04	0.10	0.78
	can	0.09	0.03	0.16	≈1.00
	el × can	0.00	−0.05	0.05	0.52
np2_2 *du concours*	el	0.01	−0.04	0.07	0.71
	can	0.03	−0.03	0.08	0.86
	el × can	0.02	−0.04	0.07	0.73
crit *et quand,/avant la lutte,*	el	−0.04	−0.12	0.05	0.20
	can	0.04	−0.03	0.11	0.87
	el × can	−0.02	−0.09	0.04	0.26
spillover *si je puis …*	el	−0.03	−0.13	0.06	0.23
	can	0.02	−0.06	0.10	0.70
	el × can	−0.01	−0.12	0.10	0.43

#### Total reading times

Table 5 shows the results for total reading times. Total reading times were elevated in non-canonical as compared to canonical sentences for the first ($\hat{\beta }$ = 81 ms, CrI: [−10 ms, 165 ms], Pr(*β* > 0) = 0.95) and second regions of the initial noun phrase ($\hat{\beta }$ = 133 ms, CrI: [60 ms, 202 ms], Pr(*β* > 0) ≈ 1). The effect was also present in the auxiliary region ($\hat{\beta }$ = 178 ms, CrI: [100 ms, 258 ms], Pr(*β* > 0) ≈ 1) as well as in the first ($\hat{\beta }$ = 84 ms, CrI: [35 ms, 133 ms], Pr(*β* > 0) ≈ 1) and second region of the second noun phrase ($\hat{\beta }$ = 45 ms, CrI: [2 ms, 90 ms], Pr(*β* > 0) = 0.98).

**Table 5:** Experiment 1: Results for total reading times (SOI stimuli). can = canonicity, el = elision.

region	parameter	estimate	CrI low	CrI high	Pr(*β* > 0)
np1_1 *Quel escrimeur*	el	0.01	−0.04	0.05	0.60
	can	0.04	0.00	0.08	0.96
	el × can	0.00	−0.04	0.04	0.51
np1_2 *de l’équipe nationale*	el	0.02	−0.02	0.06	0.83
	can	0.09	0.04	0.13	≈1.00
	el × can	0.03	−0.02	0.07	0.90
aux *a/ont salué*	el	0.02	−0.03	0.07	0.79
	can	0.14	0.08	0.20	≈1.00
	el × can	0.01	−0.04	0.05	0.61
np2_1 *les adversaires*	el	0.02	−0.03	0.06	0.80
	can	0.07	0.03	0.12	≈1.00
	el × can	−0.01	−0.05	0.04	0.40
np2_2 *du concours*	el	−0.03	−0.08	0.02	0.12
	can	0.04	0.00	0.09	0.98
	el × can	0.01	−0.03	0.05	0.69
crit *et quand,/avant la lutte,*	el	−0.04	−0.10	0.03	0.13
	can	0.00	−0.05	0.04	0.47
	el × can	0.00	−0.04	0.04	0.44
spillover *si je puis …*	el	−0.04	−0.09	0.02	0.08
	can	0.00	−0.05	0.06	0.51
	el × can	0.00	−0.06	0.07	0.54

### Results—Reduced relative clauses (RRC)

**Fig 3:**
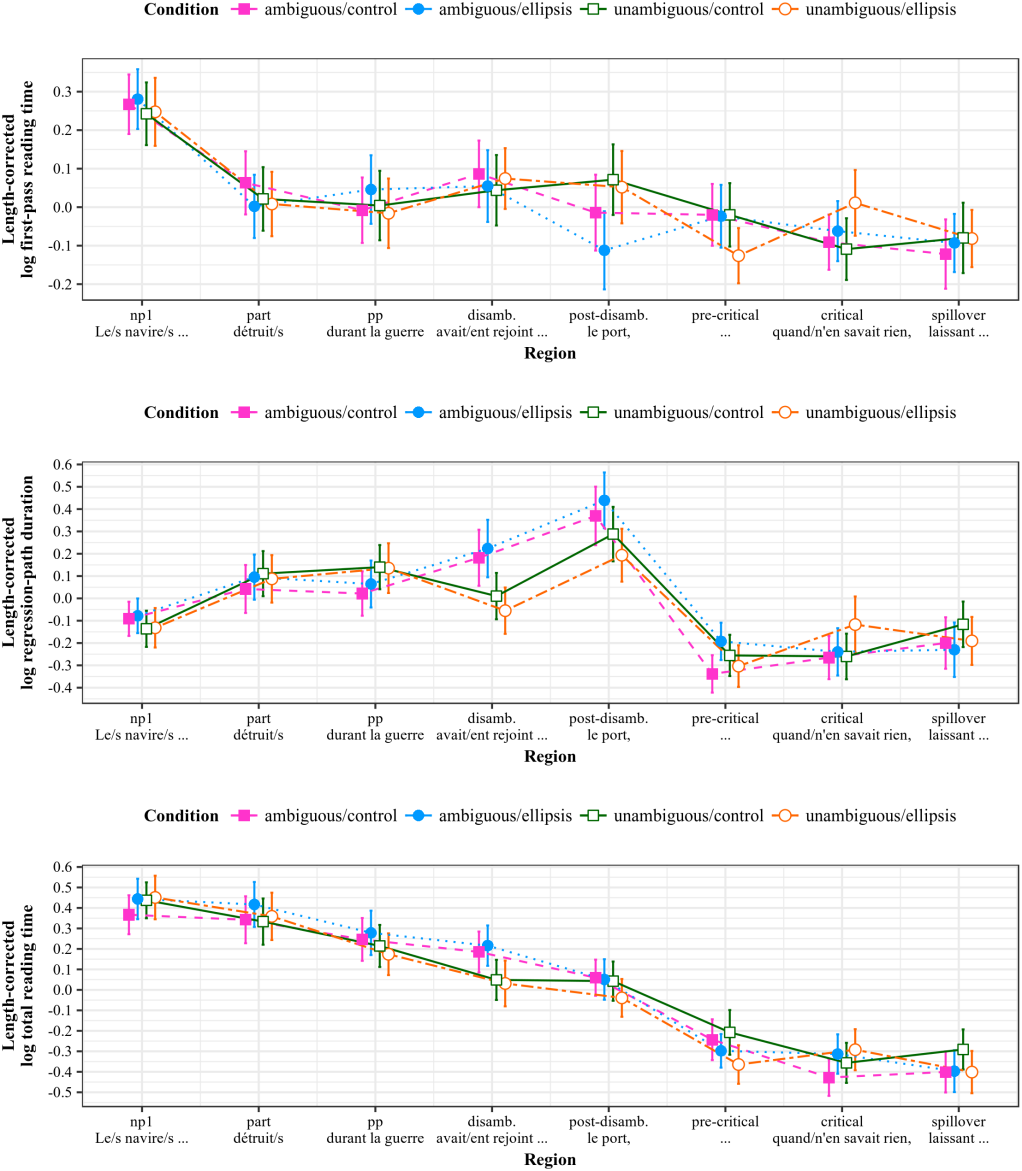
Reading measures by region for RRC stimuli (Experiment 1). All measures log-transformed and residualized against region length in characters; error bars show 95% intervals.

Length-corrected reading time measures by region of interest for the RRC stimuli are shown in Fig 3.

#### Comprehension probes

Results with regard to comprehension accuracy and response time are shown in Table 6. The mean probe response accuracy for the RRC stimuli was 86%. There were no effects of the experimental manipulations on response times. Response accuracy showed an effect of elision, such that questions about elided sentences were answered more accurately ($\hat{\beta }=5\%$, CrI: [−1%, 11%], Pr(*β* > 0) = 0.95).

**Table 6:** Experiment 1: Results for question response accuracy and response times (RRC stimuli). amb = ambiguity, el = elision.

response accuracy (logit scale)				
parameter	estimate	CrI low	CrI high	Pr(*β* > 0)
el	0.29	−0.05	0.66	0.95
amb	0.08	−0.25	0.41	0.70
el × amb	−0.18	−0.56	0.19	0.17
response time (log scale)				
parameter	estimate	CrI low	CrI high	Pr(*β* > 0)
el	−0.03	−0.10	0.04	0.22
amb	0.01	−0.05	0.07	0.59
el × amb	0.03	−0.03	0.09	0.84

#### First-pass reading times

Table 7 lists the results for first-pass reading times. In the post-disambiguating region, there was an effect of ambiguity, such that ambiguous sentences were read faster than unambiguous ones ($\hat{\beta }$ = −48 ms, CrI: [−98 ms, 2 ms], Pr(*β* > 0) = 0.03). In the spillover region following the critical region, elided sentences showed longer first-pass reading times than control sentences ($\hat{\beta }$ = 23 ms, CrI: [−4 ms, 51 ms], Pr(*β* > 0) = 0.95).

**Table 7:** Experiment 1: Results for first-pass reading times (RRC stimuli). amb = ambiguity, el = elision.

region	parameter	estimate	CrI low	CrI high	Pr(*β* > 0)
np1 *Le/s navire/s*	el	0.00	−0.05	0.05	0.57
	amb	0.01	−0.04	0.07	0.69
	el × amb	0.00	−0.04	0.04	0.57
part *détruit/s*	el	−0.02	−0.06	0.03	0.21
	amb	0.01	−0.04	0.05	0.61
	el × amb	−0.02	−0.07	0.04	0.27
pp *durant la guerre*	el	0.01	−0.05	0.07	0.63
	amb	0.01	−0.06	0.07	0.61
	el × amb	0.02	−0.04	0.07	0.75
disamb *avait/ent rejoint*	el	0.00	−0.06	0.06	0.46
	amb	0.01	−0.05	0.06	0.58
	el × amb	−0.02	−0.06	0.03	0.23
post-disamb *le port*	el	−0.02	−0.07	0.03	0.16
	amb	−0.06	−0.13	0.00	0.03
	el × amb	−0.02	−0.08	0.04	0.27
pre-crit *…*	el	−0.03	−0.10	0.04	0.23
	amb	0.02	−0.02	0.07	0.85
	el × amb	0.02	−0.03	0.07	0.81
crit *quand,/n’en savait rien,*	el	0.00	−0.06	0.06	0.54
	amb	−0.02	−0.07	0.03	0.24
	el × amb	0.00	−0.04	0.05	0.57
spillover *laissant*	el	0.04	−0.01	0.09	0.95
	amb	−0.02	−0.06	0.03	0.22
	el × amb	−0.02	−0.07	0.02	0.15

#### Regression-path durations

Results for regression-path durations are shown in Table 8. At the disambiguating region, regression-path durations were longer in the ambiguous compared to the unambiguous conditions ($\hat{\beta }$ = 108 ms, CrI: [29 ms, 193 ms], Pr(*β* > 0) = 0.99). The effect continued into the post-disambiguating region ($\hat{\beta }$ = 125 ms, CrI: [−19 ms, 275 ms], Pr(*β* > 0) = 0.96). At the pre-critical region, there was an interaction between elision and ambiguity ($\hat{\beta }$ = 62 ms, CrI: [−13 ms, 140 ms], Pr(*β* > 0) = 0.95), such that ambiguity increased regression-path durations in the elision conditions while the opposite was observed in the control conditions, with neither effect driving the interaction.

**Table 8:** Experiment 1: Results for regression-path durations (RRC stimuli). amb = ambiguity, el = elision.

region	parameter	estimate	CrI low	CrI high	Pr(*β* > 0)
np1 *Le/s navire/s*	See first-pass reading times				
part *détruit/s*	el	0.00	−0.06	0.06	0.56
	amb	−0.02	−0.11	0.07	0.31
	el × amb	0.01	−0.04	0.07	0.67
pp *durant la guerre*	el	0.01	−0.05	0.07	0.63
	amb	−0.05	−0.13	0.03	0.11
	el × amb	0.01	−0.05	0.07	0.65
disamb *avait/ent rejoint*	el	−0.01	−0.08	0.06	0.40
	amb	0.11	0.03	0.20	0.99
	el × amb	0.02	−0.05	0.09	0.69
post-disamb *le port*	el	−0.01	−0.08	0.07	0.44
	amb	0.08	−0.01	0.17	0.96
	el × amb	0.04	−0.02	0.10	0.88
pre-crit *…*	el	0.02	−0.05	0.09	0.73
	amb	0.01	−0.05	0.07	0.61
	el × amb	0.04	−0.01	0.09	0.95
crit *quand,/n’en savait rien,*	el	−0.04	−0.11	0.03	0.11
	amb	−0.03	−0.10	0.03	0.17
	el × amb	0.01	−0.05	0.07	0.58
spillover *laissant*	el	0.04	−0.03	0.11	0.88
	amb	−0.03	−0.09	0.03	0.13
	el × amb	−0.03	−0.09	0.03	0.17

#### Total reading times

Total reading times at the disambiguating region were higher in the ambiguous than in the unambiguous conditions ($\hat{\beta }$ = 112 ms, CrI: [32 ms, 195 ms], Pr(*β* > 0) ≈ 1). Table 9 shows results across all regions of interest.

**Table 9:** Experiment 1: Results for total reading times (RRC stimuli). amb = ambiguity, el = elision.

region	parameter	estimate	CrI low	CrI high	Pr(*β* > 0)
np1 *Le/s navire/s*	el	0.02	−0.03	0.07	0.81
	amb	−0.01	−0.06	0.04	0.32
	el × amb	0.01	−0.03	0.06	0.72
part *détruit/s*	el	0.02	−0.03	0.08	0.83
	amb	0.03	−0.03	0.08	0.83
	el × amb	0.01	−0.04	0.06	0.66
pp *durant la guerre*	el	0.00	−0.05	0.05	0.51
	amb	0.03	−0.01	0.08	0.94
	el × amb	0.01	−0.03	0.06	0.73
disamb *avait/ent rejoint*	el	0.00	−0.05	0.05	0.55
	amb	0.09	0.02	0.15	≈1.00
	el × amb	0.01	−0.06	0.08	0.62
post-diamb *le port*	el	−0.02	−0.07	0.03	0.21
	amb	0.02	−0.04	0.09	0.80
	el × amb	0.02	−0.03	0.07	0.76
pre-crit *…*	el	−0.05	−0.13	0.02	0.08
	amb	0.00	−0.04	0.05	0.57
	el × amb	0.02	−0.02	0.07	0.84
crit *quand,/n’en savait rien,*	el	0.01	−0.09	0.12	0.61
	amb	−0.03	−0.09	0.02	0.14
	el × amb	0.03	−0.02	0.08	0.85
spillover *laissant*	el	0.04	−0.03	0.10	0.89
	amb	−0.03	−0.07	0.02	0.12
	el × amb	0.00	−0.04	0.05	0.57

### Results—Triple lexical ambiguity (TLA)

**Fig 4:**
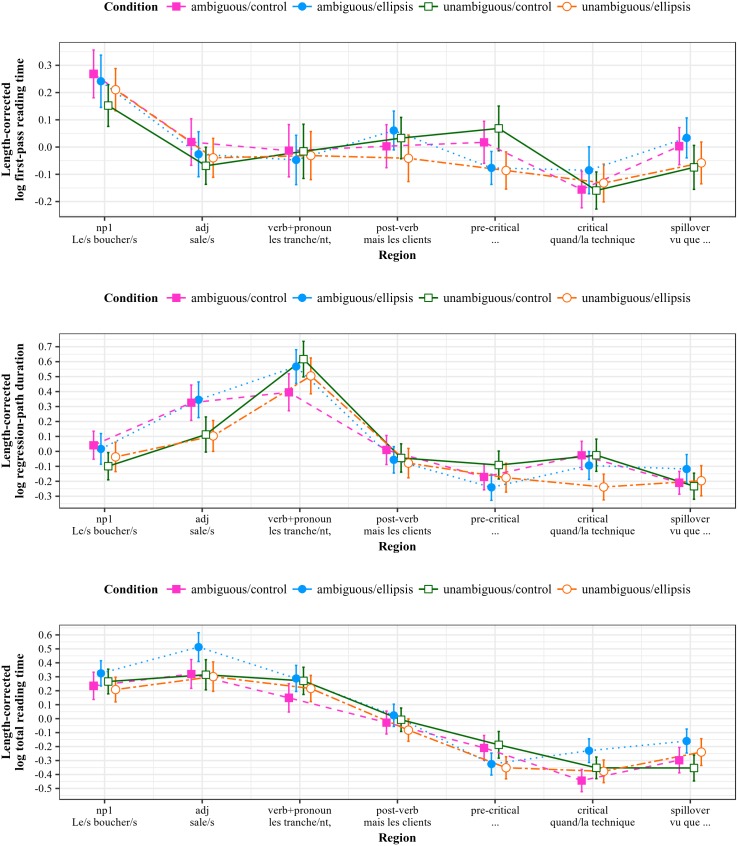
Reading measures by region for TLA stimuli (Experiment 1). All measures log-transformed and residualized against region length in characters; error bars show 95% intervals.

Length-corrected reading time measures by region of interest for the TLA stimuli are shown in Fig 4.

#### Comprehension probes

Results for question response accuracy and response latency are shown in Table 10. The mean probe response accuracy for TLA stimuli was 80%. There were no effects of the experimental manipulations on response accuracy or response times.

**Table 10:** Experiment 1: Results for question response accuracy and response times (TLA stimuli). amb = ambiguity, el = elision.

response accuracy (logit scale)				
parameter	estimate	CrI low	CrI high	Pr(*β* > 0)
el	−0.13	−0.56	0.29	0.28
amb	−0.07	−0.34	0.19	0.30
el × amb	0.00	−0.39	0.38	0.52
response time (log scale)				
parameter	estimate	CrI low	CrI high	Pr(*β* > 0)
el	0.00	−0.04	0.04	0.53
amb	0.00	−0.04	0.05	0.57
el × amb	0.01	−0.04	0.05	0.64

#### First-pass reading times

Table 11 shows the results for first-pass reading times. At the initial noun phrase, first-pass reading times were elevated in the ambiguous compared to the unambiguous conditions ($\hat{\beta }$ = 30 ms, CrI: [−4 ms, 65 ms], Pr(*β* > 0) = 0.96). At the post-verbal region, there was an unexpected interaction ($\hat{\beta }$ = 58 ms, CrI: [−8 ms, 122 ms], Pr(*β* > 0) = 0.96). We have no explanation for this effect as elided and control sentences did not differ at this point of the sentence. Speculatively, however, participants may have had preview of the critical region on the following line of text, even though this preview would have needed to cross two empty lines.

**Table 11:** Experiment 1: Results for first-pass reading times (TLA stimuli). amb = ambiguity, el = elision.

region	parameter	estimate	CrI low	CrI high	Pr(*β* > 0)
np1 *Le/s boucher/s*	el	0.01	−0.04	0.05	0.62
	amb	0.04	0.00	0.08	0.96
	el × amb	−0.02	−0.06	0.02	0.17
adj *sale/s*	el	0.00	−0.05	0.04	0.47
	amb	0.02	−0.02	0.06	0.82
	el × amb	−0.02	−0.07	0.02	0.14
verb+pronoun *les tranche/nt,*	el	−0.01	−0.06	0.03	0.26
	amb	−0.01	−0.06	0.05	0.38
	el × amb	0.00	−0.08	0.06	0.45
post-verb *mais les clients*	el	0.00	−0.04	0.04	0.45
	amb	0.02	−0.02	0.05	0.80
	el × amb	0.04	0.00	0.08	0.96
pre-crit *…*	el	−0.06	−0.12	−0.01	0.01
	amb	−0.01	−0.05	0.03	0.24
	el × amb	0.01	−0.03	0.05	0.70
crit *quand,/la technique,*	el	0.03	−0.03	0.09	0.88
	amb	0.01	−0.03	0.05	0.62
	el × amb	0.01	−0.03	0.05	0.72
spillover *vu que …*	el	0.01	−0.02	0.05	0.79
	amb	0.04	0.00	0.08	0.97
	el × amb	0.00	−0.04	0.04	0.45

At the pre-critical region, first-pass reading times were shorter for ellipsis than for control sentences ($\hat{\beta }$ = −38 ms, CrI: [−70 ms, −5 ms], Pr(*β* > 0) = 0.01). In the spillover region following the critical region, there was an effect of ambiguity on first-pass reading times, such that reading times were elevated in the ambiguous conditions ($\hat{\beta }$ = 23 ms, CrI: [−1 ms, 46 ms], Pr(*β* > 0) = 0.97).

#### Regression-path durations

Regression-path durations were elevated for ambiguous compared to unambiguous sentences on the first noun phrase ($\hat{\beta }$ = 40 ms, CrI: [−8 ms, 87 ms], Pr(*β* > 0) = 0.95), indicating longer regressions towards the context sentence. Longer regression-path durations were also observed for ambiguous sentences at the adjective ($\hat{\beta }$ = 87 ms, CrI: [30 ms, 146 ms], Pr(*β* > 0) ≈ 1). Next, there was a surprising interaction between ambiguity and elision in regression-path durations on the verb+pronoun region ($\hat{\beta }$ = 225 ms, CrI: [25 ms, 425 ms], Pr(*β* > 0) = 0.99). Elision and control sentences did not differ at this point, and planned comparisons revealed the interaction to be driven by neither sentence type. We speculate that the effect is either spurious or due to unintended parafoveal preview of the next line of text.

At the pre-critical region, ambiguous sentences had shorter regression paths than unambiguous sentences ($\hat{\beta }$ = −31 ms, CrI: [−69 ms, 7 ms], Pr(*β* > 0) = 0.05). At the critical region, regression-path durations were shorter for ellipsis than for control sentences ($\hat{\beta }$ = −54 ms, CrI: [−108 ms, −2 ms], Pr(*β* > 0) = 0.02). Table 12 lists the results for all regions of interest.

**Table 12:** Experiment 1: Results for regression-path durations (TLA stimuli). amb = ambiguity, el = elision.

region	parameter	estimate	CrI low	CrI high	Pr(*β* > 0)
np1 *Le/s boucher/s*	el	0.01	−0.04	0.07	0.68
	amb	0.04	−0.01	0.10	0.95
	el × amb	−0.02	−0.07	0.03	0.20
adj *sale/s*	el	0.00	−0.05	0.06	0.56
	amb	0.11	0.04	0.18	≈1.00
	el × amb	0.00	−0.07	0.07	0.54
verb+pronoun *les tranche/nt,*	el	0.02	−0.05	0.08	0.69
	amb	−0.05	−0.15	0.06	0.20
	el × amb	0.07	0.01	0.12	0.99
post-verb *mais les clients*	el	−0.02	−0.07	0.03	0.18
	amb	0.02	−0.04	0.07	0.71
	el × amb	0.00	−0.06	0.05	0.43
pre-crit *…*	el	−0.05	−0.11	0.02	0.09
	amb	−0.04	−0.09	0.01	0.05
	el × amb	0.00	−0.05	0.05	0.53
crit *quand,/la technique,*	el	−0.06	−0.13	0.00	0.02
	amb	0.03	−0.03	0.08	0.84
	el × amb	0.03	−0.02	0.09	0.91
spillover *vu que …*	el	0.03	−0.01	0.09	0.92
	amb	0.02	−0.03	0.07	0.80
	el × amb	0.00	−0.05	0.06	0.58

#### Total reading times

Results for total reading times are listed in Table 13. The adjective showed an interaction between elision and ambiguity ($\hat{\beta }$ = 126 ms, CrI: [15 ms, 241 ms], Pr(*β* > 0) = 0.98), driven by longer reading times in ambiguous elided sentences ($\hat{\beta }$ = 89 ms, CrI: [−23 ms, 200 ms], Pr(*β* > 0) = 0.95). An interaction with the same sign was evident at the verb+pronoun region ($\hat{\beta }$ = 149 ms, CrI: [16 ms, 283 ms], Pr(*β* > 0) = 0.99), but was driven by neither pair of conditions.

**Table 13:** Experiment 1: Results for total reading times (TLA stimuli). amb = ambiguity, el = elision.

region	parameter	estimate	CrI low	CrI high	Pr(*β* > 0)
np1 *Le/s boucher/s*	el	0.01	−0.03	0.06	0.69
	amb	0.03	−0.02	0.08	0.89
	el × amb	0.03	−0.01	0.08	0.93
adj *sale/s*	el	0.05	−0.02	0.12	0.92
	amb	0.06	−0.02	0.14	0.94
	el × amb	0.05	0.00	0.09	0.98
verb+pronoun *les tranche/nt,*	el	0.02	−0.03	0.08	0.79
	amb	−0.01	−0.07	0.06	0.41
	el × amb	0.05	0.01	0.09	0.99
post-verb *mais les clients*	el	0.00	−0.06	0.05	0.44
	amb	0.02	−0.03	0.06	0.77
	el × amb	0.03	−0.02	0.08	0.91
pre-crit *…*	el	−0.06	−0.13	0.00	0.03
	amb	−0.01	−0.05	0.04	0.38
	el × amb	0.01	−0.03	0.06	0.71
crit *quand,/la technique,*	el	0.05	−0.01	0.11	0.96
	amb	0.01	−0.04	0.05	0.64
	el × amb	0.06	0.01	0.10	0.99
spillover *vu que …*	el	0.06	−0.01	0.13	0.96
	amb	0.03	−0.02	0.07	0.89
	el × amb	0.00	−0.04	0.05	0.59

At the pre-critical region, sentences with elision showed shorter total reading times than control sentences ($\hat{\beta }$ = −55 ms, CrI: [−111 ms, 1 ms], Pr(*β* > 0) = 0.03). At the critical region, elided sentences showed longer reading times than control sentences ($\hat{\beta }$ = 44 ms, CrI: [−6 ms, 93 ms], Pr(*β* > 0) = 0.96). There was also an interaction between elision and ambiguity ($\hat{\beta }$ = 96 ms, CrI: [19 ms, 172 ms], Pr(*β* > 0) = 0.99), mainly driven by longer reading times in ambiguous elided sentences ($\hat{\beta }$ = 58 ms, CrI: [4 ms, 112 ms], Pr(*β* > 0) = 0.98). At the spillover region following the critical region, elided sentences again showed longer total reading times than non-elided sentences ($\hat{\beta }$ = 52 ms, CrI: [−6 ms, 110 ms], Pr(*β* > 0) = 0.96).

### Discussion

#### Garden paths in French

Our design rested on the assumption that all three stimulus types would cause garden-path effects, which was largely borne out in the data (but see discussion below). Off-line acceptability judgments had already shown that the ambiguous and non-canonical versions of TLA and SOI stimuli, respectively, received lower ratings, which we took to indicate processing difficulty on part of our participants. RRC stimuli, for which no effect had been found in off-line acceptability judgments, nevertheless showed an effect of ambiguity in on-line measures, specifically in both regression-path durations and total reading times at the disambiguating finite verb.

For SOI stimuli, on-line processing difficulty was also observed at the disambiguating region, again in the form of longer regression paths and higher total reading times. The garden-path effect for the OVS sentences appears to be quite strong, as evidenced by its persistence across the following regions and the effect on total reading times across the whole antecedent clause. It is also striking that SOI stimuli showed the lowest comprehension accuracy among the three item types. This, however, may be due to the fact that as SOI sentences contained less material between antecedent and ellipsis site than both RRC and TLA sentences, a larger amount of comprehension questions necessarily targeted the assignment of argument roles within the antecedent, so that misinterpretations could be more easily detected.

#### The reactivation hypothesis

The current study yielded no evidence in favor of the reactivation hypothesis, which predicted that an ellipsis should be easier to process in sentences where the antecedent has been syntactically reanalyzed. For SOI stimuli, we found no evidence that having experienced a garden path while processing the antecedent had any effect on the subsequent processing of the ellipsis. For TLA stimuli, we observed the opposite of what the reactivation hypothesis predicted: when the antecedent clause was temporarily ambiguous, ellipsis was more difficult to process. For RRC stimuli, there was evidence of increased processing difficulty in the pre-critical region when the antecedent was temporarily ambiguous and the following critical region contained an ellipsis; this effect, however, is somewhat difficult to interpret as it could stem either from parafoveal preview of the ellipsis site or from reading a pronoun in the pre-critical region in the control conditions, or both.

More generally, the multiple interactions we observed between the experimental factors in regions where the critical region had not been encountered yet are a reason for concern. Note, however, that our hypotheses did not concern any of these effects, but were limited to an expected garden-path effect at the disambiguating region of the antecedent, as well as effects at the critical ellipsis region. Still, while it is not unlikely that the unexpected effects are false positives, we cannot rule out the possibility that they are due to unintended preview that may also have affected later processing of the critical region.

The first interesting question to ask is why German readers showed a reactivation effect in Paape [1] while the French readers showed the opposite effect, namely difficulty processing the ellipsis in the presence of temporarily ambiguous antecedents in TLA stimuli, and possible RRC stimuli as well. A second question is why French readers showed a clear reverse effect only with TLA stimuli, but not in sentences with subject-object inversion or when the antecedent contained a reduced relative clause, despite evidence that there was garden-pathing in these latter stimuli. We believe that the difference in methods between Paape’s [1] experiment and the current study, the idiosyncrasies of the stimuli used as well as cross-linguistic differences in the acceptability of object-initial sentences may be jointly responsible for the diverging results. We discuss these points next.

While we chose to use eye tracking to make processing of the stimuli more natural for our participants, different styles and strategies may emerge in comparison to the self-paced reading methodology used in the original study. In non-cumulative self-paced reading, participants are aware that they cannot make regressions to earlier parts of the sentence, which could conceivably result in a more careful processing strategy. By ‘more careful’, we mean a strategy that minimizes strategic underspecification on part of the reader: a reader who knows that regressions are possible might be tempted to not fully disambiguate the syntactic structure of the antecedent, hoping that later information would point towards the correct analysis, or that choosing the correct analysis was simply not relevant to the task at hand. While evidence for underspecification has repeatedly been found in self-paced reading experiments [7, 25], its use may be still more prevalent when readers know that they can re-read earlier parts of the sentence at their leisure.

Especially with regard to our TLA stimuli, we suspect that at least on some trials, participants failed or neglected to resolve the ambiguity before proceeding past the antecedent clause. The first piece of evidence for this assumption is that TLA stimuli failed to show a classical garden-path effect that could be attributed to reanalysis, that is, an indication of processing difficulty for ambiguous sentences at or directly after the point of disambiguation. Instead, an ambiguity disadvantage was visible at the adjective, likely indicating that participants were torn between the two possible readings, that is, a competition effect. If participants failed to settle on a reading, it is not surprising that there was no evidence of revision at the point of disambiguation. Looking at first-pass reading times and regression-path durations, there was even a tendency for ambiguous sentences to be easier to process than unambiguous ones at this position. We assume that when participants reached the ellipsis site, they realized that they did not have a suitable antecedent available for retrieval, which led to elevated total reading times at this position (as well as regressions towards the antecedent, as shown in the next section). No reactivation advantage was observed because reanalysis sometimes did not take place prior to reaching the ellipsis site.

With regard to our SOI stimuli, we have some anecdotal evidence from three participants who claimed that the non-canonical versions were ungrammatical. While we have it on good authority that OVS word order in wh-questions is, in fact, grammatical in French [28], it is nevertheless worrying that some subjects would claim otherwise, which may indicate that they failed to parse the non-canonical stimuli. Note that this presents a stark contrast to German, where object-initial wh-questions are quite common, even though the object analysis is dispreferred in ambiguous cases [46]. If a large enough group of our French participants was under the impression of being presented with ungrammatical stimuli in the non-canonical conditions, it would not be surprising if interpretation of the ellipsis was largely suspended, given that the clause could not be assigned a meaning anyway.

**Fig 5:**
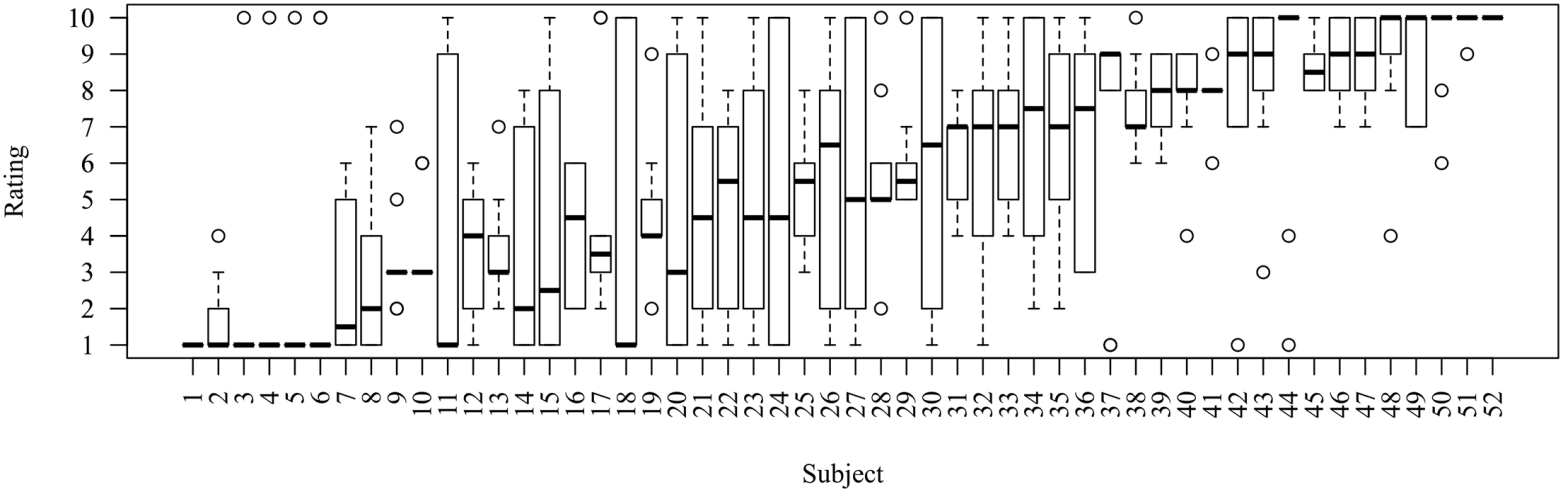
Rating distributions from the pilot study by participant.

In order to investigate the issue further, we took a closer look at the distributions of ratings given to inverted SOI stimuli in the pilot study, where the object-initial construction received a mean rating of about 5.7 out of 10. Prima facie, this value does not seem to indicate a general impression of ungrammaticality, given that the stimuli were expected to cause a garden-path effect and thus receive a comparatively low rating. Fig 5 shows the distribution of acceptability ratings for each participant.

As the plot indicates, there were six participants whose median rating was 1, meaning that at least half of the stimuli received a rating of 1, that is, were deemed completely unacceptable. Still, all but two of these participants also assigned perfect 10 ratings to a range of inverted SOI stimuli, suggesting that the construction was not seen as uniformly ungrammatical. Given these observations, we conclude that there may be a small minority of French speakers who indeed view subject-object inversion as ungrammatical, but that for the majority of speakers inversion is generally possible despite exhibiting a dispreferred ordering of arguments.

It is possible that the impression of ungrammaticality in the main experiment was partly due to an experimenter effect: as the main author, who carried out the experiment, is not a native speaker of French, participants may have been biased to expect some proportion of ungrammatical sentences. As a precaution, we ensured that a second experimenter who was a native speaker was always present during the experimental sessions, and explicitly informed participants that the stimuli had been designed by a native speaker. Still, some participants insisted on having read ungrammatical sentences even when the second experimenter explained the construction during debriefing.

There were no comments indicating that RRC stimuli were perceived as ungrammatical. Rather, it appears that they were the easiest to process, compared to the other stimulus types. We thus speculate that the reactivation effect for RRC stimuli was too subtle to be clearly detectable in our experiment, possibly because the construction causes garden-pathing less reliably in French, as suggested by the results of our pre-study, where RRC items failed to show an effect of the ambiguity manipulation on acceptability ratings.

### Design analysis

An issue of statistical power is implied when effects are believed to have been too small to be detectable in any given experiment. In a Bayesian setting, one is generally not interested in refuting the particular hypothesis that an effect size is zero, but rather in the amount of evidence for an effect being greater or smaller than zero, respectively, as well as in the precision of the estimate, that is, how wide the credible interval is [47]. Rather than it being most likely that there is no interaction between garden-pathing and elision in the SOI and RRC conditions, our results indicate that any effects could be either positive or negative, that is, could be speedups or slowdowns; the credible intervals put boundaries on the likely numerical size of the effect in each direction.

**Fig 6:**
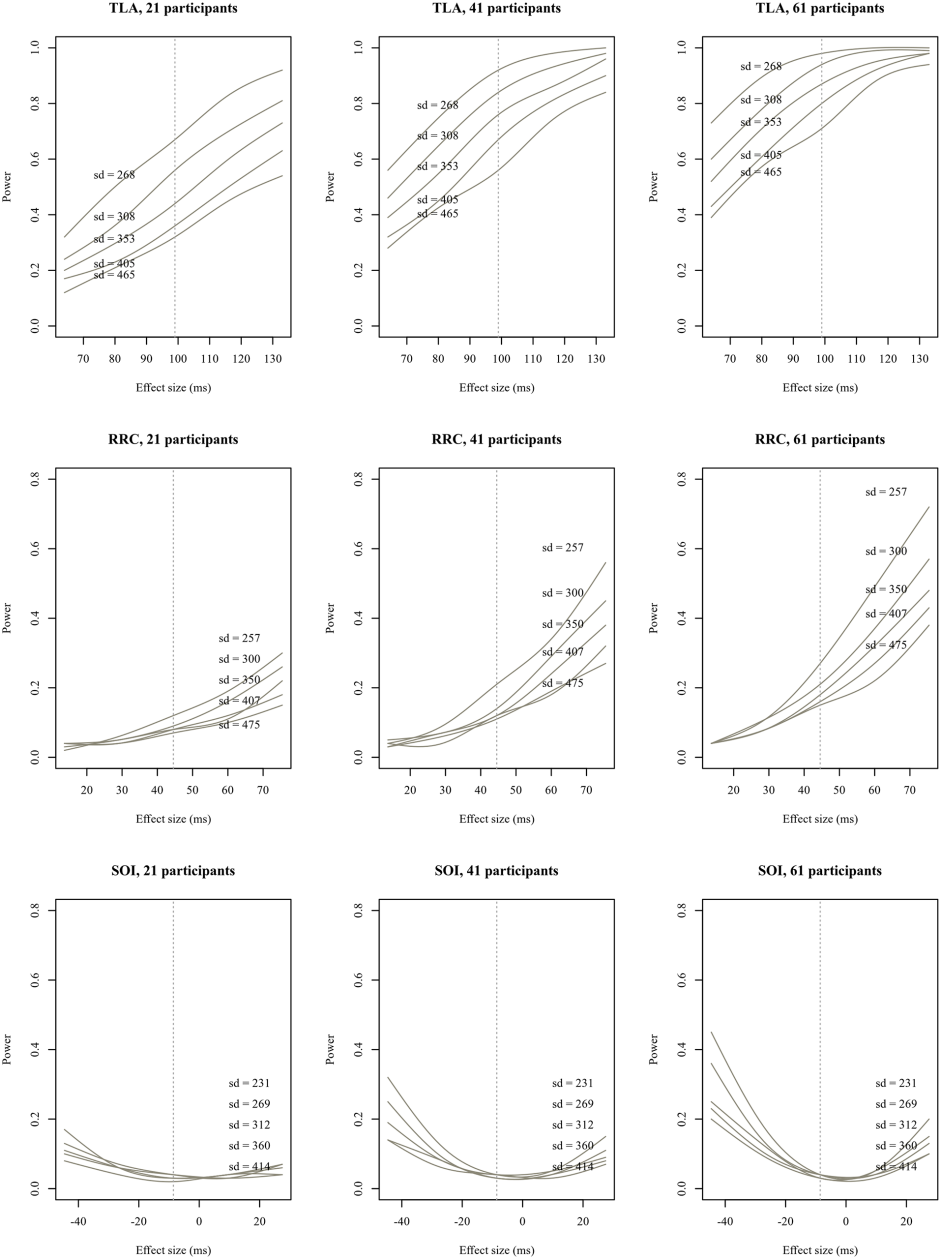
Estimated power (smoothed) as a function of interaction size, sample size and residual variance in total reading times at the critical region by stimulus type.

Statistical power (or precision) is not only a function of sample size, but also depends on the inherent variability of the dependent measure as well as the unknown ‘true’ effect size. In order to demonstrate changes in power dependent on effect size, sample size and residual variance, we ran a simulation-based design analysis by repeatedly generating data sets based on the model parameters computed from the original data. For our simulations, we varied the size of the ‘true’ interaction (now under our direct control, see [18] for a similar approach) in steps of 0.01 on the log scale, as well as the number of participants (our current study size plus or minus twenty participants), as well as the residual variance of the dependent measure, in this case total reading times. Within each sentence type, for each hypothetical effect size, sample size and variance, we generated 1000 data sets using the simulate function of the lme4 package [45] and fitted maximal frequentist models to total reading time at the critical ellipsis region. As Fig 6 shows, if the estimate for TLA sentences (around 96 ms) is reflective of the true effect size, and if the estimated residual variance is realistic, our power to detect it was about 70%. For the other sentence types, effects in the range of the estimate yielded by the relevant maximal model are unlikely to be detected (between 5% and 14% power), even under optimistic assumptions about variance.

While it should be kept in mind that the observed effect size for TLA stimuli, and therefore the associated power, is likely to be an overestimate [19], the relevant credible interval still gives a range of plausible effect sizes, and allows for the qualitative conclusion that the effect is likely to be positive (given the data and the model), whereas no such conclusion can be drawn for SOI and RRC sentences. However, what can be said is that the effects for both of the latter sentence types would have needed to be large (around 100 ms) in order for us to be able to reliably detect them, given the amount of variability present in the data.

### Exploratory analyses

#### Exploratory analysis I—Regressions and antecedent re-reading in TLA stimuli

For TLA stimuli, the analyses of total reading times for the verb+pronoun region showed effects of the elision manipulation, possibly indicating differential amounts of re-reading. However, based on this result alone, we cannot be sure when the re-reading occurred: a pronoun appearing before the critical region may trigger regressions, leading to higher re-reading times for the antecedent on a given trial even though the ellipsis has not been processed yet. To address this issue, we separated the data for TLA stimuli into fixations made before the critical region was first fixated on a given trial and fixations made after the first fixation on the critical region and computed reading measures separately using the em2 R package [48].

When only reading times that were generated before the first fixation on the critical region were considered, none of the three regions of the antecedent clause, that is, the initial noun phrase, the adjective and the verb+pronoun region, showed any effects of the experimental manipulations. The same was true when only reading times that were generated *after* the first fixation on the critical region were considered.

Looking at overall re-reading probabilities for the antecedent regions, there was an interaction at the first noun phrase ($\hat{\beta }=21\%$, CrI: [1%, 41%], Pr(*β* > 0) = 0.98) as well as at the adjective ($\hat{\beta }=19\%$, CrI: [1%, 38%], Pr(*β* > 0) = 0.97), the latter being driven by additional refixations in ambiguous sentences with elision ($\hat{\beta }=14\%$, CrI: [1%, 26%], Pr(*β* > 0) = 0.99).

When the re-reading times for all antecedent regions after the first fixation on the critical region were summed up, there was an effect of ambiguity ($\hat{\beta }$ = 240 ms, CrI: [−16 ms, 410 ms], Pr(*β* > 0) = 0.97), such that ambiguous sentences caused more re-reading.

#### Discussion

The results of the exploratory analysis indicate that the adjective region was refixated more often in elided sentences with ambiguous antecedents, a finding that matches the increased reading times at the critical region in this condition: when readers realized that they could not retrieve a suitable antecedent structure, they presumably returned to the antecedent in order to attempt a re-parse. Furthermore, the increase in antecedent re-reading times in the ambiguous conditions may indicate that both ellipsis and pronouns were affected by the occasional unavailability of an antecedent, which led to attempts to re-parse the problematic earlier part of the sentence. It should be kept in mind, however, that these are post-hoc interpretations of the patterns that should be addressed more systematically in future work.

#### Exploratory analysis II—Effects of lexical bias in TLA stimuli

There was no evidence for a classical garden path in TLA stimuli, as no effect of ambiguity was detected in the disambiguating region, even though ambiguous sentences generated longer regression-path durations on the adjective. In order to make sense of this finding, it is important to consider that the word *sale*, ‘dirty’, which shares its form with the third-person singular of *saler*, ‘to salt’, will be subject to lexical competition: on the one hand, the syntactic context suggests a simple SVO sentence, which favors the finite verb reading. However, the French Wikipedia corpus available at http://corp.hum.sdu.dk/cqp.fr.html (37.8 m words; [49]) shows the form *sale* to appear as an adjective 160 times, compared to only 9 occurrences as a finite verb. This implies that at least for the form *sale*, lexical frequency favors the adjective reading while the syntactic context favored the verb reading.

In order to further explore the influence of lexical competition on the ambiguity effect in TLA stimuli, we extracted token counts for both the verb and adjective readings of the relevant forms appearing in our items from the aforementioned Wikipedia corpus and computed the log ratio as a measure of bias toward the verb reading.

When entered as a covariate into the statistical analysis, the bias predictor showed no effect on first-pass reading times or regression-path durations for the adjective. In total reading times, in addition to a main effect of elision ($\hat{\beta }$ = 64 ms, CrI: [0 ms, 141 ms], Pr(*β* > 0) = 0.97) and a two-way interaction between ambiguity and elision ($\hat{\beta }$ = 132 ms, CrI: [−22 ms, 260 ms], Pr(*β* > 0) = 0.96), there was a two-way interaction between ambiguity and bias ($\hat{\beta }$ = −73 ms, CrI: [−150 ms, 4 ms], Pr(*β* > 0) = 0.02), such that a bias towards the verb reading led to longer reading times in the unambiguous conditions with adjective morphology only ($\hat{\beta }$ = 90 ms, CrI: [0 ms, 192 ms], Pr(*β* > 0) = 0.97).

When only total reading times prior to fixating the critical region were taken into account, there was evidence of a two-way interaction between bias and ambiguity ($\hat{\beta }$ = −83 ms, CrI: [−124 ms, −21 ms], Pr(*β* > 0) = 0.01), again driven by an effect of bias in the unambiguous conditions, such that verb-biased participles showed longer reading times in the presence of adjective morphology ($\hat{\beta }$ = 53 ms, CrI: [0 ms, 116 ms], Pr(*β* > 0) = 0.98). There were no effects of bias when only reading times after the first fixation on the critical region were taken into account.

#### Discussion

While ambiguous adjectives were more difficult to process overall, most likely due to increased lexical competition, it appears that participants perceived a clash between the lexical bias of the root form and the morphological adjective marker in the unambiguous conditions, which also caused processing difficulty. As there was no interaction with the elision manipulation and no evidence that lexical bias interacted with the experimental factors after participants had read the critical region, it appears that lexical bias probably exerted an effect on the initial analysis of the antecedent, but not on later attempts at re-parsing the structure. As before, these preliminary conclusions should be addressed in future confirmatory studies.

## Experiment 2

Experiment 1 yielded no evidence in favor of the reactivation hypothesis, which casts doubt on whether the results of Paape [1] are reproducible across different experimental paradigms and languages. In order to get a clearer picture, our second eye tracking study attempted to replicate the original result with the same materials and roughly the same number of participants, but using eye tracking instead of self-paced reading. If the eye tracking version of the German study yields comparable results, this would strengthen the claim that the pattern observed in the original experiment was not a mere artifact, and suggest that the diverging results in French are due to differences in the constructions investigated and not between the methods used.

### Materials

The experimental materials for Experiment 2 were taken directly from Paape [1]. Regions of interest were the same as in the original study. The factors word order (SVO vs. OVS) and case marking on the initial noun phrase (ambiguous vs. unambiguous) were manipulated in a 2 × 2 design, as shown below. As explained in the introduction, feminine nouns in German are not overtly marked for case, meaning that the first noun phrase of a main clause could either be the subject or the object, with the subject reading being preferred [15, 16]. The finite auxiliary *hatte/n*, ‘had’, agrees in number with either the first noun phrase, indicating that the word order is SVO, or with the second noun phrase, indicating that the word order is OVS.

(9)
  - **Ambiguous / OVS**
    Eine Sympathisantin der Opposition_np1_ ⋄ hatten_aux_ ⋄ die Rebellen_np2_ ⋄ …
    A sympathizer.fem.**nom|acc** of the opposition **had.pl** the rebels.nom|acc
  - **Ambiguous / SVO**
    Eine Sympathisantin der Opposition_np1_ ⋄ hatte_aux_ ⋄ die Rebellen_np2_ ⋄ …
    A sympathizer.fem.**nom|acc** of the opposition **had.sg** the rebels.nom|acc
  - **Unambiguous / OVS**
    Einen Sympathisanten der Opposition_np1_ ⋄ hatten_aux_ ⋄ die Rebellen_np2_ ⋄ …
    A sympathizer.masc.**acc** of the opposition **had.pl** the rebels.nom|acc
  - **Unambiguous / SVO**
    Ein Sympathisant der Opposition_np1_ ⋄ hatte_aux_ ⋄ die Rebellen_np2_ ⋄ …
    A sympathizer.masc.**nom** of the opposition **had.sg** the rebels.nom|acc
    … laut einem Bericht_adj_ ⋄ maßgeblich unterstützt,_vp_ ⋄ aber ⋄ die Regierung ⋄ konnte ⋄ nicht ⋄ nachweisen, ⋄ wie,_critical_ ⋄ so sehr ⋄ sich ⋄ die Untersuchungskommission ⋄ auch ⋄ bemühte.
     according to a report decisively supported but the government could not substantiate **how** so greatly SELF the investigative commission too struggled
    ‘The rebels had supported a sympathizer (OVS, a/c) / A sympathizer had supported the rebels (SVO, b/d), but the government could not substantiate how, no matter how hard the investigative commission tried.’

Sentences (9c, d) are control conditions in which the initial noun phrase is overtly marked for case, so that no additional processing difficulty is expected when the auxiliary agrees with the second noun phrase in (9c). As in the original study, but unlike in our Experiment 1, there were no control conditions without ellipsis in Experiment 2. A complete list of items is contained in S1 File.

### Predictions

If the change from self-paced reading to eye tracking does not have an influence on participants’ reading strategies, we expect to find the same pattern of results as in the original study of Paape [1]. First, a garden-path effect is expected at the auxiliary or at the second noun phrase, which should appear in the form of an interaction: in the original study, OVS sentences were especially difficult to process when the initial noun phrase carried ambiguous case marking, suggesting that readers first misanalyzed this noun phrase as being the subject.

At or directly after the critical ellipsis region, OVS sentences should be easier to process than SVO sentences in the ambiguous as compared to the unambiguous conditions, given that the reanalysis of the antecedent structure should lead to reactivation of the memory trace. In principle, this pattern may be visible in any of the eye tracking measures analyzed (first-pass reading time, regression-path duration, total reading time), as reading times from self-paced reading do not allow inferences as to whether an effect arises early or late in the processing stream.

Furthermore, Paape [1] observed the same interaction directly before the ellipsis site, which was interpreted as evidence for predictive processing: participants were argued to maintain an expectation of the upcoming ellipsis gap, effectively trying to pre-fill the gap before having encountered it. Paape also observed a processing disadvantage for OVS sentences in one of the ellipsis spillover regions, as well as a numerical tendency towards the same disadvantage both directly before and after the ellipsis site. This was interpreted as an effect of the mismatch between the OVS antecedent and the ellipsis gap, which sets a cue for an SOV antecedent (see introduction).

It is possible that the relative freedom of the eye tracking paradigm as compared to self-paced reading, especially with regard to the possibility of regressions, may change the pattern of results. For instance, readers may choose not to resolve the ambiguity in the initial clause, like the French participants did for TLA stimuli in Experiment 1, and experience processing difficulty at the ellipsis site because they do not have a fully analyzed representation of the antecedent available for retrieval. Such a result would not constitute evidence against the reactivation hypothesis per se, but would highlight the impact of different reading strategies on the probability of a correct retrieval at the critical region.

### Participants

We recruited 62 German native speakers as subjects. All had normal or corrected-to-normal eyesight and were either paid 7 € or received course credit for their participation. Informed consent was obtained from all participants prior to the experiment.

### Procedure

The procedure was analogous to that of Experiment 1, with the following changes. Eye movements were recorded by an SR Research Eyelink 1000 tracker with a desktop-mounted camera setup. Sentences were presented in 21 pt Arial font at a resolution of 1680 × 1050 pixels, with participants sitting at a distance of 61 cm from the presentation screen. The screen was 83.8 cm wide, so that each character displayed equaled 0.98° of visual angle. Recalibration of the eye-tracker was performed as needed when tracking accuracy fell below acceptable levels. Participants signaled completion of a trial by looking in the lower right corner of the screen for one second. Unlike in Experiment 1, filler sentences as well as experimental sentences were always followed by one of two types of comprehension test. The first type consisted of a statement that had to be judged as being either true or false while the second type required participants to fill in a gap within a statement, choosing one out of four options, as in Paape [1]. As in the original study, a subset of comprehension probes targeted the critical wh-pronoun while another subset targeted the argument structure of the antecedent.

### Data analysis

Data analysis was carried out analogously to Experiment 1. Again, the experimental factors were sum-coded, with ambiguous and OVS conditions coded as 1 and unambiguous and SVO conditions coded as −1, respectively. As in Experiment 1, a log transformation was applied to the dependent measures prior to analysis, and the log-transformed length of each region in letters was entered into the analysis as a covariate. Due to experimenter error, question response data from ten participants was lost, thus the reported accuracy and response latency results refer to the remaining 52 participants.

### Results

**Fig 7:**
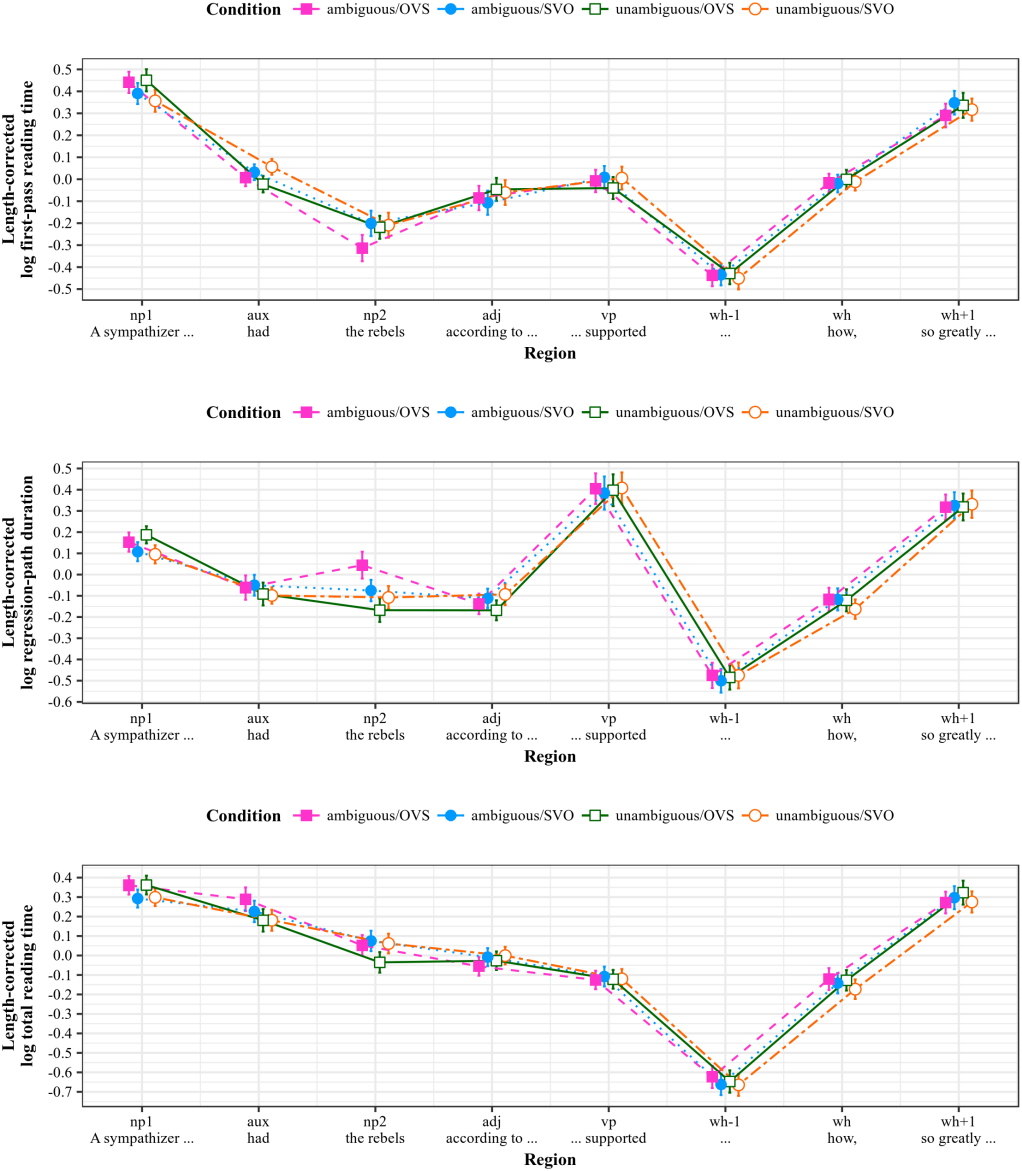
Reading measures by region (Experiment 2). All measures log-transformed and residualized against region length in characters; error bars show 95% intervals.

Length-corrected reading time measures by region of interest for Experiment 2 are shown in Fig 7.

#### Comprehension probes

Results for probe response accuracy and response latency are shown in Table 14. The overall probe response accuracy was 85%. Participants reached 92% accuracy when asked to supply the critical wh-pronoun and 76% accuracy when the argument structure of the antecedent was probed. There were no effects of the experimental manipulations on response times. For response accuracy, there was an effect of word order, such that probes about OVS sentences were answered less accurately than probes about SVO sentences ($\hat{\beta }=-3\%$, CrI: [−7%, 0%], Pr(*β* > 0) = 0.03).

**Table 14:** Experiment 2: Results for question response accuracy and response times. case = case marking, ord = word order.

response accuracy (logit scale)				
parameter	estimate	CrI low	CrI high	Pr(*β* > 0)
case	−0.05	−0.30	0.21	0.35
ord	−0.25	−0.53	0.01	0.03
case × ord	−0.19	−0.44	0.07	0.07
response time (log scale)				
parameter	estimate	CrI low	CrI high	Pr(*β* > 0)
case	0.00	−0.03	0.02	0.46
ord	0.01	−0.02	0.03	0.64
case × ord	0.01	−0.01	0.03	0.77

In order to check for possible parsing failures, which should result in comprehension probes being answered incorrectly, we ran an additional analysis in which the type of the comprehension probe was added to the accuracy and response time models. Argument structure probes were coded as 1 and other probes as −1. The results of this ancillary analysis are listed in Table 15.

**Table 15:** Experiment 2: Results for question response accuracy by probe type. case = case marking, ord = word order, pt = probe type.

response accuracy (logit scale)				
parameter	estimate	CrI low	CrI high	Pr(*β* > 0)
case	−0.08	−0.33	0.16	0.24
ord	−0.26	−0.51	−0.01	0.02
pt	−0.86	−1.24	−0.50	≈0.00
case × ord	−0.24	−0.47	−0.01	0.02
case × pt	−0.29	−0.53	−0.06	0.01
ord × pt	−0.36	−0.60	−0.12	≈0.00
case × ord × pt	−0.26	−0.50	−0.03	0.02
response time (log scale)				
parameter	estimate	CrI low	CrI high	Pr(*β* > 0)
case	−0.00	−0.03	0.02	0.45
ord	0.00	−0.03	0.04	0.59
pt	−0.11	−0.19	−0.04	≈0.00
case × ord	0.01	−0.02	0.03	0.73
case × pt	0.00	−0.02	0.03	0.56
ord × pt	−0.01	−0.04	0.02	0.34
case × ord × pt	0.00	−0.02	0.02	0.38

Response time only showed an effect of probe type, such that argument structure probes were responded to more quickly than the other probe types ($\hat{\beta }$ = −808 ms, CrI: [−1367 ms, −255 ms], Pr(*β* > 0) ≈ 0). With regard to accuracy, in addition to main effects of word order ($\hat{\beta }$ = −4%, CrI: [−8%, 0%], Pr(*β* > 0) = 0.02) and probe type ($\hat{\beta }$ = −14%, CrI: [−21%, −8%], Pr(*β* > 0) ≈ 0), along with all two-way interactions (case marking × word order: $\hat{\beta }$ = −14%, CrI: [−22%, −6%], Pr(*β* > 0) = 0.02, word order × probe type: $\hat{\beta }$ = −18%, CrI: [−27%, −10%], Pr(*β* > 0) ≈ 0, case marking × probe type: $\hat{\beta }$ = −16%, CrI: [−25%, −8%], Pr(*β* > 0) = 0.01), there was also a three-way interaction ($\hat{\beta }$ = −15%, CrI: [−24%, −7%], Pr(*β* > 0) = 0.02), such that ambiguous OVS sentences caused a much steeper drop in accuracy with argument structure probes than with the other probe types (56% vs. 92% mean accuracy).

#### First-pass reading times

First-pass reading times were longer for OVS compared to SVO sentences on the initial noun phrase ($\hat{\beta }$ = 102 ms, CrI: [39 ms, 165 ms], Pr(*β* > 0) ≈ 1), an effect that was reversed on the auxiliary ($\hat{\beta }$ = −19 ms, CrI: [−33 ms, −6 ms], Pr(*β* > 0) ≈ 0). The effect on the initial noun phrase is surprising given that there was no interaction with case marking, but may in part be due to parafoveal processing and/or misattributed fixations on the adjacent auxiliary, given that region’s relatively small size. The adjunct phrase showed shorter first-pass reading times in the ambiguous compared to the unambiguous conditions ($\hat{\beta }$ = −29 ms, CrI: [−58 ms, 1 ms], Pr(*β* > 0) = 0.03).

An interaction between word order and case marking became evident in the ellipsis spillover region ($\hat{\beta }$ = −35 ms, CrI: [−68 ms, 0 ms], Pr(*β* > 0) = 0.03), driven by shorter reading times in the ambiguous OVS compared to the ambiguous SVO condition ($\hat{\beta }$ = −22 ms, CrI: [−45 ms, −1 ms], Pr(*β* > 0) = 0.02). Table 16 shows parameter estimates across all regions of interest.

**Table 16:** Experiment 2: Results for first-pass reading times. case = case marking, ord = word order.

region	parameter	estimate	CrI low	CrI high	Pr(*β* > 0)
np1 *A sympathizer …*	case	0.01	−0.02	0.03	0.67
	ord	0.04	0.01	0.06	≈1.00
	case × ord	−0.01	−0.04	0.01	0.19
aux *had*	case	0.01	−0.02	0.03	0.72
	ord	−0.04	−0.06	−0.01	≈0.00
	case × ord	0.02	−0.01	0.04	0.93
np2 *the rebels*	case	−0.02	−0.05	0.01	0.06
	ord	−0.03	−0.07	0.01	0.08
	case × ord	−0.02	−0.05	0.01	0.06
adj *according to …*	case	−0.02	−0.05	0.00	0.03
	ord	0.01	−0.02	0.03	0.77
	case × ord	0.00	−0.02	0.03	0.64
vp *decisively supported,*	case	0.01	−0.01	0.04	0.85
	ord	−0.02	−0.04	0.01	0.07
	case × ord	0.01	−0.03	0.04	0.63
pre-crit *…*	case	0.00	−0.03	0.02	0.40
	ord	0.01	−0.01	0.04	0.82
	case × ord	−0.01	−0.03	0.02	0.34
crit *how,*	case	−0.01	−0.03	0.02	0.33
	ord	0.01	−0.02	0.03	0.72
	case × ord	0.00	−0.03	0.02	0.39
spillover *so greatly …*	case	−0.01	−0.04	0.02	0.28
	ord	−0.01	−0.04	0.02	0.24
	case × ord	−0.03	−0.05	0.00	0.03

#### Regression-path durations

At the second noun phrase, regression paths were longer in the ambiguous than in the unambiguous case marking conditions ($\hat{\beta }$ = 86 ms, CrI: [48 ms, 123 ms], Pr(*β* > 0) ≈ 1). There was also an interaction with word order ($\hat{\beta }$ = 124 ms, CrI: [44 ms, 202 ms], Pr(*β* > 0) ≈ 1), which was mainly driven by longer regression paths in the ambiguous OVS condition compared to the ambiguous SVO condition ($\hat{\beta }$ = 83 ms, CrI: [21 ms, 146 ms], Pr(*β* > 0) ≈ 1). At the adjunct, regression-path durations for OVS sentences were shorter than for SVO sentences ($\hat{\beta }$ = −41 ms, CrI: [−80 ms, −3 ms], Pr(*β* > 0) = 0.02). Results are shown in Table 17.

**Table 17:** Experiment 2: Results for regression-path durations. case = case marking, ord = word order.

region	parameter	estimate	CrI low	CrI high	Pr(*β* > 0)
np1 *A sympathizer …*	See first-pass reading times				
aux *had*	case	0.02	−0.01	0.05	0.89
	ord	−0.01	−0.04	0.02	0.28
	case × ord	−0.01	−0.04	0.02	0.29
np2 *the rebels*	case	0.06	0.04	0.09	≈1.00
	ord	0.02	−0.01	0.04	0.86
	case × ord	0.04	0.01	0.07	≈1.00
adj *according to …*	case	0.00	−0.02	0.03	0.59
	ord	−0.03	−0.05	0.00	0.02
	case × ord	0.01	−0.01	0.03	0.82
vp *decisively supported,*	case	0.00	−0.03	0.04	0.52
	ord	0.00	−0.05	0.04	0.45
	case × ord	0.00	−0.04	0.05	0.55
pre-crit *…*	case	−0.01	−0.04	0.03	0.31
	ord	0.01	−0.02	0.04	0.79
	case × ord	0.01	−0.02	0.05	0.75
crit *how,*	case	0.01	−0.02	0.04	0.71
	ord	0.01	−0.02	0.04	0.78
	case × ord	0.00	−0.03	0.03	0.48
spillover *so greatly …*	case	0.01	−0.02	0.04	0.75
	ord	0.01	−0.02	0.04	0.68
	case × ord	−0.02	−0.05	0.01	0.13

#### Total reading times

Table 18 lists results for total reading times. The initial noun phrase showed increased total reading times in OVS compared to SVO sentences ($\hat{\beta }$ = 156 ms, CrI: [60 ms, 257 ms], Pr(*β* > 0) ≈ 1). The auxiliary showed an effect of case marking, such that reading times were longer for sentences with ambiguous case marking ($\hat{\beta }$ = 33 ms, CrI: [5 ms, 60 ms], Pr(*β* > 0) = 0.99). The ambiguity effect remained in evidence on the second noun phrase ($\hat{\beta }$ = 48 ms, CrI: [−6 ms, 100 ms], Pr(*β* > 0) = 0.96), but the order effect was reversed, such that OVS sentences showed shorter total reading times than SVO sentences ($\hat{\beta }$ = −58 ms, CrI: [−111 ms, 8 ms], Pr(*β* > 0) = 0.01).

**Table 18:** Experiment 2: Results for total reading times. case = case marking, ord = word order.

region	parameter	estimate	CrI low	CrI high	Pr(*β* > 0)
np1 *A sympathizer …*	case	0.00	−0.03	0.02	0.45
	ord	0.03	0.01	0.06	≈1.00
	case × ord	0.00	−0.02	0.02	0.53
aux *had*	case	0.04	0.01	0.07	0.99
	ord	0.02	−0.01	0.05	0.91
	case × ord	0.02	−0.01	0.04	0.90
np2 *the rebels*	case	0.02	0.00	0.05	0.96
	ord	−0.03	−0.06	0.00	0.01
	case × ord	0.02	−0.01	0.04	0.92
adj *according to …*	case	−0.01	−0.03	0.01	0.17
	ord	−0.02	−0.04	0.00	0.06
	case × ord	−0.01	−0.03	0.02	0.34
vp *decisively supported,*	case	0.00	−0.02	0.03	0.61
	ord	−0.01	−0.03	0.02	0.32
	case × ord	0.00	−0.03	0.02	0.39
pre-crit *…*	case	0.01	−0.02	0.04	0.69
	ord	0.02	−0.02	0.05	0.81
	case × ord	0.00	−0.03	0.03	0.62
crit *how,*	case	0.01	−0.01	0.03	0.78
	ord	0.02	−0.01	0.05	0.90
	case × ord	−0.01	−0.03	0.02	0.31
spillover *so greatly …*	case	−0.01	−0.04	0.02	0.26
	ord	0.01	−0.02	0.03	0.70
	case × ord	−0.02	−0.05	0.00	0.05

As in first-pass reading times, the ellipsis spillover region showed an interaction between case marking and word order ($\hat{\beta }$ = −39 ms, CrI: [−83 ms, 8 ms], Pr(*β* > 0) = 0.05), in this case driven by neither pair of conditions.

### Design analysis

**Fig 8:**
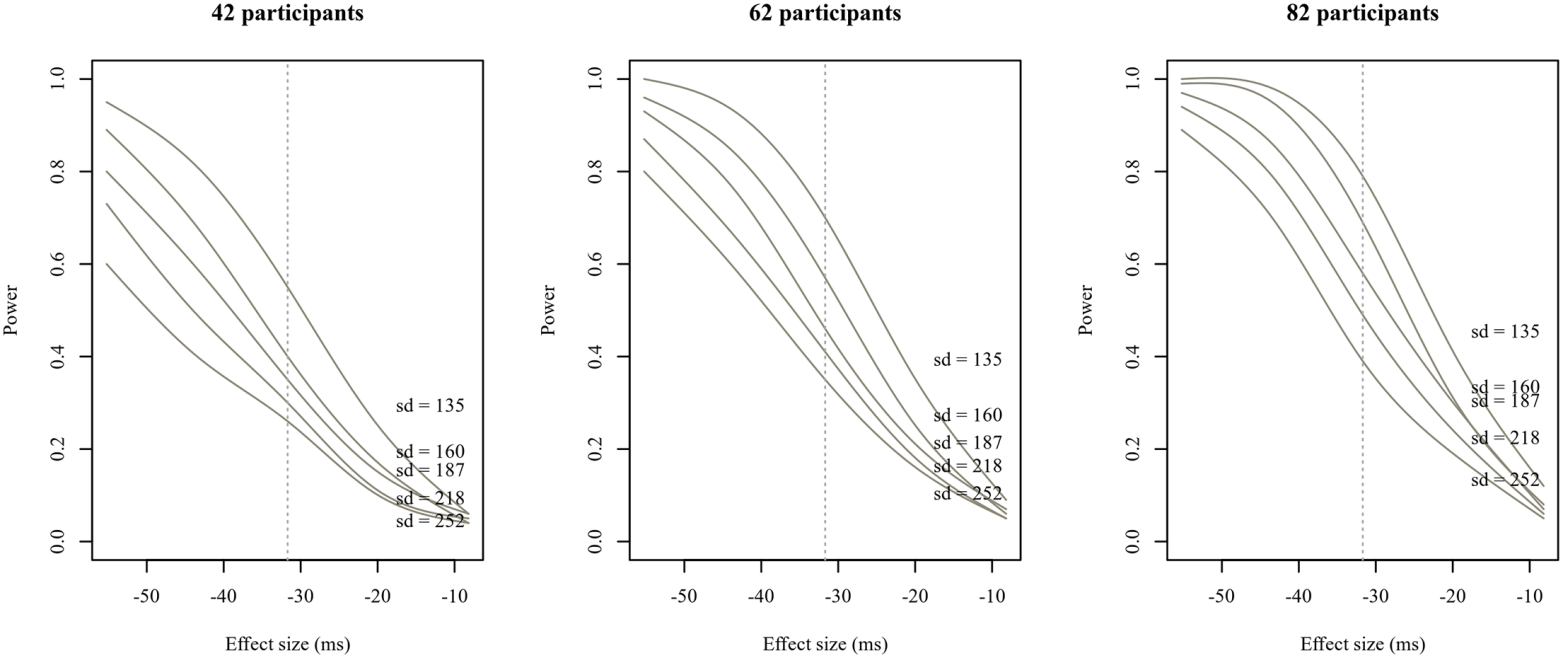
Estimated power (smoothed) as a function of interaction size, sample size and residual variance in total reading times at the critical region.

As for Experiment 1, we conducted a simulation-based design analysis based on parameter estimates from the data. This time we focused on the interaction observed in first-pass reading times at the spillover region, as it is most directly relevant for the claims of the reaction hypothesis. As before, 1000 new data sets were created for each unique combination of effect size, sample size and residual variance. Fig 8 shows the results.

As can be seen in the graphic, under the assumption that the estimated effect size is close to the ‘true’ effect size, the experiment had about 50% power to detect it. Perhaps even more discouragingly, increasing our sample size by 20 participants would only have increased power by about 10%. Power would, of course, be still lower if the observed effect size is an overestimate. Given these observations, there is a clear need for further replication attempts with substantially larger sample sizes in the future (see also [19] for a more general point about replication).

#### Reading times before and after the first fixation on the critical region

As before, we analyzed total reading times before and after the first fixation on the critical region separately in order to get a clearer picture of participants’ behavior.

When only fixations made after having fixated the critical region were taken into account, there were no effects of the experimental manipulations on either re-reading probabilities or re-reading times when the first three regions of the antecedent—that is, the two argument noun phrases and the auxiliary—were analyzed together, nor when each region was analyzed separately.

When only fixations made prior to visiting the ellipsis site were taken into account, OVS word order led to longer total reading times on the initial noun phrase ($\hat{\beta }$ = 154 ms, CrI: [77 ms, 269 ms], Pr(*β* > 0) ≈ 1), an effect that was also visible on the auxiliary ($\hat{\beta }$ = 60 ms, CrI: [38 ms, 83 ms], Pr(*β* > 0) ≈ 1), but reversed on the second noun phrase ($\hat{\beta }$ = −47 ms, CrI: [−78 ms, 0 ms], Pr(*β* > 0) = 0.02).

Pre-critical total reading times on the auxiliary also showed a main effect of case marking, such that reading times were longer in the ambiguous conditions ($\hat{\beta }$ = 30 ms, CrI: [8 ms, 53 ms], Pr(*β* > 0) ≈ 1), as well as an interaction indicative of a garden-path effect in the ambiguous OVS condition ($\hat{\beta }$ = 35 ms, CrI: [5 ms, 80 ms], Pr(*β* > 0) = 0.95). The second noun phrase showed the same main effect of case marking ($\hat{\beta }$ = 47 ms, CrI: [16 ms, 94 ms], Pr(*β* > 0) = 0.99) and the same interaction ($\hat{\beta }$ = 60 ms, CrI: [−3 ms, 154 ms], Pr(*β* > 0) = 0.95). Results of the pre-ellipsis analysis thus closely match those of the overall analysis, with the exception of the interaction observed at the auxiliary which was not in evidence when the entire trial was taken into consideration.

### Discussion

By and large, the results of Experiment 2 are similar to those of Paape [1], though there are some important differences. The garden-path effect observed on the second noun phrase in the original self-paced reading experiment was visible at the same location in regression-path durations in the current study, suggesting that participants adopted the erroneous subject reading of the initial noun phrase in ambiguous OVS sentences and were then forced to reanalyze. The crucial interaction between word order and case marking that is predicted by the reactivation hypothesis was visible in first-pass reading times at the ellipsis spillover region. Numerically, it was of the same shape as in Paape [1]; however, in the current study it was driven mainly by the ambiguous conditions. An interaction was also observed in total reading times, where it was not driven by the ambiguous conditions, just as in the original experiment.

Comprehension accuracy was comparable across the studies, even down to the changing accuracy patterns with different comprehension probes. In contrast to Paape [1], we observed a steep drop in accuracy when the comprehension probe targeted the antecedent’s argument structure in garden-path sentences. This pattern may imply that on some trials the garden path was not resolved correctly, resulting in an incorrect representation of the antecedent. However, as the reduced accuracy did not coincide with an increase in total reading times or re-reading probabilities/times for any of the relevant antecedent regions or for the ellipsis site, which might have been expected given the results from Experiment 1, we are left with the question why participants did not check their interpretations against the input more diligently, a point to which we return below.

Unlike in the original study, the current experiment yielded no evidence in favor of predictive processing in the region directly before the critical region, which would have indicated that participants were expecting an elided structure. It is not clear why such an effect would appear in self-paced reading but not in eye tracking. One possible explanation would be that self-paced reading generally puts more pressure on the processing system due to the impossibility of regressions (and possibly the lack of parafoveal preview), and that part of the computational load is shifted towards prediction in order to minimize the impact of unexpected continuations. Furthermore, unlike eye tracking data, self-paced reading data is composed of a series of response times with non-zero shift, due to the time it takes to perform the motor action, that is, press the space bar [50, 51]. Speculatively, additional predictive processing may be carried out during this ‘idle time’.

Interestingly, the adjunct region of the antecedent showed evidence of a speedup in regression-path durations in OVS compared to SVO sentences. A similar speedup was also observed in the original self-paced reading study, where Paape [1] speculated that it may be due to readers trying to make up for lost time after having been slowed down by the non-canonical word order in the preceding regions.

**Fig 9:**
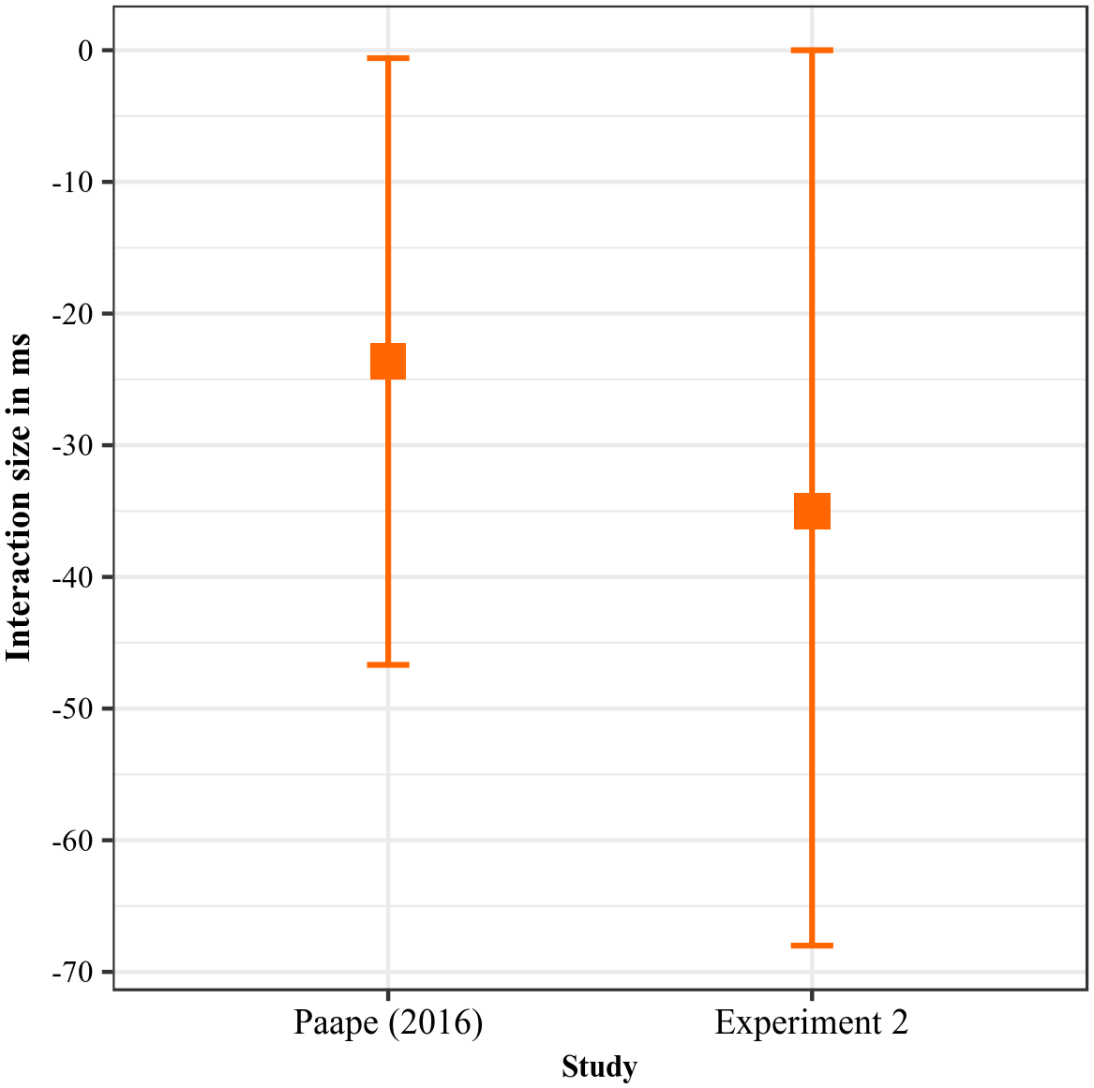
Comparison of effects between Paape [1] and Experiment 2.

In order to gauge the magnitude of the critical interaction predicted by the reactivation hypothesis that was observed in first-pass reading times at the spillover region in Experiment 2, we compared it to the interaction found by Paape in self-paced reading times in the third spillover region. We back-transformed both parameter estimates and their credible and confidence intervals, respectively, to the millisecond scale, as shown in Fig 9. We chose first-pass as opposed to total reading times as the basis for comparison because self-paced reading does not allow re-reading, leading us to assume that the earliest observed effect may be the most parallel to the one observed in the original study.

As the figure shows, the parameter estimates fall between −35 ms (CrI: [−68 ms, 0 ms]) in the current study and −24 ms (CI: [−47 ms, −1 ms]) in the original study. The current study thus showed a larger estimate of the mean interaction effect, but even higher uncertainty than the original study with regard to the actual magnitude, and showed a non-zero (if low) probability of the effect being zero or positive.

Taken at face value, our result suggests that just like in the original study, having refreshed the antecedent’s memory trace through reanalysis made the trace easier to retrieve during the interpretation of the ellipsis. Yet, there are two critical discrepancies in comparison with the original study: first, no OVS mismatch effect was observed in the overall analysis, which would suggest that the amount of fit between the gap’s retrieval cue for SOV word order and the ellipsis antecedent did not influence processing at the ellipsis site. Second, as the garden-path condition showed poor comprehension accuracy, we cannot be sure that the antecedent was always retrieved correctly.

It should be noted that the reduced response accuracy in the garden-path condition, where the speedup at the critical region was observed, is not necessarily evidence against the reactivation account. Additional ‘post-interpretive processing’ may be carried out when the comprehension probe is presented [52], which may involve factors beyond accessing the interpretation that was derived during reading. This type of processing may be more prone to retrieving remnants of garden-path structures than ‘true’ on-line processes (see [53]).

Assuming that response accuracy *does* directly reflect parsing success, we can ask why we would observe a speedup at the ellipsis site if the antecedent was not parsed correctly, given that the findings of Experiment 1 suggest that a malformed antecedent representation leads to a slowdown at the ellipsis site.

Paape [1] briefly considers an alternative explanation of the ambiguity advantage at the ellipsis site which rests on the assumption that discarded parses may remain active to some degree, causing comprehension errors [53–56]. Under such an account, the parser would sometimes retrieve the initial, erroneous SVO representation of the first clause in the ambiguous OVS condition as the ellipsis antecedent without noticing the mistake. Processing times for the ellipsis would then be predicted to decrease, given that an SVO structure is a better match for the SOV cue from the ellipsis site than the OVS structure.

In light of the accuracy results in the current study, the possibility that participants sometimes retrieved an incorrect SVO structure in ambiguous OVS clauses cannot be discounted. Such an account, however, would raise the question why the French readers in Experiment 1 did not retrieve and integrate an incorrect structure but rather returned to the antecedent and attempted to re-parse it. We take up this question in the general discussion.

In order to check whether there was any connection between processing times at the ellipsis site and probe response accuracy in Experiment 2, we performed separate analyses of first-pass reading times at the ellipsis spillover region, where the supposed reactivation effect appeared, conditional on response accuracy and probe type—that is, whether the probe targeted the assignment of thematic roles in the antecedent or not.

If the speedup was really due to erroneous retrievals, it should be most clearly visible when the subject gives an inaccurate response to a thematic role probe. This was not the case: the only subset of the data which by itself showed an interaction between word order and case marking was the one in which non-thematic role probes were responded to incorrectly, as shown by a post-hoc ancillary analysis ($\hat{\beta }$ = −232 ms, CrI: [−388 ms, −77 ms], Pr(*β* > 0) ≈ 0). While it should be kept in mind that this particular subset consisted of only 80 observations from 39 subjects and 17 items (as probe type was a between-items factor), the finding could be taken to imply that subjects were less able to remember meaning aspects that were *not* related to the antecedent and the ellipsis because they had focused on resolving the ellipsis correctly. In any case, we have no evidence that the garden-path effect on probe response accuracy was directly related to the speedup at the ellipsis spillover region.

In summary, the main prediction of the reactivation hypothesis was borne out in Experiment 2. However, the pattern seen in participants’ overall response accuracy as well as the lack of an OVS mismatch effect at the ellipsis site cast some doubt on the result, though new problems arise when parsing failure is assumed to be responsible for the speedup, as shown by the incongruous result of the ancillary analysis.

## General discussion

We have presented two eye tracking studies which were intended to test the predictions of the reactivation hypothesis of Paape [1], which claims that an ellipsis antecedent becomes easier to retrieve if it has been syntactically reanalyzed at an earlier point in the sentence, due to the memory trace having received an activation boost. Experiment 1, carried out in French with three different kinds of garden-path antecedents, did not yield any evidence in favor of the reactivation hypothesis. Instead, there was some indication that, at least for one type of garden path, participants did not resolve the temporary syntactic ambiguity of the antecedent, which led to processing difficulty at the ellipsis site. Experiment 2, which was an attempt to replicate Paape [1]’s original study, yielded results that were compatible with the reactivation hypothesis; some aspects of the data, however, would allow for a different explanation of the ambiguity-induced speedup, namely that subjects may occasionally have misretrieved the initial, erroneous representation of the antecedent.

It has recently been suggested that failures to retrieve a memory target during dependency resolution may speed up processing instead of slowing it down. Nicenboim and colleagues [51] found that inserting additional material between two elements of a long-distance dependency led to a slowdown in reading times for subjects with high working memory capacity, but, contrary to prediction, caused a speedup for subjects with low working memory capacity. The authors explain the finding by assuming that low-capacity readers have a higher rate of memory retrieval failure in the presence of intervening words, and that such failures are faster on average than successful retrievals. As Nicenboim et al. [51] did not investigate garden-path structures, there was no opportunity for participants to retrieve a non-target alternative representation, as may have been the case in our Experiment 2. Nevertheless, the results call into question the implicit assumption that the time taken to process a given region is proportional to the rate of success at correctly deriving the relevant meaning.

Relatedly, Slattery and colleagues [56] investigated small discourses like the one shown in (10), where the first sentence contains an early-closure ambiguity:

(10)
  While Frank dried off the truck that was dark green was peed on by a stray dog. Frank quickly finished drying himself off then yelled out the window at the dog.

While the results showed evidence of reanalysis in regression-path durations for the disambiguating region (*was peed on …*), first-pass reading times for the second clause were increased compared to a control condition, suggesting that participants perceived a clash between their initial misinterpretation (*Frank dried off the truck*) and the continuation of the discourse. This, in turn, indicates that despite participants’ attempts at reanalysis, the garden-path reading still continued to interfere with processing, possibly because it was not completely inhibited.

Looking again at our Experiment 2, the situation is different from the one in (10), as the ellipsis is compatible with the correct as well as the garden-path reading of the antecedent. We would thus not expect a slowdown in processing, as there is no semantic clash, but may instead observe a speedup, because the parser has an alternative retrieval target available which yields an unlicensed but nonetheless grammatical interpretation.

In Experiment 1, a measurable effect at the ellipsis site was only observed for one stimulus type, namely for sentences with triple lexical ambiguity (TLA stimuli). For these stimuli, there was no evidence of reanalysis in the disambiguating region of the antecedent; instead, processing times were increased for the ambiguous region, suggesting competition between the different readings. Furthermore, the ambiguous condition was not easier, but rather more difficult to process at the ellipsis site, which implies that participants were having trouble completing any retrieval at all.

Taken together, the two patterns suggest that a kind of ‘good-enough’ processing (e.g. [57]) was employed in Experiment 1, at least on some trials: despite evidence that the ambiguity was noticed by subjects, sometimes they nevertheless continued reading past the antecedent clause without resolving it, hazarding the consequences of not fully grasping the intended meaning of the whole stimulus sentence.

Note that for such a strategy to be, in fact, good enough, participants would need to have an expectation of still being able to answer the comprehension probes with a reasonable level of accuracy. Given that not all comprehension probes targeted the interpretation of the antecedent or the ellipsis site, this is not an implausible assumption. Had the wrong meaning been derived, no problem would have arisen at the ellipsis site, just as in Experiment 2, given that the ellipsis itself is compatible with both readings. However, it appears that TLA stimuli occasionally created a situation in which participants did not succeed at settling on a reading, resulting in underspecification of the antecedent’s structure [25]. It would appear that underspecified structures are not valid retrieval targets, given that participants regressed to the antecedent, presumably in order to attempt a re-parse.

A final remark concerns the absence of a measurable effect of antecedent reanalysis on ellipsis processing times in the remaining French sentences, that is, for subject-object inversion and reduced relative clauses. Based on comments from participants, we have argued that the inversion stimuli may sometimes have been judged as ungrammatical, which would have masked any reactivation effect, especially as the distance between the disambiguating word and the ellipsis site was much shorter than in the other stimuli. As for the reduced relative clauses, we have argued that reanalysis may not always have been carried out, presumably because the would-be garden-path structure was in fact the preferred one. However, these post-hoc hypotheses are clearly unsatisfying and need to be tested more rigorously.

To conclude, we found evidence compatible with the reactivation hypothesis only in one out of four cases of antecedent ambiguity, specifically in the same construction originally used by Paape [1]. Given the aforementioned caveats, however, we cannot be entirely certain that reactivation is indeed taking place in this case. We also found evidence suggesting that when the antecedent has a particularly difficult ambiguous structure, participants may be unable to resolve the ambiguity and end up without a retrieval target at the ellipsis site. One fruitful approach for future studies would be to probe the interpretation of the antecedent on-line, possibly in a cross-modal priming or visual world setup, so that the participants’ success at parsing the garden-path structure can be assessed on a trial-by-trial basis.

## Supporting information

S1 File Appendices I and II. (PDF)

S2 File Acceptability rating data (Pilot study). (TXT)

S3 File Eye tracking data (Experiment 1). (TXT)

S4 File Question response data (Experiment 1). (TXT)

S5 File Eye tracking data, after critical region (Experiment 1). (TXT)

S6 File Eye tracking data, before critical region (Experiment 1). (TXT)

S7 File Lexical bias data (Experiment 1). (TXT)

S8 File R analysis script for rating data (Pilot study). (R)

S9 File Stan model for rating data (Pilot study). (STAN)

S10 File R analysis script for question responses (Experiment 1). (R)

S11 File Stan model for question response accuracy (Experiment 1). (STAN)

S12 File Stan model for question response times (Experiment 1). (STAN)

S13 File R analysis script for eye tracking data (Experiment 1). (R)

S14 File Stan model for eye tracking data (Experiment 1). (STAN)

S15 File R script for generating plots (Experiment 1). (R)

S16 File Eye tracking data (Experiment 2). (TXT)

S17 File Question response data (Experiment 2). (TXT)

S18 File Eye tracking data, after critical region (Experiment 2). (TXT)

S19 File Eye tracking data, before critical region (Experiment 2). (TXT)

S20 File R analysis script for question responses (Experiment 2). (R)

S21 File R analysis script for eye tracking data (Experiment 2). (R)

S22 File Stan model for eye tracking data (Experiment 2). (STAN)

S23 File R script for generating plots (Experiment 2). (R)

S24 File R script for comparison between Experiment 2 and Paape (2016). (R)

S25 File R script for design analyses (Experiments 1 and 2). (R)
