# Supplementary material for: Processing of ellipsis with garden-path antecedents in French and German: Evidence from eye tracking
Source: PLoS One. 2018 Jun 13;13(6):e0198620. doi: 10.1371/journal.pone.0198620 (PMC5999118; doi:10.1371/journal.pone.0198620)
Supplement: S1 File — (PDF) [file pone.0198620.s001.pdf]

Processing of ellipsis with garden-path antecedents in French and German: Evidence from eye tracking
Appendices

Dario Paape[1*], Barbara Hemforth[2], Shravan Vasishth[1]

1 Department of Linguistics, University of Potsdam, Potsdam, Germany
2 Laboratoire de Linguistique Formelle, Université Paris Diderot, Paris, France

* paape@uni-potsdam.de

Appendix I – Items used in Experiment 1

Vertical bars indicate boundaries between regions of interest.

RRC stimuli

1. a. Le politicien | contredit | durant le débat | a proposé | des mesures | à réaliser | en vitesse, | mais tous les quotidiens | de tendance libérale | se demandaient | pourquoi, | vu que | ces derniers temps | il n'y a pas eu | de menaces terroristes | à prendre au sérieux.
   b. La politicienne | contredite | durant le débat | a proposé | des mesures | à réaliser | en vitesse, | mais tous les quotidiens | de tendance libérale | se demandaient | pourquoi, | vu que | ces derniers temps | il n'y a pas eu | de menaces terroristes | à prendre au sérieux.
   c. Le politicien | contredit | durant le débat | a proposé | des mesures | à réaliser | en vitesse, | mais tous les quotidiens | de tendance libérale | les critiquaient, | vu que | ces derniers temps | il n'y a pas eu | de menaces terroristes | à prendre au sérieux.
   d. La politicienne | contredite | durant le débat | a proposé | des mesures | à réaliser | en vitesse, | mais tous les quotidiens | de tendance libérale | les critiquaient, | vu que | ces derniers temps | il n'y a pas eu | de menaces terroristes | à prendre au sérieux.
2. a. Le héros | décrit | dans cette histoire | a vaincu | une bête féroce, | mais aucun conte | du romancier | ne mentionne | comment, | peut-être | parce qu'il | n'était pas | présent à l'époque.
   b. Les héros | décrits | dans cette histoire | ont vaincu | une bête féroce, | mais aucun conte | du romancier | ne mentionne | comment, | peut-être | parce qu'il | n'était pas | présent à l'époque.
   c. Le héros | décrit | dans cette histoire | a vaincu | une bête féroce, | mais aucun conte | du romancier | ne le mentionne, | peut-être | parce qu'il | n'était pas | présent à l'époque.
   d. Les héros | décrits | dans cette histoire | ont vaincu | une bête féroce, | mais aucun conte | du romancier | ne le mentionne, | peut-être | parce qu'il | n'était pas | présent à l'époque.

3. a. Le navire | détruit | pendant la guerre | avait rejoint | le port, | mais le professeur | d'histoire | ne pouvait pas dire | quand, | laissant | un peu désillusionnée | la jeune collègue | durant leur rendez-vous.
   b. Les navires | détruits | pendant la guerre | avaient rejoint | le port, | mais le professeur | d'histoire | ne pouvait pas dire | quand, | laissant | un peu désillusionnée | la jeune collègue | durant leur rendez-vous.
   c. Le navire | détruit | pendant la guerre | avait rejoint | le port, | mais le professeur | d'histoire | n'en savait rien, | laissant | un peu désillusionnée | la jeune collègue | durant leur rendez-vous.
   d. Les navires | détruits | pendant la guerre | avaient rejoint | le port, | mais le professeur | d'histoire | n'en savait rien, | laissant | un peu désillusionnée | la jeune collègue | durant leur rendez-vous.
4. a. L'étudiant | distrait | pendant la classe | était parti, | mais même le directeur | de l'école | ne savait pas | pourquoi, | puisqu'il | n'y avait pas | de lettre | ni d'avertissement.
   b. L'étudiante | distraite | pendant la classe | était partie, | mais même le directeur | de lécole | ne savait pas | pourquoi, | puisqu'il | n'y avait pas | de lettre | ni d'avertissement.
   c. L'étudiant | distrait | pendant la classe | était parti, | mais même le directeur | de l'école | ne le savait pas, | puisqu'il | n'y avait pas | de lettre | ni d'avertissement.
   d. L'étudiante | distraite | pendant la classe | était partie, | mais même le directeur | de l'école | ne le savait pas, | puisqu'il | n'y avait pas | de lettre | ni d'avertissement.
5. a. Le dernier chapitre | soustrait | du livre | avait | l'air ingénieux, | mais effectivement | personne | ne pouvait dire | exactement | pourquoi, | d'autant que | beaucoup d'intellectuels | n'avaient même pas | compris l'introduction.
   b. Les derniers chapitres | soustraits | du livre | avaient | l'air ingénieux, | mais effectivement | personne | ne pouvait dire | exactement | pourquoi, | d'autant que | beaucoup d'intellectuels | n'avaient même pas | compris l'introduction.
   c. Le dernier chapitre | soustrait | du livre | avait | l'air ingénieux, | mais effectivement | personne | ne le savait, | d'autant que | beaucoup d'intellectuels | n'avaient même pas | compris l'introduction.
   d. Les derniers chapitres | soustraits | du livre | avaient | l'air ingénieux, | mais effectivement | personne | ne le savait, | d'autant que | beaucoup d'intellectuels | n'avaient même pas | compris l'introduction.
6. a. L'enseignant | inscrit | au cours de mathématiques avancées | s'était tué, | mais le porte-parole | de la police | n'avait pas dit | comment, | de sorte que | les villageois | au café | ne pouvaient que spéculer.
   b. L'enseignante | inscrite | au cours de mathématiques avancées | s'était tuée, | mais le porte-parole | de la police | n'avait pas dit | comment, | de sorte que | les villageois | au café | ne pouvaient que spéculer.
   c. L'enseignant | inscrit | au cours de mathématiques avancées | s'était tué, | mais le porte-parole | de la police | n'en avait | rien révélé, | de sorte que | les villageois | au café | ne pouvaient que spéculer.
   d. L'enseignante | inscrite | au cours de mathématiques avancées | s'était tuée, | mais le porte-parole | de la police | n'en avait | rien révélé, | de sorte que | les villageois | au café | ne pouvaient que spéculer.
7. a. Le secrétaire | instruit | de la défaite | aurait été trouvé | dans un bar, | mais sur la base du rapport militaire | il est impossible | de dire | par qui, | d'autant que | les passages pertinents | ont été noircis.
   b. La secrétaire | instruite | de la défaite | aurait été trouvée | dans un bar, |

mais sur la base du rapport militaire | il est impossible | de dire | par qui, | d'autant que | les passages pertinents | ont été noircis.

c. Le secrétaire | instruit | de la défaite | aurait été trouvé | dans un bar, | mais sur la base du rapport militaire | il est impossible | de le vérifier, | d'autant que | les passages pertinents | ont été noircis.

d. La secrétaire | instruite | de la défaite | aurait été trouvée | dans un bar, | mais sur la base du rapport militaire | il est impossible | de le vérifier, | d'autant que | les passages pertinents | ont été noircis.

8. a. Le fonctionnaire | interdit | de séance | a reçu | une convocation, | mais aucun de ses collègues | ne pouvait encore | expliquer | pourquoi, | jusqu'à ce que | la police secrète | l'arrête | pour haute trahison.

b. La fonctionnaire | interdite | de séance | a reçu | une convocation, | mais aucun de ses collègues | ne pouvait encore | expliquer | pourquoi, | jusqu'à ce que | la police secrète | l'arrête | pour haute trahison.

c. Le fonctionnaire | interdit | de séance | a reçu | une convocation, | mais aucun de ses collègues | ne pouvait encore | l'expliquer, | jusqu'à ce que | la police secrète | l'arrête | pour haute trahison.

d. La fonctionnaire | interdite | de séance | a reçu | une convocation, | mais aucun de ses collègues | ne pouvait encore | l'expliquer, | jusqu'à ce que | la police secrète | l'arrête | pour haute trahison.

9. a. Le fruit exotique | introduit | au village | était | fortement toxique, | mais sur le moment | les scientifiques de l'université | n'avaient pas | découvert | pourquoi, | alors même que | le gouvernement | leur avait fourni | des moyens financiers considérables.

b. Les fruits exotiques | introduits | au village | étaient | fortement toxiques, | mais sur le moment | les scientifiques de l'université | n'avaient pas | découvert | pourquoi, | alors même que | le gouvernement | leur avait fourni | des moyens financiers considérables.

c. Le fruit exotique | introduit | au village | était | fortement toxique, | mais sur le moment | les scientifiques de l'université | ne l'avaient pas | découvert, | alors même que | le gouvernement | leur avait fourni | des moyens financiers considérables.

d. Les fruits exotiques | introduits | au village | étaient | fortement toxiques, | mais sur le moment | les scientifiques de l'université | ne l'avaient pas | découvert, | alors même que | le gouvernement | leur avait fourni | des moyens financiers considérables.

10. a. L'artiste | peint | dans son atelier | avait rencontré | une comtesse, | mais le guide avait malheureusement oublié | de vérifier | où, | de sorte que | les touristes | se sont plaints | à l'agence de voyage.

b. L'artiste | peinte | dans son atelier | avait rencontré | une comtesse, | mais le guide avait malheureusement oublié | de vérifier | où, | de sorte que | les touristes | se sont plaints | à l'agence de voyage.

c. L'artiste | peint | dans son atelier | avait rencontré | une comtesse, | mais le guide avait malheureusement oublié | d'en vérifier | le lieu, | de sorte que | les touristes | se sont plaints | à l'agence de voyage.

d. L'artiste | peinte | dans son atelier | avait rencontré | une comtesse, | mais le guide avait malheureusement oublié | d'en vérifier | le lieu, | de sorte que | les touristes | se sont plaints | à l'agence de voyage.

11. a. Le roi éternel | prédit | dans les écritures | aurait accompli | un miracle, | mais de nos jours il est difficile | de dire | quand, | en particulier | parce que | la Curie refuse | de rendre accessibles | ses archives.

b. La reine éternelle | prédite | dans les écritures | aurait accompli | un miracle,

| mais de nos jours il est difficile | de dire | quand, | en particulier | parce que | la Curie refuse | de rendre accessibles | ses archives.

c. Le roi éternel | prédit | dans les écritures | aurait accompli | un miracle, | mais de nos jours il est difficile | d'en découvrir | la nature, | en particulier | parce que | la Curie refuse | de rendre accessibles | ses archives.

d. La reine éternelle | prédite | dans les écritures | aurait accompli | un miracle, | mais de nos jours il est difficile | d'en découvrir | la nature, | en particulier | parce que | la Curie refuse | de rendre accessibles | ses archives.

12. a. Le robot | produit | à l'usine | doit traiter | des pièces uniques, | mais | pour le chef d'équipe c'est un vrai problème | de décider | quand, | vu qu'il y a | plein d'autres | commandes importantes | à finir.

b. Les robots | produits | à l'usine | doivent traiter | des pièces uniques, | mais | pour le chef d'équipe c'est un vrai problème | de décider | quand, | vu qu'il y a | plein d'autres | commandes importantes | à finir.

c. Le robot | produit | à l'usine | doit traiter | des pièces uniques, | mais | pour le chef d'équipe c'est un vrai problème | d'en trouver | le temps, | vu qu'il y a | plein d'autres | commandes importantes | à finir.

d. Les robots | produits | à l'usine | doivent traiter | des pièces uniques, | mais | pour le chef d'équipe c'est un vrai problème | d'en trouver | le temps, | vu qu'il y a | plein d'autres | commandes importantes | à finir.

13. a. Le texte | reconstruit | à partir d'anciennes épigraphes | contient | des contradictions, | mais pour les philosophes des temps modernes il est difficile | d'établir | lesquelles, | car les penseurs | de la Renaissance | ont un style | trop opaque.

b. Les textes | reconstruits | à partir d'anciennes épigraphes | contiennent | des contradictions, | mais pour les philosophes des temps modernes il est difficile | d'établir | lesquelles, | car les penseurs | de la Renaissance | ont un style | trop opaque.

c. Le texte | reconstruit | à partir d'anciennes épigraphes | contient | des contradictions, | mais pour les philosophes des temps modernes il est | difficile | de les établir, | car les penseurs | de la Renaissance | ont un style | trop opaque.

d. Les textes | reconstruits | à partir d'anciennes épigraphes | contiennent | des contradictions, | mais pour les philosophes des temps modernes il est | difficile | de les établir, | car les penseurs | de la Renaissance | ont un style | trop opaque.

14. a. Le sultan | satisfait | depuis peu | avait | un grand harem, | mais les visiteurs | du palais | ne savaient pas | où, | et n'avaient | même pas | vraiment | le droit de chercher.

b. Les sultans | satisfaits | depuis peu | avaient | un grand harem, | mais les visiteurs | du palais | ne savaient pas | où, | et n'avaient | même pas | vraiment | le droit de chercher.

c. Le sultan | satisfait | depuis peu | avait | un grand harem, | mais les visiteurs | du palais | ne savaient pas | en trouver | l'emplacement, | et n'avaient | même pas | vraiment | le droit de chercher.

d. Les sultans | satisfaits | depuis peu | avaient | un grand harem, | mais les visiteurs | du palais | ne savaient pas | en trouver | l'emplacement, | et n'avaient | même pas | vraiment | le droit de chercher.

15. a. L'acteur | séduit | à la soirée | a rencontré | une journaliste, | mais les données | prises du portable | ne révèlent pas | où, | bien que | les enquêteurs | aient fait appel | aux techniciens spécialisés.

b. L'actrice | séduite | à la soirée | a rencontré | une journaliste, | mais les

données | prises du portable | ne révèlent pas | où, | bien que | les enquêteurs | aient fait appel | aux techniciens spécialisés.

c. L'acteur | séduit | à la soirée | a rencontré | une journaliste, | mais les données | prises du portable | n'en révèlent pas | l'endroit, | bien que | les enquêteurs | aient fait appel | aux techniciens spécialisés.

d. L'actrice | séduite | à la soirée | a rencontré | une journaliste, | mais les données | prises du portable | n'en révèlent pas | l'endroit, | bien que | les enquêteurs | aient fait appel | aux techniciens spécialisés.

16. a. Le dictionnaire | traduit | de l'anglais | contenait | des familles de mots | en plus, | mais la table | des matières | n'indique pas | lesquelles, | de façon | qu'il serait | extrêmement laborieux | de les identifier.

b. Les dictionnaires | traduits | de l'anglais | contenaient | des familles de mots | en plus, | mais la table | des matières | n'indique pas | lesquelles, | de façon | qu'il serait | extrêmement laborieux | de les identifier.

c. Le dictionnaire | traduit | de l'anglais | contenait | des familles de mots | en plus, | mais la table | des matières | ne les indique pas, | de façon | qu'il serait | extrêmement laborieux | de les identifier.

d. Les dictionnaires | traduits | de l'anglais | contenaient | des familles de mots | en plus, | mais la table | des matières | ne les indique pas, | de façon | qu'il serait | extrêmement laborieux | de les identifier.

TLA stimuli

1. a. Les adolescents ont entendu qu'il y a un pianiste handicapé formidable sur YouTube. | La vidéo | illustre | les touche, | mais il s'avère | curieusement | difficile | de formuler | pourquoi, | sauf que | le triomphe | de la créativité | sur la destinée | est exaltant à regarder.

b. Les adolescents ont entendu qu'il y a un pianiste handicapé formidable sur YouTube. | Les vidéos | illustres | les touchent, | mais il s'avère | curieusement | difficile | de formuler | pourquoi, | sauf que | le triomphe | de la créativité | sur la destinée | est exaltant à regarder.

c. Les adolescents ont entendu qu'il y a un pianiste handicapé formidable sur YouTube. | La vidéo | illustre | les touche, | mais il s'avère | curieusement | difficile | d'en formuler | la raison, | sauf que | le triomphe | de la créativité | sur la destinée | est exaltant à regarder.

d. Les adolescents ont entendu qu'il y a un pianiste handicapé formidable sur YouTube. | Les vidéos | illustres | les touchent, | mais il s'avère | curieusement | difficile | d'en formuler | la raison, | sauf que | le triomphe | de la créativité | sur la destinée | est exaltant à regarder.

2. a. Pour cacher les trous, les ouvriers du chantier attachent de grandes bâches au bateau. | Le montage | fixe | les voile, | mais le patron | sait | qu'on | ne peut | garantir | pour combien de temps, | même si | la visite | d'acheteurs potentiels | se déroule | sans problème.

b. Pour cacher les trous, les ouvriers du chantier attachent de grandes bâches au bateau. | Les montages | fixes | les voilent, | mais le patron | sait | qu'on | ne peut | garantir | pour combien de temps, | même si | la visite | d'acheteurs potentiels | se déroule | sans problème.

c. Pour cacher les trous, les ouvriers du chantier attachent de grandes bâches au bateau. | Le montage | fixe | les voile, | mais le patron | sait | qu'on | ne peut | le garantir, | même si | la visite | d'acheteurs potentiels | se déroule | sans problème.

d. Pour cacher les trous, les ouvriers du chantier attachent de grandes bâches au bateau. | Les montages | fixes | les voilent, | mais le patron | sait | qu'on | ne

peut | le garantir, | même si | la visite | d'acheteurs potentiels | se déroule | sans problème.

3. a. La directrice du groupe découvre que quelques collaborateurs ont détourné d'importantes sommes d'argent. | Le projet | informe | les aide, | mais grâce à | la mauvaise comptabilité, | il n'y a | aucun moyen | de saisir | depuis quand, | incitant | une investigation interne | par | des examinateurs minutieux.
   b. La directrice du groupe découvre que quelques collaborateurs ont détourné d'importantes sommes d'argent. | Les projets | informes | les aident,, | mais grâce à | la mauvaise comptabilité, | il n'y a | aucun moyen | de saisir | depuis quand, | incitant | une investigation interne | par | des examinateurs minutieux.
   c. La directrice du groupe découvre que quelques collaborateurs ont détourné d'importantes sommes d'argent. | Le projet | informe | les aide, | mais grâce à | la mauvaise comptabilité, | il n'y a | aucun moyen | d'en saisir | les coupables, | incitant | une investigation interne | par | des examinateurs minutieux.
   d. La directrice du groupe découvre que quelques collaborateurs ont détourné d'importantes sommes d'argent. | Les projets | informes | les aident, | mais grâce à | la mauvaise comptabilité, | il n'y a | aucun moyen | d'en saisir | les coupables, | incitant | une investigation interne | par | des examinateurs minutieux.
4. a. L'administrateur de la société de vente en ligne détecte des données singulières dans le réseau interne. | Un client | valide | les produit, | mais personne | dans le département informatique | ne peut | déchiffrer | comment, | même si | l'entreprise | qui vend le logiciel utilisé | a déjà été contactée.
   b. L'administrateur de la société de vente en ligne détecte des données singulières dans le réseau interne. | Des clients | valides | les produisent, | mais personne | dans le département informatique | ne peut | déchiffrer | comment, | même si | l'entreprise | qui vend le logiciel utilisé | a déjà été contactée.
   c. L'administrateur de la société de vente en ligne détecte des données singulières dans le réseau interne. | Un client | valide | les produit, | mais personne | dans le département informatique | ne peut | en déchiffrer | l'origine, | même si | l'entreprise | qui vend le logiciel utilisé | a déjà été contactée.
   d. L'administrateur de la société de vente en ligne détecte des données singulières dans le réseau interne. | Des clients | valides | les produisent, | mais personne | dans le département informatique | ne peut | en déchiffrer | l'origine, | même si | l'entreprise | qui vend le logiciel utilisé | a déjà été contactée.
5. a. Les élèves notent que la bataille d'Alésia est mentionnée plusieurs fois dans le livre scolaire. | Le renvoi | explicite | les fait discuter, | mais le professeur | n'a pas le temps | de demander | avec quel résultat, | car il y a | beaucoup | de matières | à traiter | avant l'examen.
   b. Les élèves notent que la bataille d'Alésia est mentionnée plusieurs fois dans le livre scolaire. | Les renvois | explicites | les font discuter, | mais le professeur | n'a pas le temps | de demander | avec quel résultat, | car il y a | beaucoup | de matières | à traiter | avant l'examen.
   c. Les élèves notent que la bataille d'Alésia est mentionnée plusieurs fois dans le livre scolaire. | Le renvoi | explicite | les fait discuter, | mais le professeur | n'a pas le temps | d'en écouter | le résultat, | car il y a | beaucoup | de matières | à traiter | avant l'examen.

d. Les élèves notent que la bataille d'Alésia est mentionnée plusieurs fois dans le livre scolaire. | Les renvois | explicites | les font discuter, | mais le professeur | n'a pas le temps | d'en écouter | le résultat, | car il y a | beaucoup | de matières | à traiter | avant l'examen.

6. a. Un couple brésilien lié au crime organisé est surveillé par le gouvernement argentin depuis longtemps avant de disparaître dans la jungle. | Un agent | lâche | les recherche, | mais à présent | les sources | à l'intérieur du service secret | ne veulent pas | révéler | où, | de manière | à ne pas mettre | en péril | toute l'opération.

   b. Un couple brésilien lié au crime organisé est surveillé par le gouvernement argentin depuis longtemps avant de disparaître dans la jungle. | Des agents | lâches | les recherchent, | mais à présent | les sources | à l'intérieur du service secret | ne veulent pas | révéler | où, | de manière | à ne pas mettre | en péril | toute l'opération.

   c. Un couple brésilien lié au crime organisé est surveillé par le gouvernement argentin depuis longtemps avant de disparaître dans la jungle. | Un agent | lâche | les recherche, | mais à présent | les sources | à l'intérieur du service secret | ne veulent rien | en révéler, | de manière | à ne pas mettre | en péril | toute l'opération.

   d. Un couple brésilien lié au crime organisé est surveillé par le gouvernement argentin depuis longtemps avant de disparaître dans la jungle. | Des agents | lâches | les recherchent, | mais à présent | les sources | à l'intérieur du service secret | ne veulent rien | en révéler, | de manière | à ne pas mettre | en péril | toute l'opération.

7. a. Trois étudiants emprisonnés sont graciés par le nouveau régime comme geste de réconciliation. | La sentence | critique | les juge non-responsables, | mais les survivants | des victimes | de la terreur anti-gouvernementale | ne peuvent pas | comprendre | de quel droit, | protestant | contre | la décision | dans les rues | et sur Internet.

   b. Trois étudiants emprisonnés sont graciés par le nouveau régime comme geste de réconciliation. | Les sentences | critiques | les jugent non-responsables, | mais les survivants | des victimes | de la terreur anti-gouvernementale | ne peuvent pas | comprendre | de quel droit, | protestant | contre | la décision | dans les rues | et sur Internet.

   c. Trois étudiants emprisonnés sont graciés par le nouveau régime comme geste de réconciliation. | La sentence | critique | les juge non-responsables, | mais les survivants | des victimes | de la terreur anti-gouvernementale | ne peuvent pas | en comprendre | la justice, | protestant | contre | la décision | dans les rues | et sur Internet.

   d. Trois étudiants emprisonnés sont graciés par le nouveau régime comme geste de réconciliation. | Les sentences | critiques | les jugent non-responsables, | mais les survivants | des victimes | de la terreur anti-gouvernementale | ne peuvent pas | en comprendre | la justice, | protestant | contre | la décision | dans les rues | et sur Internet.

8. a. Les sondages montrent à maintes reprises que l'extrême droite a pris le dessus, grâce à sa rhétorique contre la société multi-culturelle. | L'opinion | aveugle | les avantage, | mais les commentateurs | plus prévoyants | demandent déjà | pour combien de temps, | d'autant | plus que | l'électorat | d'origine étrangère | augmente | d'année en année.

   b. Les sondages montrent à maintes reprises que l'extrême droite a pris le dessus, grâce à sa rhétorique contre la société multi-culturelle. | Les opinions | aveugles | les avantagent, | mais les commentateurs | plus prévoyants |

demandent déjà | pour combien de temps, | d'autant | plus que | l'électorat | d'origine étrangère | augmente | d'année en année.

c. Les sondages montrent à maintes reprises que l'extrême droite a pris le dessus, grâce à sa rhétorique contre la société multi-culturelle. | L'opinion | aveugle | les avantage, | mais les commentateurs | plus prévoyants | en contestent | la continuité, | d'autant | plus que | l'électorat | d'origine étrangère | augmente | d'année en année.

d. Les sondages montrent à maintes reprises que l'extrême droite a pris le dessus, grâce à sa rhétorique contre la société multi-culturelle. | Les opinions | aveugles | les avantagent, | mais les commentateurs | plus prévoyants | en contestent | la continuité, | d'autant | plus que | l'électorat | d'origine étrangère | augmente | d'année en année.

9. a. Les jeunes sans-abris du campement ne veulent pas rester inertes plus longtemps. | Un camion | détruit | les tente, | mais les anciens | de la petite communauté | sous-estiment | à quel point, | de sorte que | le conflit | avec les ferrailleurs | est déjà clairement | à prévoir.

b. Les jeunes sans-abris du campement ne veulent pas rester inertes plus longtemps. | Des camions | détruits | les tentent, | mais les anciens | de la petite communauté | sous-estiment | à quel point, | de sorte que | le conflit | avec les ferrailleurs | est déjà clairement | à prévoir.

c. Les jeunes sans-abris du campement ne veulent pas rester inertes plus longtemps. | Un camion | détruit | les tente, | mais les anciens | de la petite communauté | en sous-estiment | le danger, | de sorte que | le conflit | avec les ferrailleurs | est déjà clairement | à prévoir.

d. Les jeunes sans-abris du campement ne veulent pas rester inertes plus longtemps. | Des camions | détruits | les tentent, | mais les anciens | de la petite communauté | en sous-estiment | le danger, | de sorte que | le conflit | avec les ferrailleurs | est déjà clairement | à prévoir.

10. a. Les lionceaux couinent dans leur panique. | Un volet | fixe | les piège, | mais les jeunes animaux | ne peuvent pas | comprendre | pourquoi, | jusqu'à | ce que | les braconniers | viennent | les ramasser | pour le transport.

b. Les lionceaux couinent dans leur panique. | Des volets | fixes | les piègent, | mais les jeunes animaux | ne peuvent pas | comprendre | pourquoi, | jusqu'à | ce que | les braconniers | viennent | les ramasser | pour le transport.

c. Les lionceaux couinent dans leur panique. | Un volet | fixe | les piège, | mais les jeunes animaux | ne peuvent pas | en comprendre | le but, | jusqu'à | ce que | les braconniers | viennent | les ramasser | pour le transport.

d. Les lionceaux couinent dans leur panique. | Des volets | fixes | les piègent, | mais les jeunes animaux | ne peuvent pas | en comprendre | le but, | jusqu'à | ce que | les braconniers | viennent | les ramasser | pour le transport.

11. a. Comme les invalides âgés n'ont pas d'argent pour vivre dignement, ils sont forcés d'endurer les défauts de la clinique laissée à l'abandon. | L'infirmier | maudit | les mouche, | mais les vieux | se demandent | souvent | à quelle fin, | étant donné | que la plupart | des occupants | sont proches | de la mort.

b. Comme les invalides âgés n'ont pas d'argent pour vivre dignement, ils sont forcés d'endurer les défauts de la clinique laissée à l'abandon. | L'infirmière | maudite | les mouche, | mais les vieux | se demandent | souvent | à quelle fin, | étant donné | que la plupart | des occupants | sont proches | de la mort.

c. Comme les invalides âgés n'ont pas d'argent pour vivre dignement, ils sont forcés d'endurer les défauts de la clinique laissée à l'abandon. |

d. L'infirmier | maudit | les mouche, | mais les vieux | s'en demandent | souvent | le sens, | étant donné | que la plupart | des occupants | sont proches | de la

mort. Comme les invalides âgés n'ont pas d'argent pour vivre dignement, ils sont forcés d'endurer les défauts de la clinique laissée à l'abandon. | L'infirmière | maudite | les mouche, | mais les vieux | s'en demandent | souvent | le sens, | étant donné | que la plupart | des occupants | sont proches | de la mort.

12. a. Il y a du sang sur le sol du bureau, et les employés savent que c'est le sang du directeur détesté par tout le monde. | La flaque | trouble | les calme, | mais le commissaire | et ses officiers | ne comprennent | pas vraiment | pour quelle raison, | même si | le tué | était décrit | comme un homme | agressif et menaçant.
    b. Il y a du sang sur le sol du bureau, et les employés savent que c'est le sang du directeur détesté par tout le monde. | Les flaques | troubles | les calment, | mais le commissaire | et ses officiers | ne comprennent | pas vraiment | pour quelle raison, | même si | le tué | était décrit | comme un homme | agressif et menaçant.
    c. Il y a du sang sur le sol du bureau, et les employés savent que c'est le sang du directeur détesté par tout le monde. | La flaque | trouble | les calme, | mais le commissaire | et ses officiers | n'en comprennent | pas vraiment | la raison, | même si | le tué | était décrit | comme un homme | agressif et menaçant.
    d. Il y a du sang sur le sol du bureau, et les employés savent que c'est le sang du directeur détesté par tout le monde. | Les flaques | troubles | les calment, | mais le commissaire | et ses officiers | n'en comprennent | pas vraiment | la raison, | même si | le tué | était décrit | comme un homme | agressif et menaçant.
13. a. Pour le stagiaire, les dossiers ne sont que des colonnes de chiffres cabalistiques. | La technicienne | intègre | les signe normalement, | mais il est | toujours | complètement impossible | de prévoir | quand, | donc il ne | lui reste | plus qu'à | attendre silencieusement | sur sa chaise.
    b. Pour le stagiaire, les dossiers ne sont que des colonnes de chiffres cabalistiques. | Les techniciennes | intègres | les signent normalement, | mais il est | toujours | complètement impossible | de prévoir | quand, | donc il ne | lui reste | plus qu'à | attendre silencieusement | sur sa chaise.
    c. Pour le stagiaire, les dossiers ne sont que des colonnes de chiffres cabalistiques. | La technicienne | intègre | les signe normalement, | mais il est | toujours | complètement impossible | d'en prévoir | le délai, | donc il ne | lui reste | plus qu'à | attendre silencieusement | sur sa chaise.
    d. Pour le stagiaire, les dossiers ne sont que des colonnes de chiffres cabalistiques. | Les techniciennes | intègres | les signent normalement, | mais il est | toujours | complètement impossible | d'en prévoir | le délai, | donc il ne | lui reste | plus qu'à | attendre silencieusement | sur sa chaise.
14. a. Les dames âgées sont très satisfaites de la jeune mariée et de ses demoiselles d'honneur. | La robe | sublime | les charme, | mais comme d'habitude | elles invoquent | des raisons très différentes | pour expliquer | pourquoi, | chacune | impatiente | de mettre | son grain de sel.
    b. Les dames âgées sont très satisfaites de la jeune mariée et de ses demoiselles d'honneur. | Les robes | sublimes | les charment, | mais comme d'habitude | elles invoquent | des raisons très différentes | pour expliquer | pourquoi, | chacune | impatiente | de mettre | son grain de sel.
    c. Les dames âgées sont très satisfaites de la jeune mariée et de ses demoiselles d'honneur. | La robe | sublime | les charme, | mais comme d'habitude | elles invoquent | des raisons très différentes | pour expliquer | cette impression, | chacune | impatiente | de mettre | son grain de sel.

d. Les dames âgées sont très satisfaites de la jeune mariée et de ses demoiselles d'honneur. | Les robes | sublimes | les charment, | mais comme d'habitude | elles invoquent | des raisons très différentes | pour expliquer | cette impression, | chacune | impatiente | de mettre | son grain de sel.

15. a. Le marché du quartier est connu pour les grandes côtes de bœuf qui sont livrées durant la nuit. | Le boucher | sale | les tranche, | mais les clients | de la place Colbert | se demandent | incrédulement | quand, | vu que | la viande | est vendue | déjà marinée | et prête à l'emploi | en début de matinée.
    b. Le marché du quartier est connu pour les grandes côtes de bœuf qui sont livrées durant la nuit. | Les bouchers | sales | les tranchent, | mais les clients | de la place Colbert | se demandent | incrédulement | quand, | vu que | la viande | est vendue | déjà marinée | et prête à l'emploi | en début de matinée.
    c. Le marché du quartier est connu pour les grandes côtes de bœuf qui sont livrées durant la nuit. | Le boucher | sale | les tranche, | mais les clients | de la place Colbert | en demandent | incrédulement | la technique, | vu que | la viande | est vendue | déjà marinée | et prête à l'emploi | en début de matinée.
    d. Le marché du quartier est connu pour les grandes côtes de bœuf qui sont livrées durant la nuit. | Les bouchers | sales | les tranchent, | mais les clients | de la place Colbert | en demandent | incrédulement | la technique, | vu que | la viande | est vendue | déjà marinée | et prête à l'emploi | en début de matinée.

16. a. Aujourd'hui, les enfants du vieux couple reçoivent un prix pour leur chanson dans une émission de télévision. | Un modérateur | célèbre | les annonce, | mais les parents | ont des difficultés | à deviner | sur quel canal, | car le journal TV | semble | avoir disparu | une fois de plus.
    b. Aujourd'hui, les enfants du vieux couple reçoivent un prix pour leur chanson dans une émission de télévision. | Des modérateurs | célèbres | les annoncent, | mais les parents | ont des difficultés | à deviner | sur quel canal, | car le journal TV | semble | avoir disparu | une fois de plus.
    c. Aujourd'hui, les enfants du vieux couple reçoivent un prix pour leur chanson dans une émission de télévision. | Un modérateur | célèbre | les annonce, | mais les parents | ont des difficultés | à s'y | concentrer, | car le journal TV | semble | avoir disparu | une fois de plus.
    d. Aujourd'hui, les enfants du vieux couple reçoivent un prix pour leur chanson dans une émission de télévision. | Des modérateurs | célèbres | les annoncent, | mais les parents | ont des difficultés | à s'y | concentrer, | car le journal TV | semble | avoir disparu | une fois de plus.

17. a. Elodie pense que son mari a eu des aventures avec deux de ses assistantes. | Le soupçon | grave | les espace, | mais l'insensible Gabriel | ne veut pas voir | encore | à quel point, | jusqu'à | ce qu'il | soit trop tard | pour sauver | le mariage.
    b. Elodie pense que son mari a eu des aventures avec deux de ses assistantes. | Les soupçons | graves | les espacent, | mais l'insensible Gabriel | ne veut pas voir | encore | à quel point, | jusqu'à | ce qu'il | soit trop tard | pour sauver | le mariage.
    c. Elodie pense que son mari a eu des aventures avec deux de ses assistantes. | Le soupçon | grave | les espace, | mais l'insensible Gabriel | ne veut pas en voir | encore | la sévérité, | jusqu'à | ce qu'il | soit trop tard | pour sauver | le mariage.
    d. Elodie pense que son mari a eu des aventures avec deux de ses assistantes. | Les soupçons | graves | les espacent, | mais l'insensible Gabriel | ne veut pas en voir | encore | la sévérité, | jusqu'à | ce qu'il | soit trop tard | pour sauver |

le mariage.

18. a. Pour la première fois, le chef d'orchestre ose faire jouer aux musiciens une pièce du compositeur hongrois farfelu. | La notation | inverse | les rythme, | mais ni le directeur | ni les spécialistes invités | ne peuvent | expliquer | par quelle magie, | et les instrumentistes | se sont | complètement perdus | durant le concert.
    b. Pour la première fois, le chef d'orchestre ose faire jouer aux musiciens une pièce du compositeur hongrois farfelu. | Les notations | inverses | les rythment, | mais ni le directeur | ni les spécialistes invités | ne peuvent | expliquer | par quelle magie, | et les instrumentistes | se sont | complètement perdus | durant le concert.
    c. Pour la première fois, le chef d'orchestre ose faire jouer aux musiciens une pièce du compositeur hongrois farfelu. | La notation | inverse | les rythme, | mais ni le directeur | ni les spécialistes invités | ne peuvent | l'expliquer, | et les instrumentistes | se sont | complètement perdus | durant le concert.
    d. Pour la première fois, le chef d'orchestre ose faire jouer aux musiciens jouer une pièce du compositeur hongrois farfelu. | Les notations | inverses | les rythment, | mais ni le directeur | ni les spécialistes invités | ne peuvent | l'expliquer, | et les instrumentistes | se sont | complètement perdus | durant le concert.

19. a. Les touristes sont encore traumatisés par la violence de la manifestation et veulent immédiatement retourner à l'hôtel. | Une policière | sauve | les véhicule, | mais il est | un peu inquiétant | que personne ne lui ait indiqué | exactement | où, | vu qu'ils | sont logés | bien loin | en banlieue.
    b. Les touristes sont encore traumatisés par la violence de la manifestation et veulent immédiatement retourner à l'hôtel. | Des policières | sauves | les véhiculent, | mais il est | un peu inquiétant | que personne ne leur ait indiqué | exactement | où, | vu qu'ils | sont logés | bien loin | en banlieue.
    c. Les touristes sont encore traumatisés par la violence de la manifestation et veulent immédiatement retourner à l'hôtel. | Une policière | sauve | les véhicule, | mais il est | un peu inquiétant | que personne ne lui ait indiqué | exactement | la longueur du trajet, | vu qu'ils | sont logés | bien loin | en banlieue.
    d. Les touristes sont encore traumatisés par la violence de la manifestation et veulent immédiatement retourner à l'hôtel. | Des policières | sauves | les véhiculent, | mais il est | un peu inquiétant | que personne ne leur ait indiqué | exactement | la longueur du trajet, | vu qu'ils | sont logés | bien loin | en banlieue.

20. a. Les reliefs sophistiqués sur les colonnes du temple continuent de captiver les archéologues. | Une enjolivure | précise | les orne, | mais les savants | n'arrivent toujours | pas à tomber | d'accord | depuis quand, | car le style | ne semble | correspondre | ni à une époque | ni à une autre.
    b. Les reliefs sophistiqués sur les colonnes du temple continuent de captiver les archéologues. | Des enjolivures | précises | les ornent, | mais les savants | n'arrivent toujours | pas à tomber | d'accord | depuis quand, | car le style | ne semble | correspondre | ni à une époque | ni à une autre.
    c. Les reliefs sophistiqués sur les colonnes du temple continuent de captiver les archéologues. | Une enjolivure | précise | les orne, | mais les savants | n'arrivent toujours | pas à tomber | d'accord | sur leur âge, | car le style | ne semble | correspondre | ni à une époque | ni à une autre.
    d. Les reliefs sophistiqués sur les colonnes du temple continuent de captiver les archéologues. | Des enjolivures | précises | les ornent, | mais les savants |

n'arrivent toujours | pas à tomber | d'accord | sur leur âge, | car le style | ne semble | correspondre | ni à une époque | ni à une autre.

SOI stimuli

1. a. Quels joueurs | du club de foot | a vu | la femme | de mon frère | et où, | si je puis me permettre?
   b. Quels joueurs | du club de foot | ont vu | la femme | de mon frère | et où, | si je puis me permettre?
   c. Quels joueurs | du club de foot | a vu | la femme | de mon frère | récemment, | si je puis me permettre?
   d. Quels joueurs | du club de foot | ont vu | la femme | de mon frère | récemment, | si je puis me permettre?
2. a. Quels avocats | du cabinet | a salué | le juge | du tribunal administratif | et quand, | s'il vous plaît?
   b. Quels avocats | du cabinet | ont salué | le juge | du tribunal administratif | et quand, | s'il vous plaît?
   c. Quels avocats | du cabinet | a salué | le juge | du tribunal administratif | fréquemment, | s'il vous plaît?
   d. Quels avocats | du cabinet | ont salué | le juge | du tribunal administratif | fréquemment, | s'il vous plaît?
3. a. Quels vendeurs | du marché | a dupé | le fils | de ma voisine | et quand, | si vous savez?
   b. Quels vendeurs | du marché | ont dupé | le fils | de ma voisine | et quand, | si vous savez?
   c. Quels vendeurs | du marché | a dupé | le fils | de ma voisine | ce jour-là, | si vous savez?
   d. Quels vendeurs | du marché | ont dupé | le fils | de ma voisine | ce jour-là, | si vous savez?
4. a. Quels acteurs | du théâtre | a insulté | le critique | du quotidien | et comment, | si vous pouvez me dire?
   b. Quels acteurs | du théâtre | ont insulté | le critique | du quotidien | et comment, | si vous pouvez me dire?
   c. Quels acteurs | du théâtre | a insulté | le critique | du quotidien | l'autre jour, | si vous pouvez me dire?
   d. Quels acteurs | du théâtre | ont insulté | le critique | du quotidien | l'autre jour, | si vous pouvez me dire?
5. a. Combien de maîtres | de kung-fu | a vaincu | l'ours | du cirque | et comment, | si je puis me permettre?
   b. Combien de maîtres | de kung-fu | ont vaincu | l'ours | du cirque | et comment, | si je puis me permettre?
   c. Combien de maîtres | de kung-fu | a vaincu | l'ours | du cirque | dans l'histoire, | si je puis me permettre?
   d. Combien de maîtres | de kung-fu | ont vaincu | l'ours | du cirque | dans l'histoire, | si je puis me permettre?
6. a. Combien de voitures | de course | a dépassé | le gros | camion rouge | et quand, | s'il vous plaît?
   b. Combien de voitures | de course | ont dépassé | le gros | camion rouge | et quand, | s'il vous plaît?
   c. Combien de voitures | de course | a dépassé | le gros | camion rouge | en tout, | s'il vous plaît?
   d. Combien de voitures | de course | ont dépassé | le gros | camion rouge | en tout, | s'il vous plaît?

7. a. Quels grimpeurs | de la région | a chassé | l'ermite | du bois | et pourquoi, | si vous savez?
   b. Quels grimpeurs | de la région | ont chassé | l'ermite | du bois | et pourquoi, | si vous savez?
   c. Quels grimpeurs | de la région | a chassé | l'ermite | du bois | le matin, | si vous savez?
   d. Quels grimpeurs | de la région | ont chassé | l'ermite | du bois | le matin, | si vous savez?
8. a. Quels bandits | du désert | a assiégé | l'armée | des Croisés | et quand, | si vous pouvez me dire?
   b. Quels bandits | du désert | ont assiégé | l'armée | des Croisés | et quand, | si vous pouvez me dire?
   c. Quels bandits | du désert | a assiégé | l'armée | des Croisés | en 1267, | si vous pouvez me dire?
   d. Quels bandits | du désert | ont assiégé | l'armée | des Croisés | en 1267, | si vous pouvez me dire?
9. a. Quels fidèles | de l'hindouisme | a converti | le pasteur | de l'église paroissiale | et comment, | si je puis me permettre?
   b. Quels fidèles | de l'hindouisme | ont converti | le pasteur | de l'église paroissiale | et comment, | si je puis me permettre?
   c. Quels fidèles | de l'hindouisme | a converti | le pasteur | de l'église paroissiale | au final, | si je puis me permettre?
   d. Quels fidèles | de l'hindouisme | ont converti | le pasteur | de l'église paroissiale | au final, | si je puis me permettre?
10. a. Quelles journalistes | de la chaîne | a soudoyé | le président | du parti | et quand, | s'il vous plaît?
    b. Quelles journalistes | de la chaîne | ont soudoyé | le président | du parti | et quand, | s'il vous plaît?
    c. Quelles journalistes | de la chaîne | a soudoyé | le président | du parti | l'année dernière, | s'il vous plaît?
    d. Quelles journalistes | de la chaîne | ont soudoyé | le président | du parti | l'année dernière, | s'il vous plaît?
11. a. Quelle chanteuse | d'opéra | ont contacté | les musiciens | du groupe | et pourquoi, | si vous savez?
    b. Quelle chanteuse | d'opéra | a contacté | les musiciens | du groupe | et pourquoi, | si vous savez?
    c. Quelle chanteuse | d'opéra | ont contacté | les musiciens | du groupe | en cachette, | si vous savez?
    d. Quelle chanteuse | d'opéra | a contacté | les musiciens | du groupe | en cachette, | si vous savez?
12. a. Quel chimiste | du groupe pharmaceutique | ont empoisonné | les collègues | du département | et comment, | si vous pouvez me dire?
    b. Quel chimiste | du groupe pharmaceutique | a empoisonné | les collègues | du département | et comment, | si vous pouvez me dire?
    c. Quel chimiste | du groupe pharmaceutique | ont empoisonné | les collègues | du département | par vengeance, | si vous pouvez me dire?
    d. Quel chimiste | du groupe pharmaceutique | a empoisonné | les collègues | du département | par vengeance, | si vous pouvez me dire?
13. a. Quel escrimeur | de l'équipe nationale | ont salué | les adversaires | du concours | et quand, | si je puis me permettre?
    b. Quel escrimeur | de l'équipe nationale | a salué | les adversaires | du concours | et quand, | si je puis me permettre?

c. Quel escrimeur | de l'équipe nationale | ont salué | les adversaires | du concours | avant la lutte, | si je puis me permettre?
d. Quel escrimeur | de l'équipe nationale | a salué | les adversaires | du concours | avant la lutte, | si je puis me permettre?

14. a. Quel héros | de la gâchette | ont inhumé | les compagnons | de bordée | et où, | s'il vous plaît?
b. Quel héros | de la gâchette | a inhumé | les compagnons | de bordée | et où, | s'il vous plaît?
c. Quel héros | de la gâchette | ont inhumé | les compagnons | de bordée | dans le désert, | s'il vous plaît?
d. Quel héros | de la gâchette | a inhumé | les compagnons | de bordée | dans le désert, | s'il vous plaît?

15. a. Quel auteur | de romans policiers | ont consulté | les enquêteurs | du commissariat | et pourquoi, | si vous savez?
b. Quel auteur | de romans policiers | a consulté | les enquêteurs | du commissariat | et pourquoi, | si vous savez?
c. Quel auteur | de romans policiers | ont consulté | les enquêteurs | du commissariat | par téléphone, | si vous savez?
d. Quel auteur | de romans policiers | a consulté | les enquêteurs | du commissariat | par téléphone, | si vous savez?

16. a. Quel fonctionnaire | de l'Etat | ont protégé | les magistrats | du parquet | et quand, | si vous pouvez me dire?
b. Quel fonctionnaire | de l'Etat | a protégé | les magistrats | du parquet | et quand, | si vous pouvez me dire?
c. Quel fonctionnaire | de l'Etat | ont protégé | les magistrats | du parquet | en août dernier, | si vous pouvez me dire?
d. Quel fonctionnaire | de l'Etat | a protégé | les magistrats | du parquet | en août dernier, | si vous pouvez me dire?

17. a. Quelle dompteuse | de lion | ont embauché | les cracheurs | de feu | et pourquoi, | si je puis me permettre?
b. Quelle dompteuse | de lion | a embauché | les cracheurs | de feu | et pourquoi, | si je puis me permettre?
c. Quelle dompteuse | de lion | ont embauché | les cracheurs | de feu | illicitement, | si je puis me permettre?
d. Quelle dompteuse | de lion | a embauché | les cracheurs | de feu | illicitement, | si je puis me permettre?

18. a. Quel roi | de l'Angleterre | ont raillé | les ministres | de la Couronne | et pourquoi, | s'il vous plaît?
b. Quel roi | de l'Angleterre | a raillé | les ministres | de la Couronne | et pourquoi, | s'il vous plaît?
c. Quel roi | de l'Angleterre | ont raillé | les ministres | de la Couronne | sans cesse, | s'il vous plaît?
d. Quel roi | de l'Angleterre | a raillé | les ministres | de la Couronne | sans cesse, | s'il vous plaît?

19. a. Quel assassin | de la mafia | ont observé | les agents infiltrés | du service secret | et quand, | si vous savez?
b. Quel assassin | de la mafia | a observé | les agents infiltrés | du service secret | et quand, | si vous savez?
c. Quel assassin | de la mafia | ont observé | les agents infiltrés | du service secret | particulièrement, | si vous savez?
d. Quel assassin | de la mafia | a observé | les agents infiltrés | du service secret | particulièrement, | si vous savez?

20. a. Quel explorateur | de l'institut ethnologique | ont caché | les autochtones | du village | et où, | si vous pouvez me dire?
    b. Quel explorateur | de l'institut ethnologique | a caché | les autochtones | du village | et où, | si vous pouvez me dire?
    c. Quel explorateur | de l'institut ethnologique | ont caché | les autochtones | du village | quelque part, | si vous pouvez me dire?
    d. Quel explorateur | de l'institut ethnologique | a caché | les autochtones | du village | quelque part, | si vous pouvez me dire?

Appendix II – Items used in Experiment 2

Vertical bars indicate boundaries between regions of interest.

1. a. Ein Spieler des Vereins | hatte | die aufdringlichen Fans | nach dem Auswärtsspiel | grob beleidigt, | aber | der Trainer | konnte | nicht | verstehen, | warum, | sodass | er | nur | enttäuscht | den Kopf schüttelte.
   b. Einen Spieler des Vereins | hatten | die aufdringlichen Fans | nach dem Auswärtsspiel | grob beleidigt, | aber | der Trainer | konnte | nicht | verstehen, | warum, | sodass | er | nur | enttäuscht | den Kopf schüttelte.
   c. Eine Spielerin des Vereins | hatte | die aufdringlichen Fans | nach dem Auswärtsspiel | grob beleidigt, | aber | der Trainer | konnte | nicht | verstehen, | warum, | sodass | er | nur | enttäuscht | den Kopf schüttelte.
   d. Eine Spielerin des Vereins | hatten | die aufdringlichen Fans | nach dem Auswärtsspiel | grob beleidigt, | aber | der Trainer | konnte | nicht | verstehen, | warum, | sodass | er | nur | enttäuscht | den Kopf schüttelte.
2. a. Ein Schüler des Schachmeisters | hatte | die Schiedsrichter | während des Turniers | sehr genau beobachtet, | aber | der aufmerksame Zuschauer | fragte | sich | noch immer, | warum, | als | er | am Abend | endlich | nach Hause kam.
   b. Einen Schüler des Schachmeisters | hatten | die Schiedsrichter | während des Turniers | sehr genau beobachtet, | aber | der aufmerksame Zuschauer | fragte | sich | noch immer, | warum, | als | er | am Abend | endlich | nach Hause kam.
   c. Eine Schülerin des Schachmeisters | hatte | die Schiedsrichter | während des Turniers | sehr genau beobachtet, | aber | der aufmerksame Zuschauer | fragte | sich | noch immer, | warum, | als | er | am Abend | endlich | nach Hause kam.
   d. Eine Schülerin des Schachmeisters | hatten | die Schiedsrichter | während des Turniers | sehr genau beobachtet, | aber | der aufmerksame Zuschauer | fragte | sich | noch immer, | warum, | als | er | am Abend | endlich | nach Hause kam.
3. a. Ein Gönner des Künstlers | hatte | die etwas seltsamen Verwandten | zu Anfang | des Mordes verdächtigt, | aber | aus den Tagebüchern | geht | nicht | hervor, | warum, | zumal | es | sich | relativ eindeutig | um Suizid handelte.
   b. Einen Gönner des Künstlers | hatten | die etwas seltsamen Verwandten | zu Anfang | des Mordes verdächtigt, | aber | aus den Tagebüchern | geht | nicht | hervor, | warum, | zumal | es | sich | relativ eindeutig | um Suizid handelte.
   c. Eine Gönnerin des Künstlers | hatte | die etwas seltsamen Verwandten | zu Anfang | des Mordes verdächtigt, | aber | aus den Tagebüchern | geht | nicht | hervor, | warum, | zumal | es | sich | relativ eindeutig | um Suizid handelte.
   d. Eine Gönnerin des Künstlers | hatten | die etwas seltsamen Verwandten | zu Anfang | des Mordes verdächtigt, | aber | aus den Tagebüchern | geht | nicht | hervor, | warum, | zumal | es | sich | relativ eindeutig | um Suizid handelte.

4. a. Ein Sympathisant der Opposition | hatte | die Rebellen | laut einem Bericht | maßgeblich unterstützt, | aber | die Regierung | konnte | nicht | nachweisen, | wie, | so sehr | sich | die Untersuchungskommission | auch | bemühte.
   b. Einen Sympathisanten der Opposition | hatten | die Rebellen | laut einem Bericht | maßgeblich unterstützt, | aber | die Regierung | konnte | nicht | nachweisen, | wie, | so sehr | sich | die Untersuchungskommission | auch | bemühte.
   c. Eine Sympathisantin der Opposition | hatte | die Rebellen | laut einem Bericht | maßgeblich unterstützt, | aber | die Regierung | konnte | nicht | nachweisen, | wie, | so sehr | sich | die Untersuchungskommission | auch | bemühte.
   d. Eine Sympathisantin der Opposition | hatten | die Rebellen | laut einem Bericht | maßgeblich unterstützt, | aber | die Regierung | konnte | nicht | nachweisen, | wie, | so sehr | sich | die Untersuchungskommission | auch | bemühte.
5. a. Ein Sprecher des Pharmakonzerns | hatte | die Sportler | nach Angaben der Presse | persönlich getroffen, | aber | die Quelle | konnte | nicht | mitteilen, | wo, | sodass | die Geschichte | den meisten Lesern | wahrscheinlich | nicht sehr glaubwürdig erschien.
   b. Einen Sprecher des Pharmakonzerns | hatten | die Sportler | nach Angaben der Presse | persönlich getroffen, | aber | die Quelle | konnte | nicht | mitteilen, | wo, | sodass | die Geschichte | den meisten Lesern | wahrscheinlich | nicht sehr glaubwürdig erschien.
   c. Eine Sprecherin des Pharmakonzerns | hatte | die Sportler | nach Angaben der Presse | persönlich getroffen, | aber | die Quelle | konnte | nicht | mitteilen, | wo, | sodass | die Geschichte | den meisten Lesern | wahrscheinlich | nicht sehr glaubwürdig erschien.
   d. Eine Sprecherin des Pharmakonzerns | hatten | die Sportler | nach Angaben der Presse | persönlich getroffen, | aber | die Quelle | konnte | nicht | mitteilen, | wo, | sodass | die Geschichte | den meisten Lesern | wahrscheinlich | nicht sehr glaubwürdig erschien.
6. a. Ein Berater des Präsidenten | hatte | die Ermittler | offensichtlich | mit Erfolg getäuscht, | aber | man | fand | nie | heraus, | wie, | denn | es | galt | nach wie vor | die höchste Geheimhaltungsstufe.
   b. Einen Berater des Präsidenten | hatten | die Ermittler | offensichtlich | mit Erfolg getäuscht, | aber | man | fand | nie | heraus, | wie, | denn | es | galt | nach wie vor | die höchste Geheimhaltungsstufe.
   c. Eine Beraterin des Präsidenten | hatte | die Ermittler | offensichtlich | mit Erfolg getäuscht, | aber | man | fand | nie | heraus, | wie, | denn | es | galt | nach wie vor | die höchste Geheimhaltungsstufe.
   d. Eine Beraterin des Präsidenten | hatten | die Ermittler | offensichtlich | mit Erfolg getäuscht, | aber | man | fand | nie | heraus, | wie, | denn | es | galt | nach wie vor | die höchste Geheimhaltungsstufe.
7. a. Ein Kellner des Lokals | hatte | die Stammgäste | über das geplante Skatturnier | ausgefragt, | aber | der Wirt | konnte | nicht | sagen, | warum, | da | er | offenbar | an jenem Abend | sehr beschäftigt gewesen war.
   b. Einen Kellner des Lokals | hatten | die Stammgäste | über das geplante Skatturnier | ausgefragt, | aber | der Wirt | konnte | nicht | sagen, | warum, | da | er | offenbar | an jenem Abend | sehr beschäftigt gewesen war.
   c. Eine Kellnerin des Lokals | hatte | die Stammgäste | über das geplante Skatturnier | ausgefragt, | aber | der Wirt | konnte | nicht | sagen, | warum, | da | er | offenbar | an jenem Abend | sehr beschäftigt gewesen war.

d. Eine Kellnerin des Lokals | hatten | die Stammgäste | über das geplante Skatturnier | ausgefragt, | aber | der Wirt | konnte | nicht | sagen, | warum, | da | er | offenbar | an jenem Abend | sehr beschäftigt gewesen war.

8. a. Ein Soldat der gegnerischen Streitkräfte | hatte | die ausgesandten Kundschafter | offenbar | in die Irre geführt, | aber | der Befehlshaber | begriff | einfach | nicht, | wie, | obwohl | ihm | die Finte | mehrmals | erklärt worden war.
   b. Einen Soldaten der gegnerischen Streitkräfte | hatten | die ausgesandten Kundschafter | offenbar | in die Irre geführt, | aber | der Befehlshaber | begriff | einfach | nicht, | wie, | obwohl | ihm | die Finte | mehrmals | erklärt worden war.
   c. Eine Soldatin der gegnerischen Streitkräfte | hatte | die ausgesandten Kundschafter | offenbar | in die Irre geführt, | aber | der Befehlshaber | begriff | einfach | nicht, | wie, | obwohl | ihm | die Finte | mehrmals | erklärt worden war.
   d. Eine Soldatin der gegnerischen Streitkräfte | hatten | die ausgesandten Kundschafter | offenbar | in die Irre geführt, | aber | der Befehlshaber | begriff | einfach | nicht, | wie, | obwohl | ihm | die Finte | mehrmals | erklärt worden war.

9. a. Ein Gegner des umstrittenen Staudammprojekts | hatte | die Planer | schließlich | doch noch überzeugt, | aber | es | herrscht | Stillschweigen | darüber, | wie, | weil | niemand | sich | dem Verdacht der Bestechlichkeit | aussetzen will.
   b. Einen Gegner des umstrittenen Staudammprojekts | hatten | die Planer | schließlich | doch noch überzeugt, | aber | es | herrscht | Stillschweigen | darüber, | wie, | weil | niemand | sich | dem Verdacht der Bestechlichkeit | aussetzen will.
   c. Eine Gegnerin des umstrittenen Staudammprojekts | hatte | die Planer | schließlich | doch noch überzeugt, | aber | es | herrscht | Stillschweigen | darüber, | wie, | weil | niemand | sich | dem Verdacht der Bestechlichkeit | aussetzen will.
   d. Eine Gegnerin des umstrittenen Staudammprojekts | hatten | die Planer | schließlich | doch noch überzeugt, | aber | es | herrscht | Stillschweigen | darüber, | wie, | weil | niemand | sich | dem Verdacht der Bestechlichkeit | aussetzen will.

10. a. Ein Befürworter der Steuerreform | hatte | die Leiter | der betroffenen Behörden | wiederholt | verbal angegriffen, | aber | es | bleibt | völlig | im Dunkeln, | weshalb, | da | das Wortgefecht | von beiden Seiten | überaus unsachlich | geführt wurde.
   b. Einen Befürworter der Steuerreform | hatten | die Leiter | der betroffenen Behörden | wiederholt | verbal angegriffen, | aber | es | bleibt | völlig | im Dunkeln, | weshalb, | da | das Wortgefecht | von beiden Seiten | überaus unsachlich | geführt wurde.
   c. Eine Befürworterin der Steuerreform | hatte | die Leiter | der betroffenen Behörden | wiederholt | verbal angegriffen, | aber | es | bleibt | völlig | im Dunkeln, | weshalb, | da | das Wortgefecht | von beiden Seiten | überaus unsachlich | geführt wurde.
   d. Eine Befürworterin der Steuerreform | hatten | die Leiter | der betroffenen Behörden | wiederholt | verbal angegriffen, | aber | es | bleibt | völlig | im Dunkeln, | weshalb, | da | das Wortgefecht | von beiden Seiten | überaus unsachlich | geführt wurde.

11. a. Ein Vertrauter des Bürgermeisters | hatte | die Ratsmitglieder | kurz vor der

Wahl | auffallend häufig angerufen, | aber | heute | weiß | niemand | mehr, | warum, | wie | eine Zeitung | kürzlich | in einem Kommentar | schrieb.

b. Einen Vertrauten des Bürgermeisters | hatten | die Ratsmitglieder | kurz vor der Wahl | auffallend häufig angerufen, | aber | heute | weiß | niemand | mehr, | warum, | wie | eine Zeitung | kürzlich | in einem Kommentar | schrieb.

c. Eine Vertraute des Bürgermeisters | hatte | die Ratsmitglieder | kurz vor der Wahl | auffallend häufig angerufen, | aber | heute | weiß | niemand | mehr, | warum, | wie | eine Zeitung | kürzlich | in einem Kommentar | schrieb.

d. Eine Vertraute des Bürgermeisters | hatten | die Ratsmitglieder | kurz vor der Wahl | auffallend häufig angerufen, | aber | heute | weiß | niemand | mehr, | warum, | wie | eine Zeitung | kürzlich | in einem Kommentar | schrieb.

12. a. Ein Sanitäter des Rettungsteams | hatte | die Feuerwehrleute | nachdrücklich | um Hilfe gebeten, | aber | man | verstand | später | nicht, | warum, | bis | schließlich | Bildmaterial vom Unglücksort | das Ausmaß der Verwüstung | verständlich machte.

b. Einen Sanitäter des Rettungsteams | hatten | die Feuerwehrleute | nachdrücklich | um Hilfe gebeten, | aber | man | verstand | später | nicht, | warum, | bis | schließlich | Bildmaterial vom Unglücksort | das Ausmaß der Verwüstung | verständlich machte.

c. Eine Sanitäterin des Rettungsteams | hatte | die Feuerwehrleute | nachdrücklich | um Hilfe gebeten, | aber | man | verstand | später | nicht, | warum, | bis | schließlich | Bildmaterial vom Unglücksort | das Ausmaß der Verwüstung | verständlich machte.

d. Eine Sanitäterin des Rettungsteams | hatten | die Feuerwehrleute | nachdrücklich | um Hilfe gebeten, | aber | man | verstand | später | nicht, | warum, | bis | schließlich | Bildmaterial vom Unglücksort | das Ausmaß der Verwüstung | verständlich machte.

13. a. Ein Abgeordneter der Landtagsfraktion | hatte | die Finanzbeamten | in einem offenen Brief | gemaßregelt, | aber | fünfzig Jahre später | erscheint | es | unverständlich, | weshalb, | da | aus heutiger Sicht | wohl | kein Fehlverhalten | vorlag.

b. Einen Abgeordneten der Landtagsfraktion | hatten | die Finanzbeamten | in einem offenen Brief | gemaßregelt, | aber | fünfzig Jahre später | erscheint | es | unverständlich, | weshalb, | da | aus heutiger Sicht | wohl | kein Fehlverhalten | vorlag.

c. Eine Abgeordnete der Landtagsfraktion | hatte | die Finanzbeamten | in einem offenen Brief | gemaßregelt, | aber | fünfzig Jahre später | erscheint | es | unverständlich, | weshalb, | da | aus heutiger Sicht | wohl | kein Fehlverhalten | vorlag.

d. Eine Abgeordnete der Landtagsfraktion | hatten | die Finanzbeamten | in einem offenen Brief | gemaßregelt, | aber | fünfzig Jahre später | erscheint | es | unverständlich, | weshalb, | da | aus heutiger Sicht | wohl | kein Fehlverhalten | vorlag.

14. a. Ein Mathematiker mit Programmierkenntnissen | hatte | die Seitenbetreiber | über die Sicherheitslücke | informiert, | aber | der Staatsanwalt | wollte | genau | wissen, | wann, | da | dies | für den Tathergang | womöglich | äußerst entscheidend war.

b. Einen Mathematiker mit Programmierkenntnissen | hatten | die Seitenbetreiber | über die Sicherheitslücke | informiert, | aber | der Staatsanwalt | wollte | genau | wissen, | wann, | da | dies | für den Tathergang | womöglich | äußerst entscheidend war.

c. Eine Mathematikerin mit Programmierkenntnissen | hatte | die

Seitenbetreiber | über die Sicherheitslücke | informiert, | aber | der Staatsanwalt | wollte | genau | wissen, | wann, | da | dies | für den Tathergang | womöglich | äußerst entscheidend war. | wann, da dies für den Tathergang womöglich äußerst entscheidend war.

d. Eine Mathematikerin mit Programmierkenntnissen | hatten | die Seitenbetreiber | über die Sicherheitslücke | informiert, | aber | der Staatsanwalt | wollte | genau | wissen, | wann, | da | dies | für den Tathergang | womöglich | äußerst entscheidend war.

15. a. Ein Schwimmer mit zwei Beinprothesen | hatte | die Komiteemitglieder | bezüglich der geplanten Werbekampagne | kontaktiert, | aber | es | bleibt | äußerst schleierhaft, | wann, | zumal | das Schriftstück | angeblich | zwischenzeitlich | verloren gegangen ist.

b. Einen Schwimmer mit zwei Beinprothesen | hatten | die Komiteemitglieder | bezüglich der geplanten Werbekampagne | kontaktiert, | aber | es | bleibt | äußerst schleierhaft, | wann, | zumal | das Schriftstück | angeblich | zwischenzeitlich | verloren gegangen ist.

c. Eine Schwimmerin mit zwei Beinprothesen | hatte | die Komiteemitglieder | bezüglich der geplanten Werbekampagne | kontaktiert, | aber | es | bleibt | äußerst schleierhaft, | wann, | zumal | das Schriftstück | angeblich | zwischenzeitlich | verloren gegangen ist.

d. Eine Schwimmerin mit zwei Beinprothesen | hatten | die Komiteemitglieder | bezüglich der geplanten Werbekampagne | kontaktiert, | aber | es | bleibt | äußerst schleierhaft, | wann, | zumal | das Schriftstück | angeblich | zwischenzeitlich | verloren gegangen ist.

16. a. Ein Student mit außergewöhnlichen Leistungen | hatte | die Professoren | laut Stellungnahme des Instituts | tatkräftig unterstützt, | aber | es | war | nicht | zu erfahren, | wobei, | da | der Projektverantwortliche | nicht | für Nachfragen | zu erreichen ist.

b. Einen Studenten mit außergewöhnlichen Leistungen | hatten | die Professoren | laut Stellungnahme des Instituts | tatkräftig unterstützt, | aber | es | war | nicht | zu erfahren, | wobei, | da | der Projektverantwortliche | nicht | für Nachfragen | zu erreichen ist.

c. Eine Studentin mit außergewöhnlichen Leistungen | hatte | die Professoren | laut Stellungnahme des Instituts | tatkräftig unterstützt, | aber | es | war | nicht | zu erfahren, | wobei, | da | der Projektverantwortliche | nicht | für Nachfragen | zu erreichen ist.

d. Eine Studentin mit außergewöhnlichen Leistungen | hatten | die Professoren | laut Stellungnahme des Instituts | tatkräftig unterstützt, | aber | es | war | nicht | zu erfahren, | wobei, | da | der Projektverantwortliche | nicht | für Nachfragen | zu erreichen ist.

17. a. Ein Autor aus Bolivien | hatte | die vier Literaturwissenschaftler | in einem 2500-Seiten-Werk | zitiert, | aber | noch | kann | niemand | sagen, | wo, | da | der Text | bislang | seltsamerweise | verschollen blieb.

b. Einen Autor aus Bolivien | hatten | die vier Literaturwissenschaftler | in einem 2500-Seiten-Werk | zitiert, | aber | noch | kann | niemand | sagen, | wo, | da | der Text | bislang | seltsamerweise | verschollen blieb.

c. Eine Autorin aus Bolivien | hatte | die vier Literaturwissenschaftler | in einem 2500-Seiten-Werk | zitiert, | aber | noch | kann | niemand | sagen, | wo, | da | der Text | bislang | seltsamerweise | verschollen blieb.

d. Eine Autorin aus Bolivien | hatten | die vier Literaturwissenschaftler | in einem 2500-Seiten-Werk | zitiert, | aber | noch | kann | niemand | sagen, | wo, | da | der Text | bislang | seltsamerweise | verschollen blieb.

18. a. Ein Korrespondent des erfolgreichen Nachrichtensenders | hatte | die Kollegen | vor laufender Kamera | schlechtgemacht, | aber | in einem Gespräch | konnte | nicht | festgestellt werden, | weshalb, | sodass | der Konflikt | trotz aller Entschuldigungen | ohne Zweifel | weiterhin bestehen blieb.
    b. Einen Korrespondenten des erfolgreichen Nachrichtensenders | hatten | die Kollegen | vor laufender Kamera | schlechtgemacht, | aber | in einem Gespräch | konnte | nicht | festgestellt werden, | weshalb, | sodass | der Konflikt | trotz aller Entschuldigungen | ohne Zweifel | weiterhin bestehen blieb.
    c. Eine Korrespondentin des erfolgreichen Nachrichtensenders | hatte | die Kollegen | vor laufender Kamera | schlechtgemacht, | aber | in einem Gespräch | konnte | nicht | festgestellt werden, | weshalb, | sodass | der Konflikt | trotz aller Entschuldigungen | ohne Zweifel | weiterhin bestehen blieb.
    d. Eine Korrespondentin des erfolgreichen Nachrichtensenders | hatten | die Kollegen | vor laufender Kamera | schlechtgemacht, | aber | in einem Gespräch | konnte | nicht | festgestellt werden, | weshalb, | sodass | der Konflikt | trotz aller Entschuldigungen | ohne Zweifel | weiterhin bestehen blieb.
19. a. Ein Violinist des Nationalorchesters | hatte | die Konzertbesucher | während der halbstündigen Pause | heimlich fotografiert, | aber | der Beitrag | verriet | leider | nicht, | weshalb, | sondern | befasste | sich | eher | mit der Bildqualität.
    b. Einen Violinisten des Nationalorchesters | hatten | die Konzertbesucher | während der halbstündigen Pause | heimlich fotografiert, | aber | der Beitrag | verriet | leider | nicht, | weshalb, | sondern | befasste | sich | eher | mit der Bildqualität.
    c. Eine Violinistin des Nationalorchesters | hatte | die Konzertbesucher | während der halbstündigen Pause | heimlich fotografiert, | aber | der Beitrag | verriet | leider | nicht, | weshalb, | sondern | befasste | sich | eher | mit der Bildqualität.
    d. Eine Violinistin des Nationalorchesters | hatten | die Konzertbesucher | während der halbstündigen Pause | heimlich fotografiert, | aber | der Beitrag | verriet | leider | nicht, | weshalb, | sondern | befasste | sich | eher | mit der Bildqualität.
20. a. Ein Straßenhund mit schwarzem Fell | hatte | die Kinder | bis an den Rand des Dorfes | verfolgt, | aber | niemand | konnte | sich | erklären, | weshalb, | zumal | das Tier | sich | normalerweise | vor Menschen versteckte.
    b. Einen Straßenhund mit schwarzem Fell | hatten | die Kinder | bis an den Rand des Dorfes | verfolgt, | aber | niemand | konnte | sich | erklären, | weshalb, | zumal | das Tier | sich | normalerweise | vor Menschen versteckte.
    c. Eine Straßenhündin mit schwarzem Fell | hatte | die Kinder | bis an den Rand des Dorfes | verfolgt, | aber | niemand | konnte | sich | erklären, | weshalb, | zumal | das Tier | sich | normalerweise | vor Menschen versteckte.
    d. Eine Straßenhündin mit schwarzem Fell | hatten | die Kinder | bis an den Rand des Dorfes | verfolgt, | aber | niemand | konnte | sich | erklären, | weshalb, | zumal | das Tier | sich | normalerweise | vor Menschen versteckte.
21. a. Ein Teenager ohne Schulabschluss | hatte | die Talentsucher | in der Bewerbungsphase | angeschrieben, | aber | der Programmverantwortliche | fragte | sich | ernsthaft, | wozu, | denn | bemerkenswerte Fähigkeiten | wurden | an keiner Stelle | erwähnt.
    b. Einen Teenager ohne Schulabschluss | hatten | die Talentsucher | in der

Bewerbungsphase | angeschrieben, | aber | der Programmverantwortliche | fragte | sich | ernsthaft, | wozu, | denn | bemerkenswerte Fähigkeiten | wurden | an keiner Stelle | erwähnt.

c. Eine Teenagerin ohne Schulabschluss | hatte | die Talentsucher | in der Bewerbungsphase | angeschrieben, | aber | der Programmverantwortliche | fragte | sich | ernsthaft, | wozu, | denn | bemerkenswerte Fähigkeiten | wurden | an keiner Stelle | erwähnt.

d. Eine Teenagerin ohne Schulabschluss | hatten | die Talentsucher | in der Bewerbungsphase | angeschrieben, | aber | der Programmverantwortliche | fragte | sich | ernsthaft, | wozu, | denn | bemerkenswerte Fähigkeiten | wurden | an keiner Stelle | erwähnt.

22. a. Ein Vertreter der Gewerkschaft | hatte | die anwesenden Minister | während der Sitzung | scharf attackiert, | aber | der gesprächige Parlamentarier | wusste | selbst | nicht, | warum, | denn | er | war | nicht | dabei gewesen.

b. Einen Vertreter der Gewerkschaft | hatten | die anwesenden Minister | während der Sitzung | scharf attackiert, | aber | der gesprächige Parlamentarier | wusste | selbst | nicht, | warum, | denn | er | war | nicht | dabei gewesen.

c. Eine Vertreterin der Gewerkschaft | hatte | die anwesenden Minister | während der Sitzung | scharf attackiert, | aber | der gesprächige Parlamentarier | wusste | selbst | nicht, | warum, | denn | er | war | nicht | dabei gewesen.

d. Eine Vertreterin der Gewerkschaft | hatten | die anwesenden Minister | während der Sitzung | scharf attackiert, | aber | der gesprächige Parlamentarier | wusste | selbst | nicht, | warum, | denn | er | war | nicht | dabei gewesen.

23. a. Ein Patient mit unklaren Symptomen | hatte | die Krankenschwestern | dem behandelnden Arzt zufolge | mehrfach angeschrien, | aber | es | war | nicht | zu ergründen, | wieso, | obwohl | seitdem | schon | mehrere Gespräche | geführt wurden.

b. Einen Patienten mit unklaren Symptomen | hatten | die Krankenschwestern | dem behandelnden Arzt zufolge | mehrfach angeschrien, | aber | es | war | nicht | zu ergründen, | wieso, | obwohl | seitdem | schon | mehrere Gespräche | geführt wurden.

c. Eine Patientin mit unklaren Symptomen | hatte | die Krankenschwestern | dem behandelnden Arzt zufolge | mehrfach angeschrien, | aber | es | war | nicht | zu ergründen, | wieso, | obwohl | seitdem | schon | mehrere Gespräche | geführt wurden.

d. Eine Patientin mit unklaren Symptomen | hatten | die Krankenschwestern | dem behandelnden Arzt zufolge | mehrfach angeschrien, | aber | es | war | nicht | zu ergründen, | wieso, | obwohl | seitdem | schon | mehrere Gespräche | geführt wurden.

24. a. Ein Biologe mit Doktortitel | hatte | die Naturschützer | auf einer Fachkonferenz | äußerst heftig kritisiert, | aber | die anderen Teilnehmer | erinnerten | sich | nicht, | wieso, | zumal | die Diskussion | offenbar | abseits des Podiums | stattfand.

b. Einen Biologen mit Doktortitel | hatten | die Naturschützer | auf einer Fachkonferenz | äußerst heftig kritisiert, | aber | die anderen Teilnehmer | erinnerten | sich | nicht, | wieso, | zumal | die Diskussion | offenbar | abseits des Podiums | stattfand.

c. Eine Biologin mit Doktortitel | hatte | die Naturschützer | auf einer Fachkonferenz | äußerst heftig kritisiert, | aber | die anderen Teilnehmer |

erinnerten | sich | nicht, | wieso, | zumal | die Diskussion | offenbar | abseits des Podiums | stattfand.

d. Eine Biologin mit Doktortitel | hatten | die Naturschützer | auf einer Fachkonferenz | äußerst heftig kritisiert, | aber | die anderen Teilnehmer | erinnerten | sich | nicht, | wieso, | zumal | die Diskussion | offenbar | abseits des Podiums | stattfand.

25. a. Ein Sachverständiger aus Osteuropa | hatte | die Investoren | in der Planungsphase | eigenständig hinzugezogen, | aber | im Nachhinein | fragte | sich | so mancher Gutachter, | wieso, | da | das Ergebnis | augenscheinlich | nicht | verbessert wurde.

b. Einen Sachverständigen aus Osteuropa | hatten | die Investoren | in der Planungsphase | eigenständig hinzugezogen, | aber | im Nachhinein | fragte | sich | so mancher Gutachter, | wieso, | da | das Ergebnis | augenscheinlich | nicht | verbessert wurde.

c. Eine Sachverständige aus Osteuropa | hatte | die Investoren | in der Planungsphase | eigenständig hinzugezogen, | aber | im Nachhinein | fragte | sich | so mancher Gutachter, | wieso, | da | das Ergebnis | augenscheinlich | nicht | verbessert wurde.

d. Eine Sachverständige aus Osteuropa | hatten | die Investoren | in der Planungsphase | eigenständig hinzugezogen, | aber | im Nachhinein | fragte | sich | so mancher Gutachter, | wieso, | da | das Ergebnis | augenscheinlich | nicht | verbessert wurde.

26. a. Ein Redakteur der Tageszeitung | hatte | die maskierten Aktivisten | zu einer geheimen Videokonferenz | eingeladen, | aber | niemand | konnte | überzeugend | begründen, | wieso, | nachdem | das Vorhaben | unbeabsichtigterweise | der Öffentlichkeit | bekannt geworden war.

b. Einen Redakteur der Tageszeitung | hatten | die maskierten Aktivisten | zu einer geheimen Videokonferenz | eingeladen, | aber | niemand | konnte | überzeugend | begründen, | wieso, | nachdem | das Vorhaben | unbeabsichtigterweise | der Öffentlichkeit | bekannt geworden war.

c. Eine Redakteurin der Tageszeitung | hatte | die maskierten Aktivisten | zu einer geheimen Videokonferenz | eingeladen, | aber | niemand | konnte | überzeugend | begründen, | wieso, | nachdem | das Vorhaben | unbeabsichtigterweise | der Öffentlichkeit | bekannt geworden war.

d. Eine Redakteurin der Tageszeitung | hatten | die maskierten Aktivisten | zu einer geheimen Videokonferenz | eingeladen, | aber | niemand | konnte | überzeugend | begründen, | wieso, | nachdem | das Vorhaben | unbeabsichtigterweise | der Öffentlichkeit | bekannt geworden war.

27. a. Ein Spion des Inlandsgeheimdienstes | hatte | die Informanten | im Vorfeld der Verhandlungen | enttarnt, | aber | nicht einmal Experten | wussten | letztlich | zu sagen, | wie, | bis | irgendwann | eine Reinigungskraft | im Schutz der Anonymität | den entscheidenden Hinweis gab.

b. Einen Spion des Inlandsgeheimdienstes | hatten | die Informanten | im Vorfeld der Verhandlungen | enttarnt, | aber | nicht einmal Experten | wussten | letztlich | zu sagen, | wie, | bis | irgendwann | eine Reinigungskraft | im Schutz der Anonymität | den entscheidenden Hinweis gab.

c. Eine Spionin des Inlandsgeheimdienstes | hatte | die Informanten | im Vorfeld der Verhandlungen | enttarnt, | aber | nicht einmal Experten | wussten | letztlich | zu sagen, | wie, | bis | irgendwann | eine Reinigungskraft | im Schutz der Anonymität | den entscheidenden Hinweis gab.

d. Eine Spionin des Inlandsgeheimdienstes | hatten | die Informanten | im Vorfeld der Verhandlungen | enttarnt, | aber | nicht einmal Experten |

wussten | letztlich | zu sagen, | wie, | bis | irgendwann | eine Reinigungskraft | im Schutz der Anonymität | den entscheidenden Hinweis gab.

28. a. Ein Dolmetscher des Botschafters | hatte | die Gastgeber | während der Begrüßungszeremonie | empfindlich gekränkt, | aber | damals | konnte | niemand | nachvollziehen, | womit, | obwohl | die kulturellen Gepflogenheiten | der jeweils anderen Seite | auf jeden Fall | hinreichend bekannt waren.
    b. Einen Dolmetscher des Botschafters | hatten | die Gastgeber | während der Begrüßungszeremonie | empfindlich gekränkt, | aber | damals | konnte | niemand | nachvollziehen, | womit, | obwohl | die kulturellen Gepflogenheiten | der jeweils anderen Seite | auf jeden Fall | hinreichend bekannt waren.
    c. Eine Dolmetscherin des Botschafters | hatte | die Gastgeber | während der Begrüßungszeremonie | empfindlich gekränkt, | aber | damals | konnte | niemand | nachvollziehen, | womit, | obwohl | die kulturellen Gepflogenheiten | der jeweils anderen Seite | auf jeden Fall | hinreichend bekannt waren.
    d. Eine Dolmetscherin des Botschafters | hatten | die Gastgeber | während der Begrüßungszeremonie | empfindlich gekränkt, | aber | damals | konnte | niemand | nachvollziehen, | womit, | obwohl | die kulturellen Gepflogenheiten | der jeweils anderen Seite | auf jeden Fall | hinreichend bekannt waren.

29. a. Ein Angestellter des städtischen Verkehrsunternehmens | hatte | die Fahrgäste | mit unverschämten Äußerungen | belästigt, | aber | das Team | von Soziologen | konnte | nicht | erklären, | wieso, | sodass | der Zwischenfall | für die Wissenschaft | bis heute | rätselhaft bleibt.
    b. Einen Angestellten des städtischen Verkehrsunternehmens | hatten | die Fahrgäste | mit unverschämten Äußerungen | belästigt, | aber | das Team | von Soziologen | konnte | nicht | erklären, | wieso, | sodass | der Zwischenfall | für die Wissenschaft | bis heute | rätselhaft bleibt.
    c. Eine Angestellte des städtischen Verkehrsunternehmens | hatte | die Fahrgäste | mit unverschämten Äußerungen | belästigt, | aber | das Team | von Soziologen | konnte | nicht | erklären, | wieso, | sodass | der Zwischenfall | für die Wissenschaft | bis heute | rätselhaft bleibt.
    d. Eine Angestellte des städtischen Verkehrsunternehmens | hatten | die Fahrgäste | mit unverschämten Äußerungen | belästigt, | aber | das Team | von Soziologen | konnte | nicht | erklären, | wieso, | sodass | der Zwischenfall | für die Wissenschaft | bis heute | rätselhaft bleibt.

30. a. Ein Aufseher des Gefängnisses | hatte | die verdächtigen Häftlinge | durch ein erfundenes Alibi | gedeckt, | aber | keinem der Beteiligten | war | damals | zu entlocken, | wieso, | denn | eine Aussage | hätte | wohl | gegen die Ehre verstoßen.
    b. Einen Aufseher des Gefängnisses | hatten | die verdächtigen Häftlinge | durch ein erfundenes Alibi | gedeckt, | aber | keinem der Beteiligten | war | damals | zu entlocken, | wieso, | denn | eine Aussage | hätte | wohl | gegen die Ehre verstoßen.
    c. Eine Aufseherin des Gefängnisses | hatte | die verdächtigen Häftlinge | durch ein erfundenes Alibi | gedeckt, | aber | keinem der Beteiligten | war | damals | zu entlocken, | wieso, | denn | eine Aussage | hätte | wohl | gegen die Ehre verstoßen.
    d. Eine Aufseherin des Gefängnisses | hatten | die verdächtigen Häftlinge | durch ein erfundenes Alibi | gedeckt, | aber | keinem der Beteiligten | war | damals | zu entlocken, | wieso, | denn | eine Aussage | hätte | wohl | gegen die Ehre verstoßen.

31. a. Ein Mitarbeiter der maroden Firma | hatte | die Geschäftsführer | in das raffinierte Veruntreuungssystem | eingeweiht, | aber | es | herrscht |

Uneinigkeit | darüber, | wann, | denn | von den belastenden Dokumenten | trägt | keines | ein Datum.

b. Einen Mitarbeiter der maroden Firma | hatten | die Geschäftsführer | in das raffinierte Veruntreuungssystem | eingeweiht, | aber | es | herrscht | Uneinigkeit | darüber, | wann, | denn | von den belastenden Dokumenten | trägt | keines | ein Datum.

c. Eine Mitarbeiterin der maroden Firma | hatte | die Geschäftsführer | in das raffinierte Veruntreuungssystem | eingeweiht, | aber | es | herrscht | Uneinigkeit | darüber, | wann, | denn | von den belastenden Dokumenten | trägt | keines | ein Datum.

d. Eine Mitarbeiterin der maroden Firma | hatten | die Geschäftsführer | in das raffinierte Veruntreuungssystem | eingeweiht, | aber | es | herrscht | Uneinigkeit | darüber, | wann, | denn | von den belastenden Dokumenten | trägt | keines | ein Datum.

32. a. Ein Geschworener des Gerichts | hatte | die beiden Angeklagten | trotz richterlicher Verwarnung | direkt angesprochen, | aber | niemand im Saal | verstand | wohl | so recht, | weshalb, | bevor | die Verhandlung | überraschend | auf unbestimmte Zeit | vertagt wurde.

b. Einen Geschworenen des Gerichts | hatten | die beiden Angeklagten | trotz richterlicher Verwarnung | direkt angesprochen, | aber | niemand im Saal | verstand | wohl | so recht, | weshalb, | bevor | die Verhandlung | überraschend | auf unbestimmte Zeit | vertagt wurde.

c. Eine Geschworene des Gerichts | hatte | die beiden Angeklagten | trotz richterlicher Verwarnung | direkt angesprochen, | aber | niemand im Saal | verstand | wohl | so recht, | weshalb, | bevor | die Verhandlung | überraschend | auf unbestimmte Zeit | vertagt wurde.

d. Eine Geschworene des Gerichts | hatten | die beiden Angeklagten | trotz richterlicher Verwarnung | direkt angesprochen, | aber | niemand im Saal | verstand | wohl | so recht, | weshalb, | bevor | die Verhandlung | überraschend | auf unbestimmte Zeit | vertagt wurde.
